# Tissue-regenerative potential of the secretome of γ-irradiated peripheral blood mononuclear cells is mediated via TNFRSF1B-induced necroptosis

**DOI:** 10.1038/s41419-019-1974-6

**Published:** 2019-09-30

**Authors:** Elisabeth Simader, Lucian Beer, Maria Laggner, Vera Vorstandlechner, Alfred Gugerell, Michael Erb, Polina Kalinina, Dragan Copic, Doris Moser, Andreas Spittler, Erwin Tschachler, Hendrik Jan Ankersmit, Michael Mildner

**Affiliations:** 1 Department of Internal Medicine III, Division of Rheumatology, Medical University of Vienna, Vienna, Austria; 2 Division of Thoracic Surgery, Medical University of Vienna, Vienna, Austria; 3 FFG Project 852748 “APOSEC“, Medical University of Vienna, Vienna, Austria; 4 Department of Biomedical Imaging and Image-guided Therapy, Medical University of Vienna, Vienna, Austria; 5 Department of Radiology and Cancer Research UK Cambridge Center, Cambridge, CB2 0QQ UK; 6 Vienna Business Agency Project 2343727 “APOSEC to clinic”, Medical University Vienna, Vienna, Austria; 7 Synlab Analytics and Services Switzerland AG, Birsfelden, Switzerland; 8 Research Division of Biology and Pathobiology of the SkinDepartment of Dermatology, Research Division of Biology and Pathobiology of the Skin, Medical University of Vienna, Vienna, Austria; 9 Division of Oral and Maxillofacial Surgery, Medical University of Vienna, Vienna, Austria; 10 Research Laboratories, Core Facility Flow Cytometry, Medical University of Vienna, Vienna, Austria

**Keywords:** Cell biology, Necroptosis

## Abstract

Peripheral blood mononuclear cells (PBMCs) have been shown to produce and release a plethora of pro-angiogenetic factors in response to γ-irradiation, partially accounting for their tissue-regenerative capacity. Here, we investigated whether a certain cell subtype of PBMCs is responsible for this effect, and whether the type of cell death affects the pro-angiogenic potential of bioactive molecules released by γ-irradiated PBMCs. PBMCs and PBMC subpopulations, including CD4^+^ and CD8^+^ T cells, B cells, monocytes, and natural killer cells, were isolated and subjected to high-dose γ-irradiation. Transcriptome analysis revealed subpopulation-specific responses to γ-irradiation with distinct activation of pro-angiogenic pathways, cytokine production, and death receptor signalling. Analysis of the proteins released showed that interactions of the subsets are important for the generation of a pro-angiogenic secretome. This result was confirmed at the functional level by the finding that the secretome of γ-irradiated PBMCs displayed higher pro-angiogenic activity in an aortic ring assay. Scanning electron microscopy and image stream analysis of γ-irradiated PBMCs revealed distinct morphological changes, indicative for apoptotic and necroptotic cell death. While inhibition of apoptosis had no effect on the pro-angiogenic activity of the secretome, inhibiting necroptosis in stressed PBMCs abolished blood vessel sprouting. Mechanistically, we identified tumor necrosis factor (TNF) receptor superfamily member 1B as the main driver of necroptosis in response to γ-irradiation in PBMCs, which was most likely mediated via membrane-bound TNF-α. In conclusion, our study demonstrates that the pro-angiogenic activity of the secretome of γ-irradiated PBMCs requires interplay of different PBMC subpopulations. Furthermore, we show that TNF-dependent necroptosis is an indispensable molecular process for conferring tissue-regenerative activity and for the pro-angiogenic potential of the PBMC secretome. These findings contribute to a better understanding of secretome-based therapies in regenerative medicine.

## Introduction

Regenerative medicine, aiming at restoring damaged tissues and organs, has become an emerging branch of translational research in the last century worldwide^1^. However, despite major advances in drug therapies, surgical interventions, and organ transplantation, regeneration of injured organs still remains a major obstacle^2^. A promising new therapeutic avenue may be offered by stem cell-based therapies, on which numerous pre-clinical studies, investigating their efficacy and mechanisms have been conducted^3–6^. Unfortunately, translation of experimental in vitro studies or animal models to the patient has been shown to be extremely difficult if not impossible^7^. In addition, an increasing number of studies suggests that not stem cells themselves, but rather the factors released from stem cells are important and sufficient to promote tissue regeneration^8,9^.

In 2005, Thum et al.^10^ speculated that stem cells undergo apoptosis while being processed for clinical applications and thus induce immunomodulatory and tissue-regenerative effects. In addition, the authors doubted the uniqueness of stem cells and suggested that any other nucleated apoptotic cell type would exhibit tissue-regenerative features^10^. The first study providing evidence for tissue repair by stressed peripheral blood mononuclear cells (PBMCs) was performed by Ankersmit et al.^11^. Enhanced regeneration was observed in acute myocardial infarction (AMI) by applying γ-irradiated PBMC suspensions intravenously. In subsequent years, we were able to show that the application of the PBMC secretome alone causes tissue repair in AMI^12–14^, stroke^15^, spinal cord^16^, and skin wounds^17–19^, in small and clinically relevant large animals. Although a previous study from our group suggested that γ-irradiation is able to induce apoptosis and necroptosis^20^, a contribution of necroptosis to tissue regeneration by the release of paracrine factors has not been investigated so far.

In contrast to necrosis, an uncontrolled form of cell death, apoptosis had already been described as a well-controlled form of programmed cell death decades ago^21^. Later, also a programmed form of necrosis, termed necroptosis^22,23^. The two forms of programmed cell death differ morphologically as well as mechanistically from one another. Morphologically, apoptosis is characterized by karyorrhexis, pyknosis, and blebbing of the plasma membrane^22^. By contrast, necroptotic cells exhibit translucent cytoplasms, oncosis, and permeabilization of the lysosomal and plasma membranes, while nuclei remain intact^22,23^. Instead of caspase activation, necroptosis involves receptor-interacting protein kinase‐1 (RIPK1), RIPK3, and mixed lineage kinase domain‐like (MLKL) activation^24^. Tumor necrosis factor-α (TNF-α) is one of the best characterized inducers of apoptosis, activating the caspase-8 signalling cascade. However, due to partially overlapping upstream signalling elements, TNF can also activate the necroptotic pathway, which is favored by impaired caspase activity^23^. Whereas the role of necroptosis in several pathological conditions, including atherosclerosis^25^, myocardial infarction^26^, traumatic brain injury^27^, and infections^28^ have been investigated so far, the effects of the necroptotic cells on surrounding tissues remains poorly understood.

**Fig. 1:**
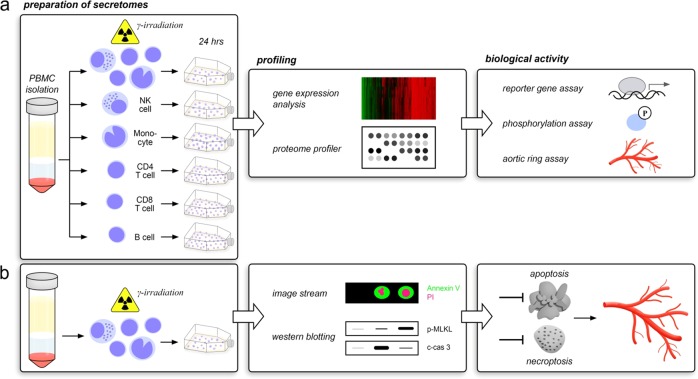
Schematic illustration of experimental approach. **a** To determine respective contributions of PBMC subsets to tissue-regenerative properties of secreted factors, secretomes obtained from PBMCs, and subpopulations were compared by gene expression analysis and proteome profiler. Biological activities were analyzed by functional assays using reporter gene constructs, protein phosphorylation, and aortic ring-assisted endothelial cell sprouting. **b** To assess whether the type of programmed cell death affects pro-angiogenic potency of PBMC secretome, dying cells were categorized by image stream and activated intracellular signalling cascades were investigated by Western blotting. Eventually, pro-angiogenic property of apoptotic and necroptotic PBMC secretomes was compared by aortic ring sprouting assay

Although several biological effects of paracrine factors released from stressed PBMCs have already been investigated, the mechanisms by which these factors exert their pro-angiogenic and tissue-regenerative activities have not been fully elucidated so far^17^. In the current study, we therefore addressed two major questions: (1) is the pro-angiogenic potential of the secretome of γ-irradiated PBMC cell type-dependent and (2) does the type of programmed cell death contribute to the pro-angiogenic property of γ-irradiated PBMC secretome (Fig. 1).

## Materials and methods

### Ethics vote

Heparinized blood samples for PBMC isolation were obtained from healthy volunteers at the Department for Blood Transfusion Medicine of the Medical University of Vienna (ethics committee vote: EK-Nr 1539/2017). All donors provided informed written consent. For ex vivo angiogenesis experiments, mouse experiments were performed according to recent Austrian guidelines for the use and care of laboratory animals and approved by the Animal Research Committee of the Medical University of Vienna (Protocol No. 190097/2015/9).

### Isolation of PBMCs and PBMC subsets and production of the secretomes

Cell secretomes were produced as described previously^27^. Briefly, PBMCs were isolated using density gradient centrifugation via Ficoll-Paque PLUS (GE Healthcare Bio-Sciences AB, Sweden). Heparinized blood was diluted with phosphate-buffered saline (PBS, Gibco by Life Technologies, Carlsbad, CA, USA) and layered carefully over Ficoll-Paque PLUS. After centrifugation (800 × *g*, 15 min, room temperature, with slow acceleration and deceleration), buffy coat containing PBMCs was enriched at the interface between Ficoll-Paque PLUS and plasma. For purification of monocytes (CD14), natural killer cells (CD56), CD4^+^ T cells (CD4), CD8^+^ T cells (CD8), and B cells (CD19), magnetic microbeads (Miltenyi, Bergisch Gladbach, Germany) against the respective cell surface epitope were used to enrich cells by Auto-Macs Pro technology (Miltenyi) according to the manufacturer’s protocol. Purity of isolated cells was confirmed by flow cytometry and ranged from 93 to 99% (Supplementary Fig. 1). Whole PBMCs and purified cell subsets were resuspended in CellGro serum-free medium (CellGenix, Freiburg, Germany), irradiated, and cultivated for 24 h at a concentration of 25 × 10^6^ cells/ml in the same medium. γ-Irradiation of isolated PBMCs and purified PBMC subsets with Cesium-137 (60 Gy) was conducted as described previously^29^. To evaluate dose-dependent effects of γ-irradiation, PBMCs were irradiated with 0.9, 1.9, 3.75, 7.5, 15, 30, and 60 Gy. For inhibition of apoptosis and necroptosis, 20 µM zVAD and 100 μM necrostatin-1 (both Sellekchem, Munich, Germany) were added immediately after irradiation. After 24 h of incubation, supernatants were collected by centrifugation (400 × *g*, 9 min) and stored at −20 °C. Cells were used for flow cytometric analysis and lysed for protein and messenger RNA (mRNA) analyses as described below.

### Imaging flow cytometry analysis

Imaging flow cytometry analysis (Amnis ImageStreamX Mk II, Luminex Corp., Seattle, WA) was performed according to a published protocol using Annexin-V-FLUOS Staining Kit (Roche, Basel, Switzerland) according to the manufacturer’s instruction^30^.

### Scanning electron microscopy

For scanning electron microscopy (SEM), PBMCs were either irradiated with 60 Gy or left untreated, washed twice with PBS, fixed in Karnovsky’s fixative (2% paraformaldehyde, 2.5% glutaraldehyde in 0.1 M phosphate buffer (pH 7.4); Morphisto, Frankfurt am Main, Germany), dehydrated, and dried with hexamethyldisilazane (HMDS, Sigma-Aldrich, Taufkirchen, Germany). Samples were fixed to specimen mounts with double-faced adhesive carbon tape, gold sputtered (Sputter Coater, ACE200, Leica Microsystems, Wetzlar, Germany), and examined by a SEM (JSM 6310, Jeol Ltd®, Japan) with an acceleration voltage set to 15 kV.

### Western blot analysis

Cells for Western blot analysis were lysed in Lämmli Buffer (Bio-Rad, Hercules, CA, USA) with protease inhibitors (Thermo Fisher, Waltham, MA, USA) and sodium orthovanadate (Sigma Aldrich, St. Louis, MO, USA) according to the manufacturer’s protocol. Thirty micrograms of total protein were separated on ExcelGels (GE Healthcare) and transferred onto nitrocellulose membranes (Bio-Rad). After blocking, membranes were incubated with primary antibodies [cleaved caspase-3 antibody (0.5 µg/ml, #MAB835; R&D Systems, Minneapolis, MN, USA), phospho-RIPKs 1 (1:100, #65746; Cell Signalling Technology, Cambridge, UK), phospho-RIPK3 (1:200, #ab209384; Abcam, Cambridge, UK), phospho-MLKL (1:500, #91689; Cell Signalling Technology, Cambridge, UK), or glyceraldehyde 3-phosphate dehydrogenase (1:2000, #2118; Cell Signalling Technology, TNF (1 µg/ml, R&D Systems)] overnight at 4 °C. After further incubation with horseradish-conjugated goat-anti-rabbit antibody (1:10,000, #170-6515; Bio-Rad, Hercules, CA, USA), secondary antibodies were visualized with Supersignal West Dura (Thermo Fisher, Waltham, MA, USA) and signals were detected using ChemiDoc System (Bio-Rad). For blocking of the TNF antibody, 1 µg TNF antibody was pre-incubated with 10 µg recombinant TNF (R&D Systems) for 4 h at 4 °C:

### TNF receptor blockade

PBMCs were treated with zVAD and neutralizing antibodies against TNF receptor superfamily member 1A (TNFRSF1A), TNFRSF1B (both 1 µg/ml, R&D Systems), or both were added. Cell lysates were obtained 24 h after irradiation.

### Total RNA isolation

Total RNA was isolated from PBMCs and PBMC subsets immediately after cell purification as well as 24 h after irradiation with 60 Gy using Trizol® Reagent (Invitrogen, Carlsbad, CA) according to the manufacturer’s instructions. Total RNA was quantified using NanoDrop-1000 spectrophotometer (Peglab, Erlangen, Germany) and RNA quality was assessed by Agilent 2100 Bioanalyzer (Agilent Technologies, Santa Clara, CA, USA). All RNA samples used in further procedures displayed an RNA integrity score between 6.2 and 10.

### Microarray analysis

Microarray analysis was carried out at the Genomics Core Facility at the Medical University of Vienna (Vienna, Austria) using Affymetrix Human Transcriptome Array 2.0 (Affymetrix part of Thermo Fisher Scientific Inc.) according to MIAME guidelines^31^. Data were analyzed using GeneSpring Version 15.0 software (Agilent). First, raw data were log 2 transformed, normalized by quintile normalization, and baseline transformed. Thereafter, a filtering step was performed in order to reduce the number of multiple hypotheses and to obtain only genes for which at least 75% of the values in one sample (0 h vs. irradiated) were above the 60th percentile of the average expression value^32^. Moderated paired *t* test was used to identify differentially expressed mRNA with a fold change (FC) ≤ −2 and ≥2, respectively. *P* values were corrected for multiplicity by applying Benjamini–Hochberg adjustment with a false discovery rate (FDR) <5%. mRNA clustering was performed with GeneSpring software using Euclidean distance metric and complete average-linkage clustering. Microarray data were published on NCBI Gene expression omnibus at https://www.ncbi.nlm.nih.gov/geo/query/acc.cgi?acc=GSE127982 (GEO Accession number GSE127982). Full access is granted using the password: qnuxcigibxmdfcf.

### Gene ontology and pathway analysis

In order to evaluate biological functions of differentially expressed genes in response to irradiation, we categorized them using the WEB-based Gene Stet Analysis Toolkit (WebGestalt)^33^. Gene ontology (GO)-term enrichment analysis was performed to identify biological processes that were enriched (geneontology.org). In addition, pathway analysis was performed using the Kyoto Encyclopedia of Genes and Genomes (KEGG) annotation list^33^. Benjamini–Hochberg method for multiple testing with a significance level of *p* ≤ 0.05 and FDR < 5% was applied for both analyses. Activated canonical pathways were identified using Ingenuity Pathway Analysis (Qiagen, Hilden, Germany) with mRNAs displaying an average FC >3 between 60-Gy-irradiated and freshly isolated samples^34,35^.

### Proteome profiler

Secretomes obtained from PBMCs and PBMC subsets were analyzed using the commercially available Proteome Profiler XL Cytokine Array and Human Apoptosis Array (R&D Systems) according to the manufacturer’s instructions. Arrays were analyzed with the ChemiDoc system as described above.

### Aortic ring assays

Male C57BL/6 mice were purchased from The Jackson Laboratory (Distributor Charles River, Sulzfeld, Germany) and housed at the Center for Biomedical Research of the Medical University of Vienna (Vienna, Austria). Mice were sacrificed via cervical dislocation and aortas were excised and sliced in 1-mm-thick rings (Supplementary Fig. 2). The aortic ring assay was performed according to a published protocol with minor alterations^16^. Aortic rings were sandwiched in a fibrin matrix composed of fibrinogen (2 mg/ml, Merck Millipore, Burlington, MA, USA), aprotinin (43.3 µg/ml, Sigma-Aldrich, St. Louis, MO, USA), and thrombin (0.6 U/ml, Sigma-Aldrich) as described previousy^29^. Sandwiched aortas were equilibrated with M199 medium, supplemented with 100 μg/ml streptomycin, 4 mM l-glutamine, 100 U penicillin (all from Gibco), 250 ng/ml amphotericin B (Fisher Bioreagents, Fisher Scientific, Waltham, MA, USA), and 10% fetal bovine serum (PAA Laboratories, Pasching, Austria), for 45 min. After equilibration, the medium was removed and supernatants of PBMCs and PBMC subfractions were diluted in M199 medium corresponding to a final concentration of 4 × 10^6^ cells/ml. Aortas were cultured for 3 days. For some sprouting assays, PBMC-derived secretomes generated with the addition of 20 µM zVAD and 100 μM necrostatin-1 directly after irradiation were investigated. Secretomes of PBMCs with zVAD and necrostatin-1 added immediately before starting the sprouting assay were included as controls. Ultimately, calcein dye (Thermo Fisher, Waltham, MA, USA) was added to label viable cells. Sprouts were photographed by Olympus IX83 scanning microscope (Olympus, Tokyo, Japan) and visualized with cellSens Imaging Software (Olympus, Tokyo, Japan). Sprouting areas were calculated using the ImageJ software version 1.48v (Wayne Rasband, National Institutes of Health, Bethesda, MD, USA).

### Tube formation assays

Primary human umbilical vein endothelial cells (HUVECs) were cultivated in endothelial cell growth medium (EGM-2, Lonza, Basel, Switzerland). Before starting the tube formation experiment, cells were subsequently starved with basal medium (EBM-2, Lonza, without growth factors) supplemented with 2% FBS (Gibco) for 12 h and without serum for 3 h. µ-Slide angiogenesis tissue culture slides (Ibidi USA Inc., Fitchburg, WI, USA) were filled with growth factor reduced Matrigel matrix (Corning, Corning, NY, USA), according to manufacturer’s protocol. A total of 1 × 10^4^ cells/well were seeded and treated with PBMC-derived secretome corresponding to a final concentration of 4 × 10^6^ cells/ml or medium alone. PBMC secretomes generated with the addition of 20 µM zVAD and 100 mM necrostatin-1 directly after irradiation were also investigated. Secretomes of PBMCs with zVAD and necrostatin-1 added immediately before starting the tube formation assay were included as controls. After 3 h of stimulation, microphotographs were taken and the number of nodes, junctions, and branches were analyzed via Angiogenesis Analyzer ImageJ plugin using default settings (Wayne Rasband, National Institutes of Health, USA).

### Reporter gene assays and potency assays

Reporter gene assays for activator protein-1 (AP-1), nuclear factor ‘kappa-light-chain-enhancer’ of activated B cells (NF-κB), and heat-shock protein 27 (HSP-27) developed at Synlab Pharma Institute AG (Bern, Switzerland) were used to compare the potential of the different secretomes to activate these pathways. Human neuroblastoma SH-SY5Y cells were cultured in Ham’s F12/MEM (50:50) Glutamax (Gibco) supplemented with 1 µg/ml puromycin, 2 mM l-glutamine, and non-essential amino acid solution (all from Sigma-Aldrich, St. Louis, MO, USA), 15% fetal bovine serum and SH-SY5Y cells were stably transfected with a firefly luciferase transcriptionally regulated by AP-1 promoter. Cells were seeded in 96-well plates at a concentration of 20,000 cells per well and stimulated with secretomes of γ-irradiated monocyte supernatant and PBMCs, both pooled from four donors. To evaluate reporter activity, SteadyGlo (Promega, Fitchburg, WI, USA) was added and luminescence was measured via luminescence reader (EnVision, Perkin-Elmer or Centro LB960, Berthold). To quantify phosphorylation of HSP-27 at Ser82, adenocarcinomic human alveolar basal epithelial cells (A549) were treated with supernatants for 30 min, fixed, and permeabilized. After sequential addition of antibodies directed against the phosphorylated form of HSP-27 and peroxidase-conjugated antibody, chemiluminescent signals were measured with the luminescence reader (EnVision, Perkin-Elmer or Centro LB960, Berthold) and relative potency was calculated with the PLA software (Stegmann Systems GmbH, Rodgau, Germany).

### Enzyme-linked immunosorbent assay

TNF-α (R&D Systems) and lymphotoxin A (LTA; R&D Systems) were quantified by enzyme-linked immunosorbent assay according to the manufacturer’s instructions.

### Graphical overview

The methodological approach was designed using the InDesign CS software (version 7.0, Adobe Systems Inc., San Jose, CA, USA) and is shown in Fig. 1.

### Statistical analysis

Data were analyzed using GraphPad Prism 6 software (GraphPad Software Inc., La Jolla, CA, USA) and IBM SPSS Statistics version 23 (SPSS Inc., Chicago, IL, USA). Two-tailed Student’s *t* test was used to compare parametric variables and stated as arithmetic mean ± standard deviation (SD). One-way analysis of variance with Bonferroni post hoc test or Kruskal–Wallis with Dunn’s post hoc test was used according to data distribution. Aberrations were excluded according to the Gibbs outlier test. *P* values below 0.05 were considered statistically significant and are marked with asterisks.

## Results

### γ-Irradiation differentially affects transcriptional profiles of PBMCs and purified cell subsets

**Fig. 2:**
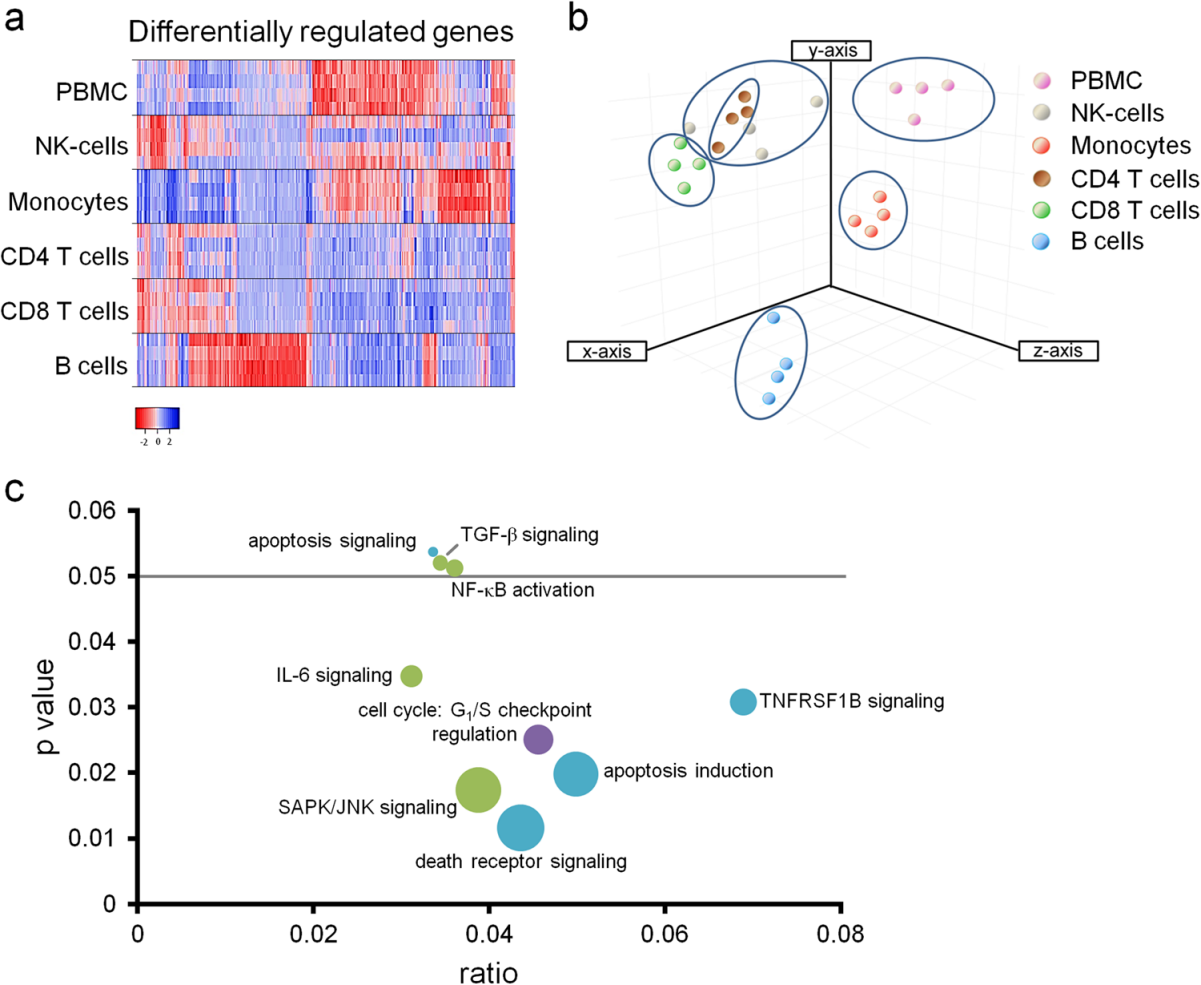
γ-Radiation differentially affects gene expression of PBMCs and purified cell subsets. **a** Heat map showing expression values of 910 transcripts differentially expressed between non-irradiated cells and cells 24 h after irradiation. Fold expression ranged from −2.7 (red, downregulation) to 2.7 (blue, upregulation). Each cell subset exhibited a unique expression pattern. **b** Principal component analysis (PCA) of global gene expression pattern clearly showed clustering of cell subsets, indicating a strong homogeneity within each cell fraction and different donors, but heterogeneity between all cell fractions. **c** Visualization of overrepresented GO terms in irradiated PBMCs is shown. Each node represents a biological process-specific term. Node size indicates *p* value, node color was selected to group terms according to their function (blue, apoptosis; green, pro-inflammatory signalling; purple, cell cycle). Ratios indicate relative amounts of activated genes per pathway. Among the most enriched terms were “TNFRSF1B signalling,” “apoptosis induction,” and “death receptor signalling.” *n* = 4 donors per group

To assess the impact of γ-irradiation on transcriptional networks of PBMCs and PBMC subsets, we conducted mRNA microarray analysis four different healthy volunteers with and without γ-irradiation. In total, 756 annotated genes were differentially expressed in PBMCs and PBMC subsets (Supplementary Table 1). Global gene expression analysis showed significant differences between stressed PBMCs and stressed purified cell types (Fig. 2a, b). Principal component analysis of global gene expression patterns showed a clear distinction between the different cell types except for natural killer (NK) and CD4^+^ T cells, which clustered together, suggesting high transcriptional similarity (Fig. 2b). Monocytes, B cells, and PBMCs displayed markedly distinct global gene expression patterns. Canonical pathway analysis of genes upregulated by γ-irradiation in PBMCs suggested activation of death receptors, upregulation of TNF receptor 2 (TNFRSF1B) signalling, and induction of apoptosis. Moreover, we identified activation of cytokine and cell signalling pathways, including NF-κB and the stress-activated protein kinase c-Jun-N-terminal kinase, both of which are linked to tissue-regenerative and angiogenic processes (Fig. 2c)^36–38^.

**Fig. 3:**
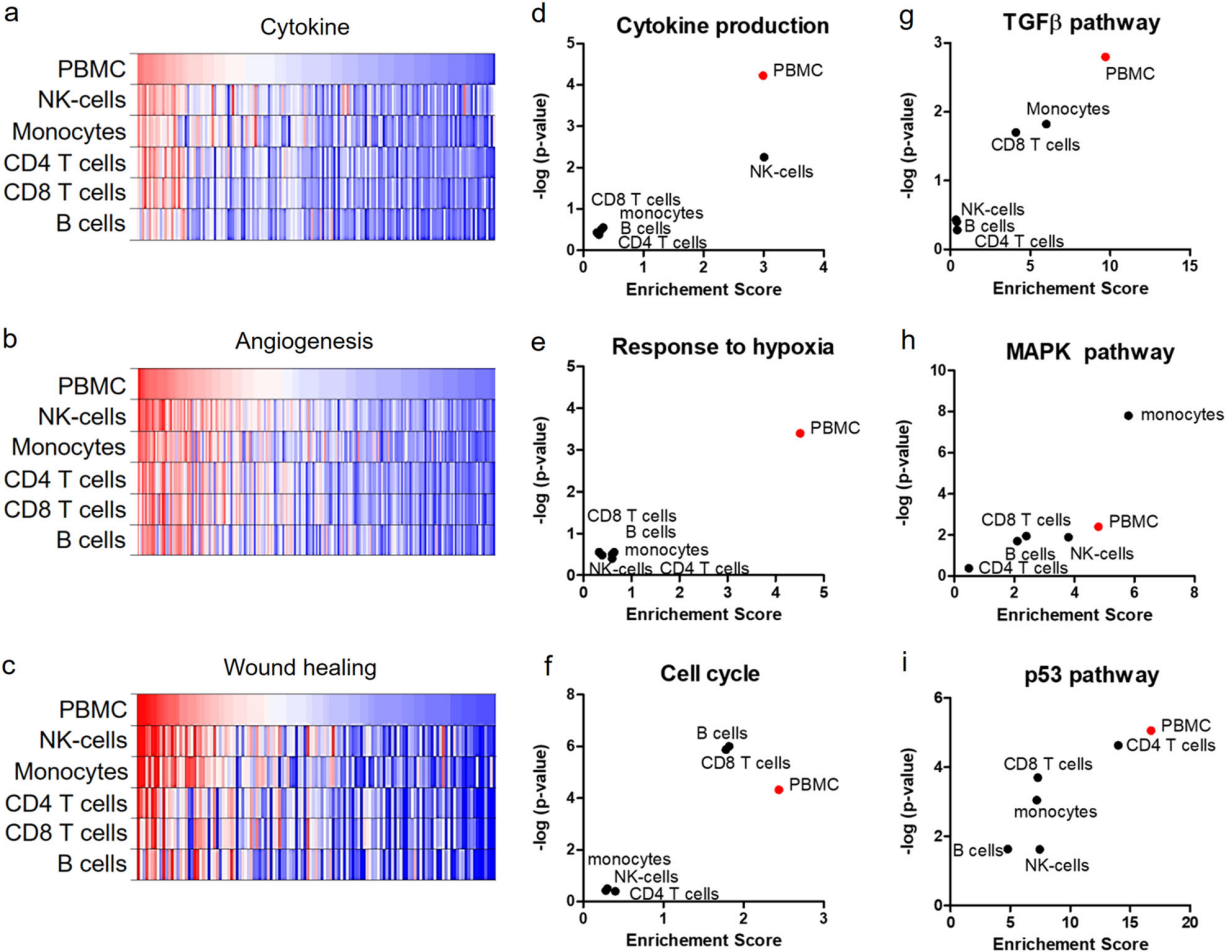
Tissue-regenerative potential varies between PBMCs and purified cell subsets. Genes associated with the biological process of **a** cytokine production (742 genes), **b** angiogenesis (509 genes), and **c** wound healing (361 genes) (mean values of four samples) revealed transcriptional heterogeneity between the different purified cells subsets and PBMCs. Expression values ranged from log(2) = 1 (red, low expression) to log(2) = 9 (blue, strong expression). GO-term enrichment analysis for **d** “cytokine production,” **e** ”response to hypoxia,” and **f** “cell cycle” as well as KEGG-pathway enrichment analysis for **g** “TGF-β”, **h** “MAPK”, and **i** “p53 pathway” are shown. Irradiated PBMCs (highlighted in red) uniformly enriched genes associated with these biological processes and pathways, while individual cell subsets showed a more heterogeneous pattern. The analysis was performed using *Webgestalt* platform. *P* values are represented as the minus base 10 log. *n* = 4 per group

To gain more detailed biological information on the pathways identified, we investigated expression profiles of key signalling molecules of these pathways in the different cell subsets. Selected genes involved in the processes of cytokine production (Fig. 3a and Supplementary Table 2), angiogenesis (Fig. 3b and Supplementary Table 3), and wound healing (Fig. 3c and Supplementary Table 4) displayed notable differences in their expression pattern in PBMCs compared to PBMC subsets. GO-term and KEGG-pathway analyses of genes induced by γ-irradiation (Fig. 3d–i) reflected the differences observed on transcriptional level also in a functional context. All selected biological functions, including cytokine production (Fig. 3d), response to hypoxia (Fig. 3e), and cell cycle (Fig. 3f), as well as the tumor growth factor-β (TGF-β) pathway (Fig. 3g), mitogen-activated protein kinase (MAPK) pathway (Fig. 3h), and p53 pathway (Fig. 3i) varied significantly between the different cell groups. While genes encoding cytokines were strongly enriched in PBMCs and NK cells (Fig. 3d), genes constituting the TGF-β signalling pathway were found mainly activated in PBMCs, CD14 monocytes, and CD8^+^ T cells (Fig. 3g). Genes associated with stress response (response to hypoxia) were exclusively enriched in irradiated PBMCs (Fig. 3e), while the MAPK pathway was most upregulated in monocytes (Fig. 3h). Cell cycle genes were enriched after irradiation in PBMCs as well as in B cells and CD8^+^ T cells (Fig. 3f), whereas genes involved in the p53 signalling were significantly enriched in all samples evaluated, showing strongest activation in PBMCs (Fig. 3i). In conclusion, results presented here indicate that various signalling pathways and biological processes are differentially regulated after γ-irradiation in the respective cellular subsets constituting PBMCs. Moreover, cell populations are in reciprocal relationships which, although mutually, differentially influence cellular signalling events in PBMCs.

### γ-Irradiated PBMC subpopulations synergistically induce blood vessel sprouting

**Fig. 4:**
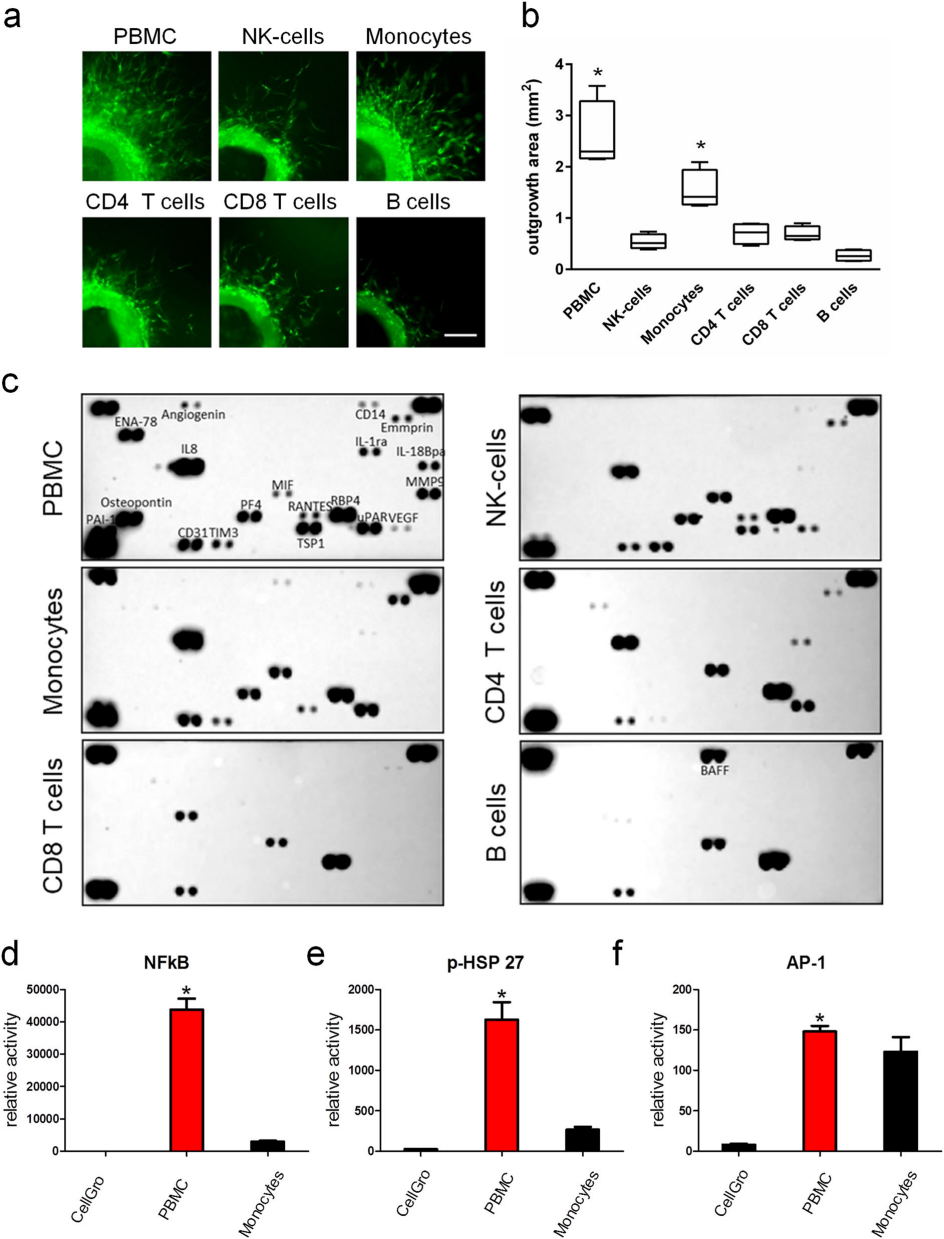
Blood vessel sprouting is synergistically induced by γ-irradiated PBMC subpopulations. **a** Representative images of calcein-labelled mouse aortic rings on day three of cultivation are shown. Scale bar, 250 µm. **b** Box plots of averaged outgrowth areas are shown. Whiskers indicate minimal and maximal values. Quantitative analysis showed a significant induction of sprouting blood vessels when adding the supernatant of PBMCs as well as the supernatant of purified monocytes. *P values <0.05 compared to CellGro. **c** Analysis of cytokines present in the different secretomes revealed that certain cytokines, including MMP-9, IL-18Bpa, osteopontin, ENA78, IL-8, RANTES, angiogenin, and IL-1ra, were exclusively detected in the supernatant of γ-irradiated PBMC. **d**–**f** Tissue-regenerative capacity of secretomes of γ-irradiated PBMCs and monocytes was further assessed using standardized reporter gene assays. NF-κB promoter activity and HSP-27 phosphorylation were strongly induced by PBMC supernatant, whereas AP-1 promotor was induced by PBMC and monocyte secretome. *P values <0.05 compared to CellGro. *n* = 4

As we have previously described a strong tissue-regenerative and pro-angiogenic activity of the secretome derived from γ-irradiated PBMCs^29,39^, and since our bioinformatics analysis revealed differential transcriptional signatures, we now asked whether a specific cell subtype of PBMCs would account for the observed effects. We therefore performed aortic ring assays with supernatants from γ-irradiated PBMCs, NK cells, monocytes, CD4^+^ T cells, CD8^+^ T cells, and B cells. As shown in Fig. 4a, b, strongest pro-angiogenic activity was observed in aortic rings cultured with the secretome of whole PBMCs. Intriguingly, monocytes displayed vessel sprouting-inducing capacity, which was higher compared to medium, yet compromised compared to that of the PBMC-derived secretome. Stimulation of aortic rings with secretomes derived from NK cells, CD4^+^ and CD8^+^ T cells, and B cells showed no increased pro-angiogenic effects compared to control medium (not shown) in our assay system (Fig. 4a, b). We furthermore sought to profile the specific protein signatures obtained from PBMCs and subsets. Analysis of cytokines revealed that certain cytokines, including matrix metallopeptidase-9, interleukin-18Bpa (IL-18Bpa), osteopontin, epithelial derived neutrophil attractant-78, IL-8, RANTES (regulated on activation, normal T cell expressed and secreted), angiogenin, and IL-1ra were exclusively detected in the supernatant of γ-irradiated PBMC (Fig. 4c, Supplementary Fig. 4). Since pro-angiogenic activity was unique to secretomes of PBMC and monocytes, we next compared the capability of these secretomes to activate signalling pathways known to be involved in tissue-regenerative and pro-angiogenic processes. Interestingly, NF-κB promoter activity (Fig. 4d) and HSP-27 phosphorylation (Fig. 4e) were strongly induced by PBMC-derived secretome, while being only moderately activated by the secretome obtained from irradiated monocytes. In contrast, both secretomes comparably activated the AP-1 promotor (Fig. 4f). Together, our data suggest that secretomes of PBMC subsets exhibit differential pro-angiogenic capacities and that a synergistic action of γ-irradiated PBMC subpopulations is necessary for efficient release of pro-angiogenic mediators.

### High-dose γ-irradiation induces apoptosis and necroptosis in PBMCs

**Fig. 5:**
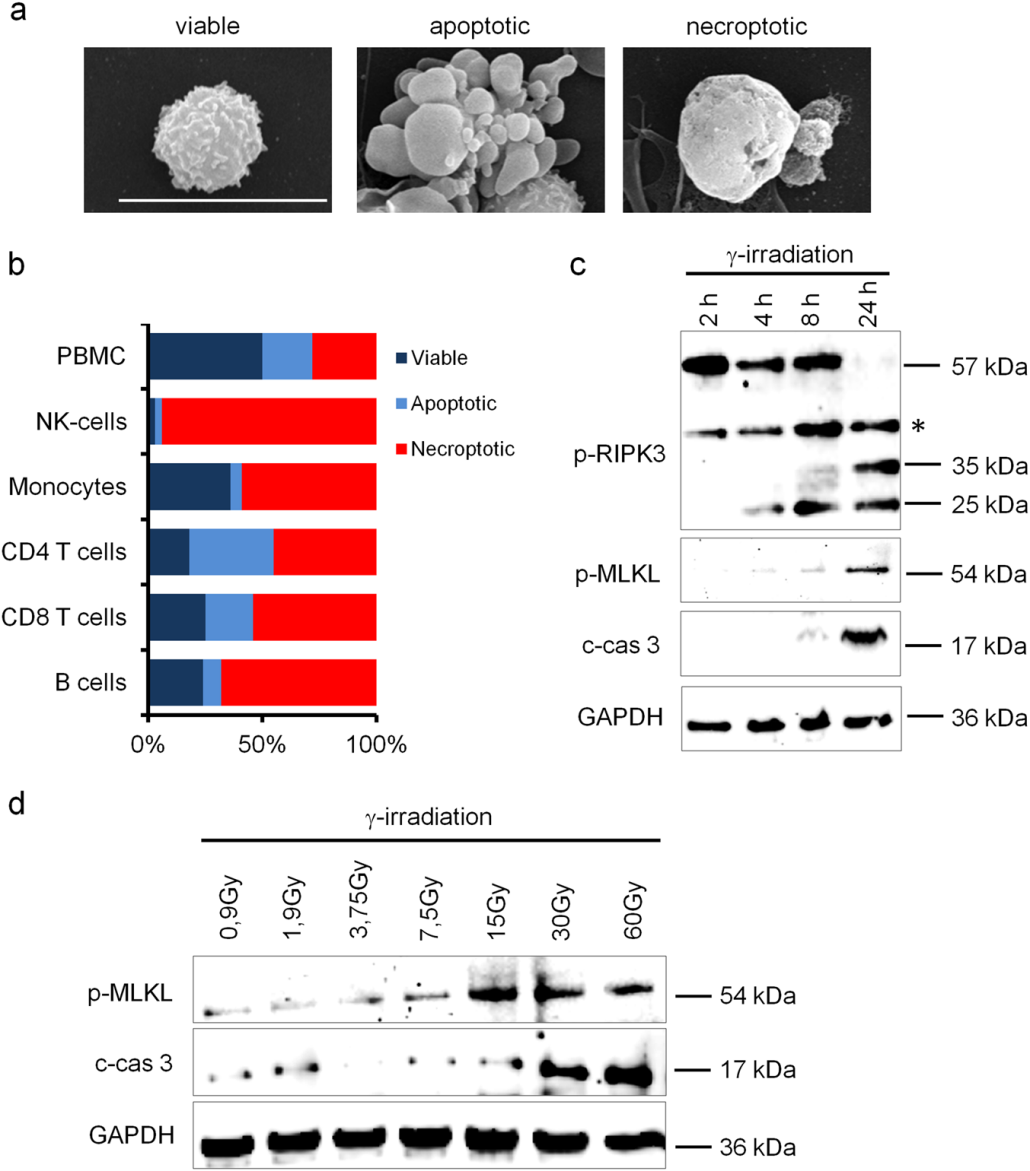
γ-irradiation differentially induces apoptosis and necroptosis in PBMC and purified cell subsets. **a** Representative scanning electron microscopy images of viable, apoptotic, and necroptotic cells 24 h after irradiation. Scale bar, 10 μm. **b** Relative amounts of viable, apoptotic, and necroptotic cells in PBMC and PBMC subpopulations. **c** Immunoblot analysis of receptor-interacting protein kinase-3 (p-RIPK3), pseudokinase mixed lineage kinase domain-like phosphorylation (p-MLKL), and cleaved caspase (c-cas 3) in PBMC shows a time-dependent induction 24 h after γ-irradiation. The position of an unidentified, most likely unspecific, band on the p-RIPK3 Western blot is indicated by an asterisk. **d** γ-Irradiation dose-dependently induces phosphorylation of MLKL and caspase-3 cleavage. *n* = 3

Since our bioinformatics analysis suggested an activation of death receptors and an involvement of TNF receptor signalling, we were interested which type of cell death is induced in PBMCs and PBMC subsets after γ-irradiation. We therefore assessed cellular morphology, indicative of the manner by which cells die, by SEM. Intriguingly, we found comparable number of cells showing morphological signs of either apoptosis or necroptosis in irradiated PBMCs (Fig. 5a). Interestingly, the levels of cells displaying either apoptotic or necroptotic features varied significantly between different populations (Fig. 5b). As already observed by electron microscopy, quantification of apoptotic and necroptotic cells in γ-irradiated PBMCs confirmed an almost equal abundance (22% apoptotic vs. 28% necroptotic) of both forms of controlled cell death. In contrast, most of NK cells were necroptotic (3% apoptotic vs. 94% necroptotic). Although around half of CD4^+^ and CD8^+^ T cells underwent necroptosis, the highest number of apoptotic cells were also detected in these populations (CD8^+^ T cells: 21% apoptotic vs. 54% necroptotic; CD4^+^ T cells: 37% apoptotic vs. 45% necroptotic). By contrast, B cells and monocytes displayed low amounts of apoptotic cells (B cells: 8% apoptotic vs. 68% necroptotic; monocytes: 5% apoptotic vs. 59% necroptotic). These data were further corroborated by an apoptosis protein array, showing that the induction of proteins involved in the apoptotic process was strongly induced in cell types that were mainly driven into apoptotic cell death (Supplementary Fig. 5). These results highlight the different susceptibilities of PBMC subsets to preferentially undergo apoptosis or necroptosis after high-dose γ-irradiation.

To assess kinetics and dose dependency of apoptosis and necroptosis induction after γ-irradiation on the molecular level, we evaluated cleavage of caspase-3 (c-cas 3) and phosphorylation of RIPK3 and MLKL, respectively (Fig. 5c, d). MLKL phosphorylation, indicative of induction of necroptosis, occurred in a dose-dependent manner, reaching its maximum at an irradiation dose of 15 Gy (Fig. 5d). Comparably, caspase-3 cleavage was induced by γ-irradiation starting from 30 Gy. For further characterization of the irradiation-induced programmed cell death, phosphorylation of RIPK3, MLKL, and c-cas 3 was assessed at different time points. While sustained phosphorylation of RIPK3 was detected starting from 2 h post irradiation, phosphorylation of MLKL and caspase-3 cleavage displayed highest levels 24 h post irradiation (Fig. 5c). Interestingly, we detected two smaller bands of 35 and 25 kDa in the phosphorylated (p)-RIPK3 Western blot. In contrast to γ-irradiation, induction of RIPK3 and MLKL phosphorylation by TNF-α and zVAD peaked as soon as 2 h after stimulation (Supplementary Fig. 6) and did not induce cleavage of p-RIPK3. Since MLKL phosphorylation occurs rapidly after induction of necroptosis, our finding that MLKL phosphorylation peaked 24 h after γ-irradiation suggests an indirect induction of necroptosis in PBMCs after exposure to γ-radiation.

### Pro-angiogenic capacity of PBMC secretome requires irradiation-induced necroptosis

**Fig. 6:**
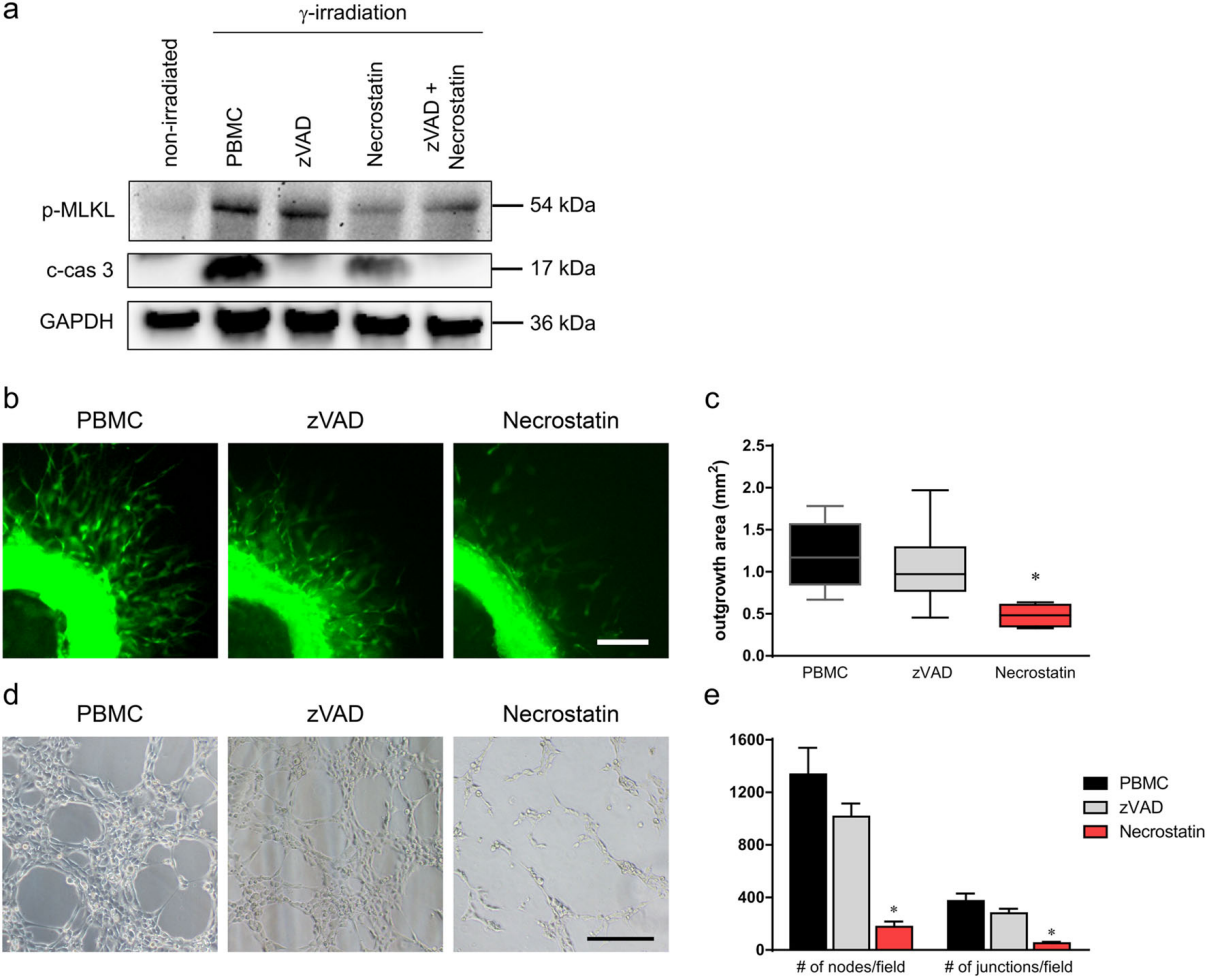
Induction of necroptosis is necessary for the pro-angiogenic capacity of the PBMC secretome. **a** Immunoblot analysis of phosphorylated MLKL and cleaved caspase (c-cas) 3 in non-irradiated and γ-irradiated PBMCs is shown. Irradiated PBMCs were cultivated with either zVAD, necrostatin-1, or both. **b** Representative images of murine aortic rings after 3 days of cultivation with conditioned medium from γ-irradiated PBMC treated with zVAD, necrostatin-1, or both. Viable cells were visualized with calcein (green). Scale bar, 200 µm. **c** Box plot diagrams of vessel areas are shown. Whiskers indicate minimal and maximal values. Necrostatin-1 added to irradiated PBMC significantly reduced sprout-inducing ability of the PBMC secretome, while addition of zVAD did not compromise pro-angiogenic potential. **d** Endothelial cells were incubated with the secretome of γ-irradiated PBMCs alone or treated with zVAD or necrostatin for 3 h after overnight starvation. The PBMC secretome treated with necrostatin-1 lead to significantly reduced tube formation. **e** Bar graph depicting a significant decrease in the number of nodes and junction after treatment with necrostatin-1. Scale bar, 200 µm. *P values below 0.05 compared to PBMC. *n* = 3

Next, we determined whether the type of cell death affects the pro-angiogenic potential of PBMC secretome. Therefore, irradiated PBMCs were cultivated in the presence of zVAD, a pan-caspase inhibitor, or necrostatin-1, an inhibitor of necroptosis, and the angiogenic capacity of the resulting secretome was assessed in murine aortic ring sprouting assays (Fig. 6b, c) and tube formation assays with HUVECs (Fig. 6d, e). Irradiation-induced c-cas 3 and phosphorylation of MLKL were efficiently abrogated by zVAD and necrostatin-1, respectively (Fig. 6a), indicating that γ-irradiated PBMCs treated with zVAD and necrostatin-1 preferentially undergo apoptosis or necroptosis. In both assay systems, blood vessel sprouting was strongly induced by the secretome of irradiated PBMCs (Fig. 6b–e), as described above, and was comparably high with blocked caspase-dependent apoptosis (zVAD, Fig. 6b–e). Intriguingly, the pro-angiogenic capacity of the secretome was remarkably compromised when necroptosis was inhibited by necrostatin-1 (Fig. 6b–e). Freshly added zVAD and necrostatin-1 to the secretome of γ-irradiated PBMC during the assay had no effect on vessel sprouting (Supplementary Fig. 7a). Our data indicate that necroptosis represents an indispensable prerequisite for the pro-angiogenic action of secretomes derived from γ-irradiated PBMCs.

### Necroptotic cell death in γ-irradiated PBMCs is induced via paracrine activation of the TNFRSF1B

**Fig. 7:**
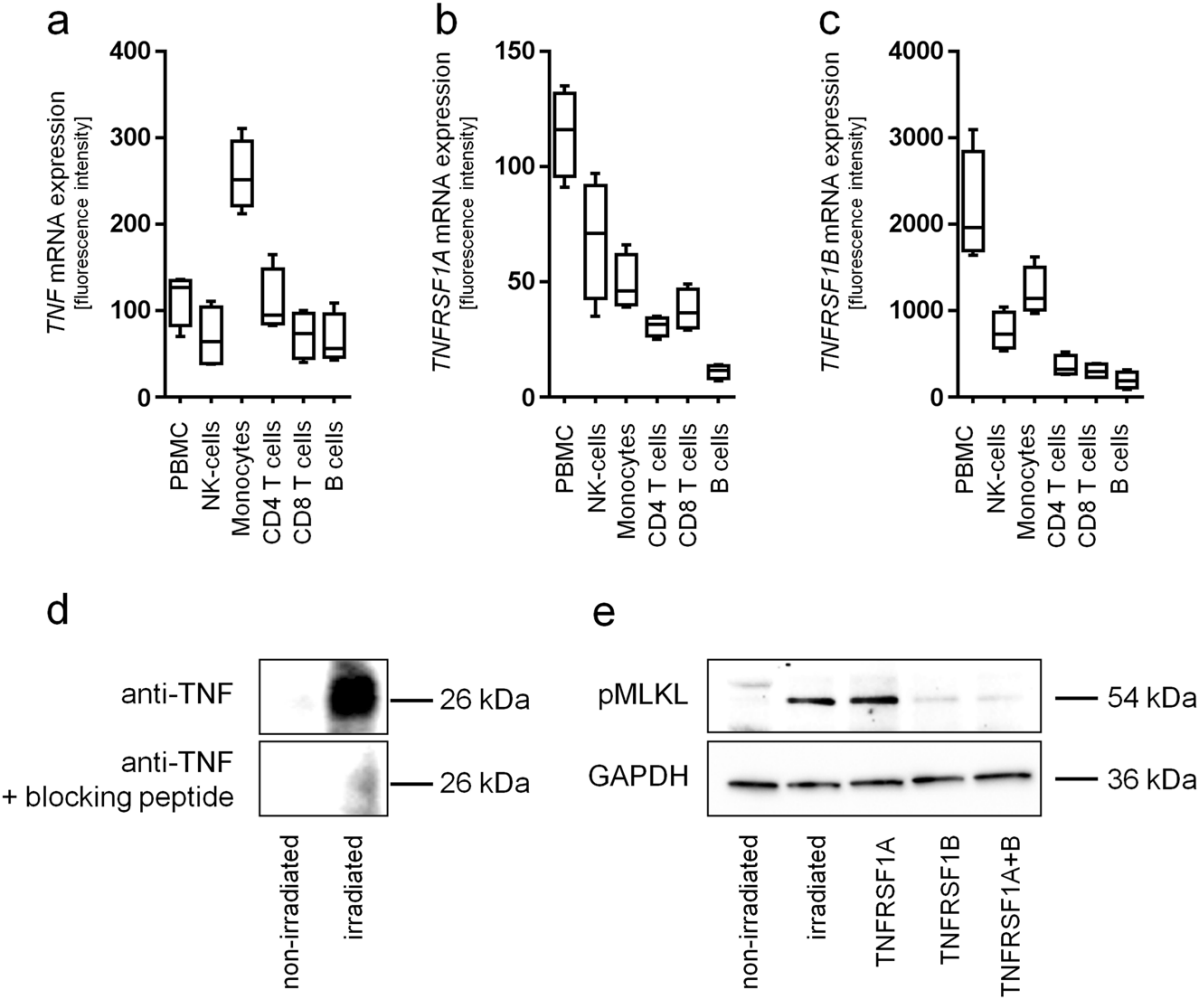
TNFRSF1B mediates radiation-induced necroptosis via a paracrine mechanism. Expressions of *TNF* (**a**), *TNFRSF1A* (**b**), and *TNFRSF1B* (**c**) assessed by microarray analysis are shown*. TNF* is expressed in PBMCs and in all subfractions. While expression of *TNFRSF1A* is low, *TNFRSF1B* is highly expressed in PBMCs and cell subsets. **d** Western blot analysis shows induction of membrane-bound TNF (mTNF) in PBMCs after γ-irradiation (upper panel). The specificity of the antibody was confirmed by pre-absorption of the antibody with recombinant TNF (lower blot). **e** Immunoblot analysis of phosphorylated MLKL in γ-irradiated PBMC treated with blocking antibodies against TNFRSF1A, TNFRSF1B, or a combination of both is shown. Blocking TNFRSF1B, but not TNFRSF1A, reduced necroptosis induction as shown by reduced MLKL phosphorylation. Diagrams show means of four independent experiments. Western blots show one representative experiment of 5

We next sought to elucidate the mechanism by which γ-irradiation induces necroptosis in PBMCs. Since previous studies identified the TNF-α pathway as one of the main drivers of necroptosis, and our bioinformatics analysis suggested an activation of the TNFRSF1B signalling pathway in response to irradiation, we analyzed the expression of *TNF* and its receptors (*TNFRSF1A* and *TNFRSF1B*). Low *TNF* expression was detectable in PBMCs and all subfractions, with highest mRNA levels in monocytes (Fig. 7a). Whereas *TNFRSF1A* showed little expression values in PBMCs and PBMC subsets (Fig. 7b), *TNFRSF1B* was strongly expressed in PBMCs and to a minor degree in all subsets (Fig. 7c). Interestingly, we neither detected soluble TNF-α nor LTA, a TNF homologous ligand of TNFRSF1A and TNFRSF1B, in the PBMC secretome (not shown). However, Western blot analysis showed a significant induction of membrane-bound TNF (mTNF) in PBMC after γ-irradiation (Fig. 7d). To further investigate the necroptosis signalling cascade, we specifically blocked both TNF receptors of γ-irradiated PBMCs with monoclonal blocking antibodies and assessed MLKL phosphorylation. As shown in Fig. 7e, induction of necroptosis was only effectively abolished by neutralizing antibody directed against TNFRSF1B, but not by TNFRSF1A. Our data thus indicate that γ-irradiation-induced necroptosis of PBMCs occurs via an mTNF-TNFRSF1B signalling cascade.

## Discussion

In the past, stem cell therapies had been praised as a promising therapeutic option for tissue regeneration of a variety of damaged organs^40–43^. Yet, most of the high expectations from in vitro and animal experiments were disappointed when stem cells employed in human clinical trials showed only minor tissue-regenerative potential^44^. We have reported previously that the release of regenerative factors is not an exclusive feature of stem cells, since secretomes derived from γ-irradiated PBMCs also displayed high tissue-regenerative activity in various experimental models. These regenerative effects were mainly attributed to the secretomes’ pro-angiogenic and cytoprotective properties^12,13,16,18^. These results raised the question whether all cell types are potentially capable of producing and releasing sufficient factors with tissue-regenerative properties after stress-induced cell death. Our analyses revealed pronounced differences in gene expression and released proteins between the respective cell types and total PBMCs in response to γ-irradiation. Importantly, certain cytokines were exclusively released when irradiated PBMCs were cultured together, but were not present in the secretomes derived from purified cell populations. In addition, using murine aortic rings for blood vessel sprouting assays, high pro-angiogenic activity was only detected in secretomes from total PBMCs. Both analyses suggest that a crosstalk of PBMC subpopulations is required for the release of angiogenesis-promoting factors. Therefore, our study argues against the initial hypothesis that the secretome of any stressed cell type exhibits tissue-regenerative characteristics and suggests that paracrine communication between different cell types is fundamental for the release of a unique composition of tissue-regenerating mediators. Thus, future clinical studies on damaged tissue will elucidate the full tissue-regenerative efficacy of the PBMC-derived secretome. Since toxicological studies and studies on the viral safety of an allogeneic secretome from γ-irradiated PBMC produced under Good Manufacturing Practice (GMP) conditions have already been successfully conducted, our study paves the way for a first clinical trial in the indication of diabetic foot ulcer^45,46^.

Here, we also investigated the impact of the type of cell death on the tissue-regenerative capacity of the secretome. Although γ-radiation is a known inducer for both apoptosis and necroptosis^20,47–51^, it is unknown whether γ-radiation-induced necroptosis has tissue-regenerative effects or further aggravates tissue damage. While Castle et al.^49^ showed that mice lacking RIP3, a critical molecule in the necroptosis pathway, were not rescued from acute radiation syndrome, a protective activity of necrostatin-1 administration in mice after lethal full body irradiation has been reported in several studies^47,52^. Although the underlying mechanisms are still not known, the aforementioned data by Huang et al.^47^ and Steinman et al.^52^ suggest that inhibition of necroptosis by necrostatin-1 is indeed favorable in a lethal setting, due to the prevention of massive cell death and organ destruction. To the best of our knowledge, our study is the first to describe that necroptosis of PBMCs exerts pro-angiogenetic effects, thereby potentially contributing to tissue regeneration in chronically damaged tissues. As shown by aortic ring assays, addition of necrostatin-1 to PBMCs before irradiation abolishes its pro-angiogenic activity, indicating that necroptosis is important for the release of factors involved in blood vessel formation. However, since PBMCs used in this study were ex vivo γ-irradiated and then applied to the tissue, we do not currently know whether similar processes are also present in stressed tissue in vivo, or in tissues under physiological conditions. Our study builds a basis for further studies, which would address these questions in more sophisticated experiments. Another interesting finding was the detection of low molecular forms of phosphorylated RIPK3 after γ-irradiation. Whether these forms are still active or only non-functional degradation products occurring during massive cell death after γ-irradiation remains to be determined.

Here we identified the TNF/TNFRSF1B signalling cascade as an inducer of necroptosis after γ-irradiation. In line with our observations, recent guidelines of the American College of Rheumatology instruct doctors to stop medication with anti-TNF therapy before surgery to avoid wound healing problems, highlighting the importance of TNF for proper wound healing, presumably due to its necroptosis-inducing action^53^. Interestingly, release of soluble TNF was not detectable in our secretomes. However, Western blot analysis revealed a significant increase in membrane-bound TNF. When analyzing TNF receptors, we found strong expression of TNFRSF1B. This is in line with previous studies, suggesting that mTNF preferentially signals through TNFRSF1B^54,55^. Currently, the mechanism of γ-radiation-induced necroptosis is still not fully understood. While activation of necroptosis by TNF requires additional inhibition of apoptosis (Supplementary Fig. 7b), γ-irradiation simultaneously induced necroptosis and apoptosis in PBMC^22,56,57^. Since high-dose ionizing radiation leads to DNA damage, activation of cytosolic DNA sensors could account for this phenomenon. Indeed, the cytosolic DNA sensor DNA-dependent activator of interferon regulatory factors has been shown to directly induce necroptosis via RIPK3 after virus infection^58^. Further studies are needed to investigate whether similar mechanisms also account for the induction of necroptosis in our experimental setting.

In conclusion, we could demonstrate that secretomes of PBMCs and PBMC subsets show different tissue-regenerative capacities, refuting the paradigm that any cell type is able to release paracrine factors with regenerative potential. Furthermore, we identified the TNF/TNFRSF1B signalling pathway as the mechanism underlying the γ-irradiation-induced release of pro-angiogenic factors. Based on these findings we believe that necroptosis, although seemingly paradox, is indeed an essential prerequisite for tissue regeneration and that forced induction of necroptosis might facilitate the development of novel therapeutic approaches in the near future.

## Supplementary information

Supplemental Figures

Supplemental table 1

Supplemental table 2

Supplemental table 3

Supplemental table 4
