# Supplementary material for: Tissue-regenerative potential of the secretome of γ-irradiated peripheral blood mononuclear cells is mediated via TNFRSF1B-induced necroptosis
Source: Cell Death Dis. 2019 Sep 30;10(10):729. doi: 10.1038/s41419-019-1974-6 (PMC6768878; doi:10.1038/s41419-019-1974-6)
Supplement: Supplementary file 1 — Supplemental Figures [file 41419_2019_1974_MOESM1_ESM.docx]

**Supplementary Figure 1**


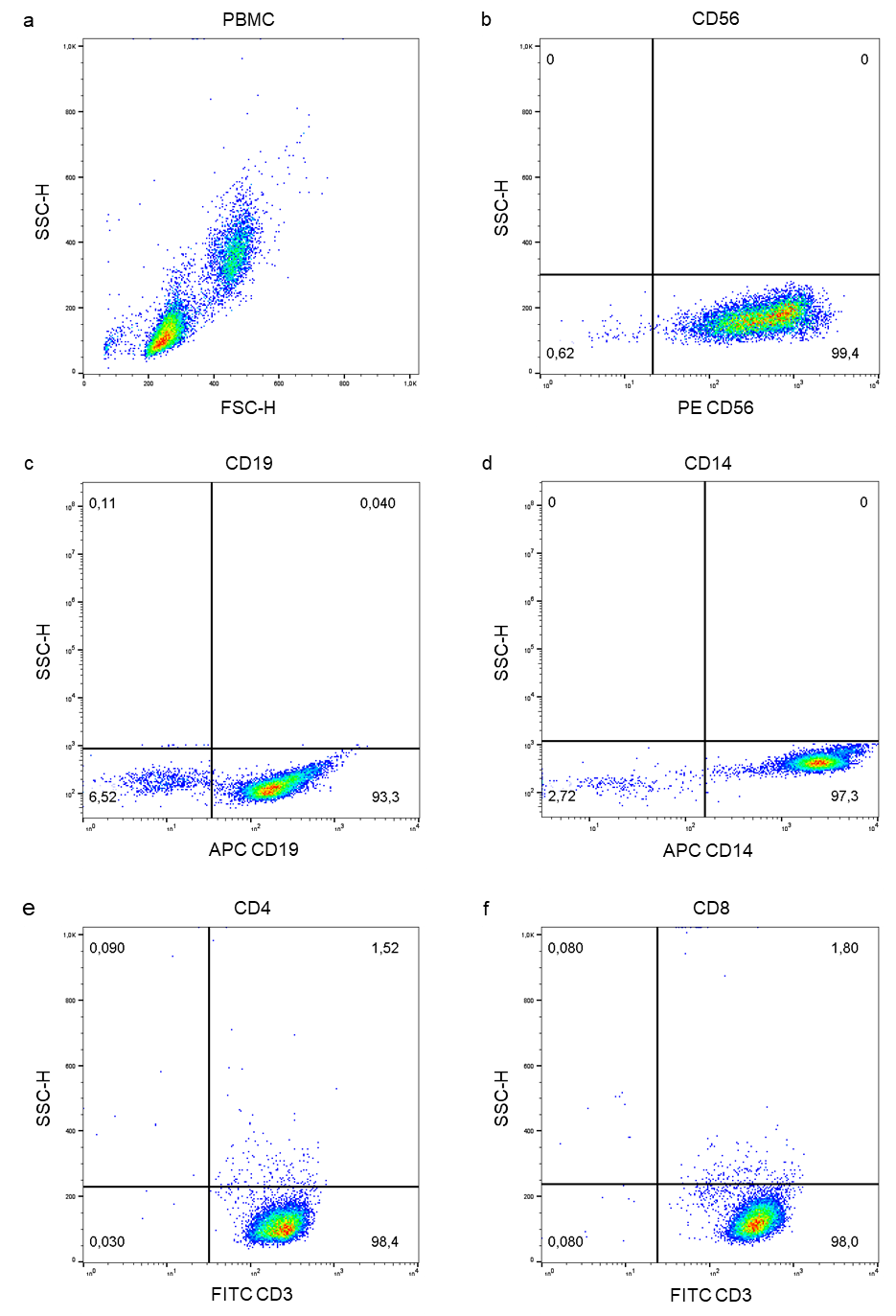


**Supplementary Figure 1. Purity of isolated PBMC subsets.** Polychromatic flow cytometric analysis for purified PBMC subsets. a) Forward-sidescatter dot plot of PBMCs. b) Purified natural killer cells expressing CD56 (99.4% cell purity). c) Purified B-cells expressing CD19 (93.3% cell purity). d) Purified monocytes expressing CD14 (97.3 cell purity). e) Purified CD4 T-cells expressing CD3 (95.4%). f) Purified CD8 T-cells expressing CD3 (98% cell purity). One experiment out of three is shown.

**Supplementary Figure 2**

**
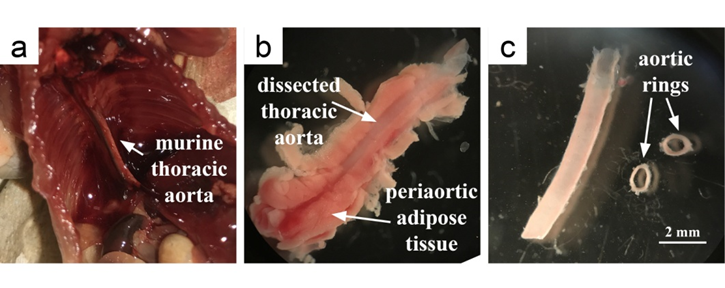
**

**Supplementary Figure 2. Preparation of aortic rings.** a) Mice were sacrificed via cervical dislocation, the rib cage was opened, heart and lungs were removed. c) Thoracic aorta including periaortic adipose tissue was carefully dissected from vertebrae. c) Adipose tissue was surgically removed and the aorta was cut in 1 mm-thick rings.

**Supplementary Figure 3**

**
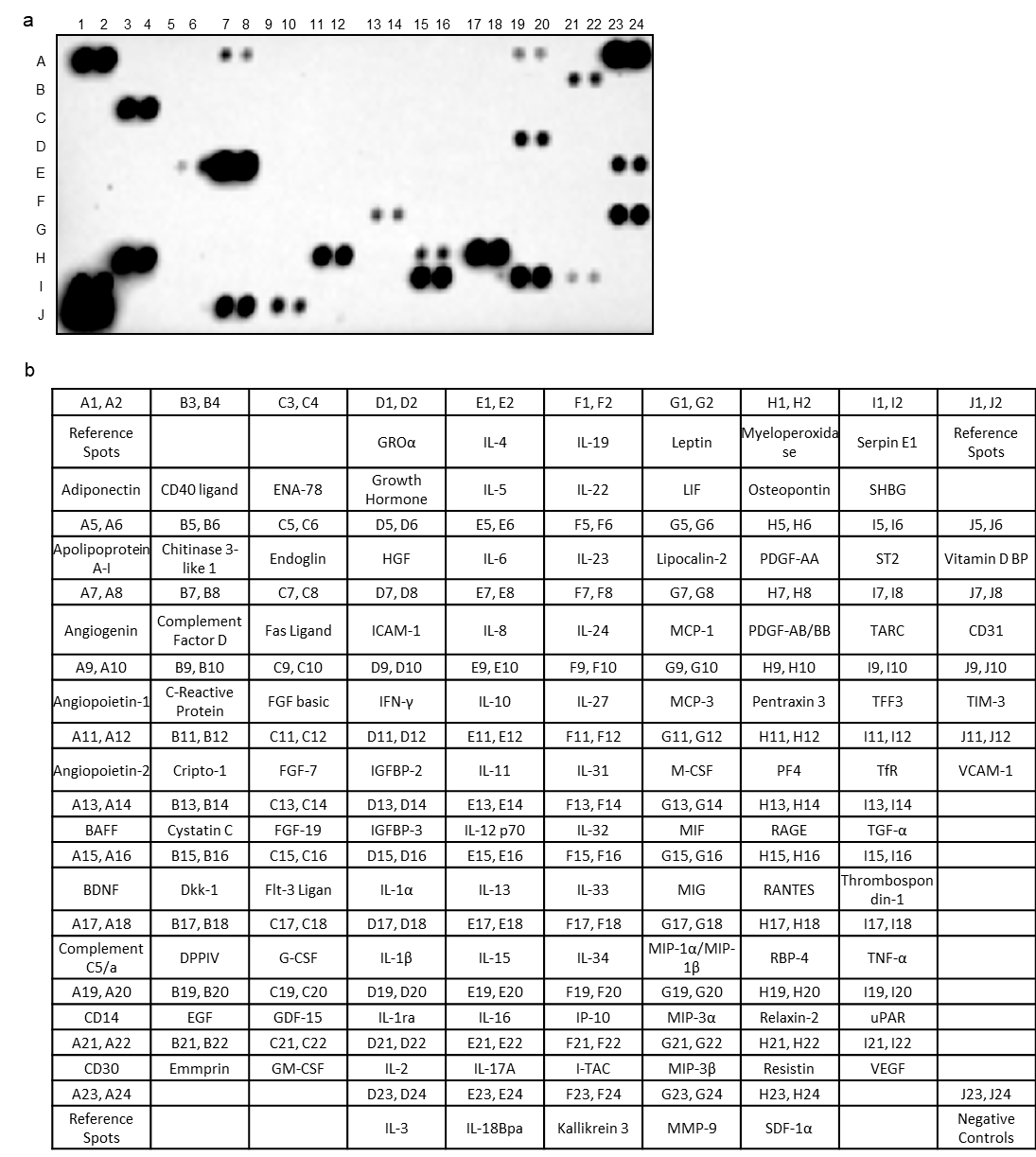
**

**Supplementary Figure 3. Cytokine protein array of γ-irradiated PBMCs.** a) Membrane arrays detecting 102 cytokines and cytokine-related proteins were incubated with supernatant from 25x10^6^ γ-irradiated PBMCs 24 hours after incubation. b) Legend for spotted antibodies.

**Supplementary Figure 4**

**
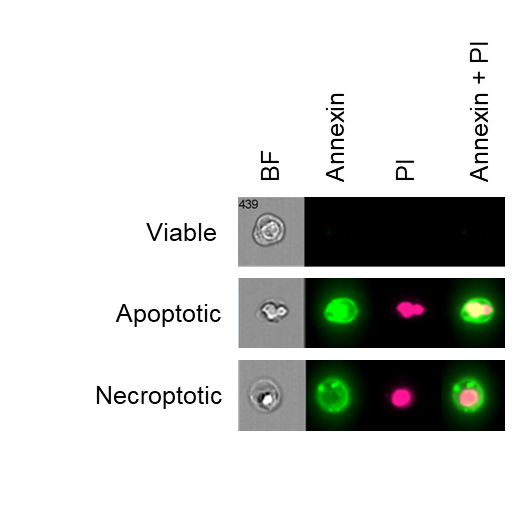
**

**Supplementary Figure 4.** Representative image stream micrographs of viable, apoptotic, and necroptotic cells. Viable cells were morphologically characterised by their intact cell shape in the bright field (BF) channel and by the lack of annexin and propidium iodide (PI). Apoptotic cells showed decreased cell volume, exposed Annexin on the surface, and displayed nuclear fragmentation (PI positive). Cells undergoing necroptosis were enlarged, indicating cytoplasmic swelling and the nucleus remained non-fragmented.

**Supplementary Figure 5**

**
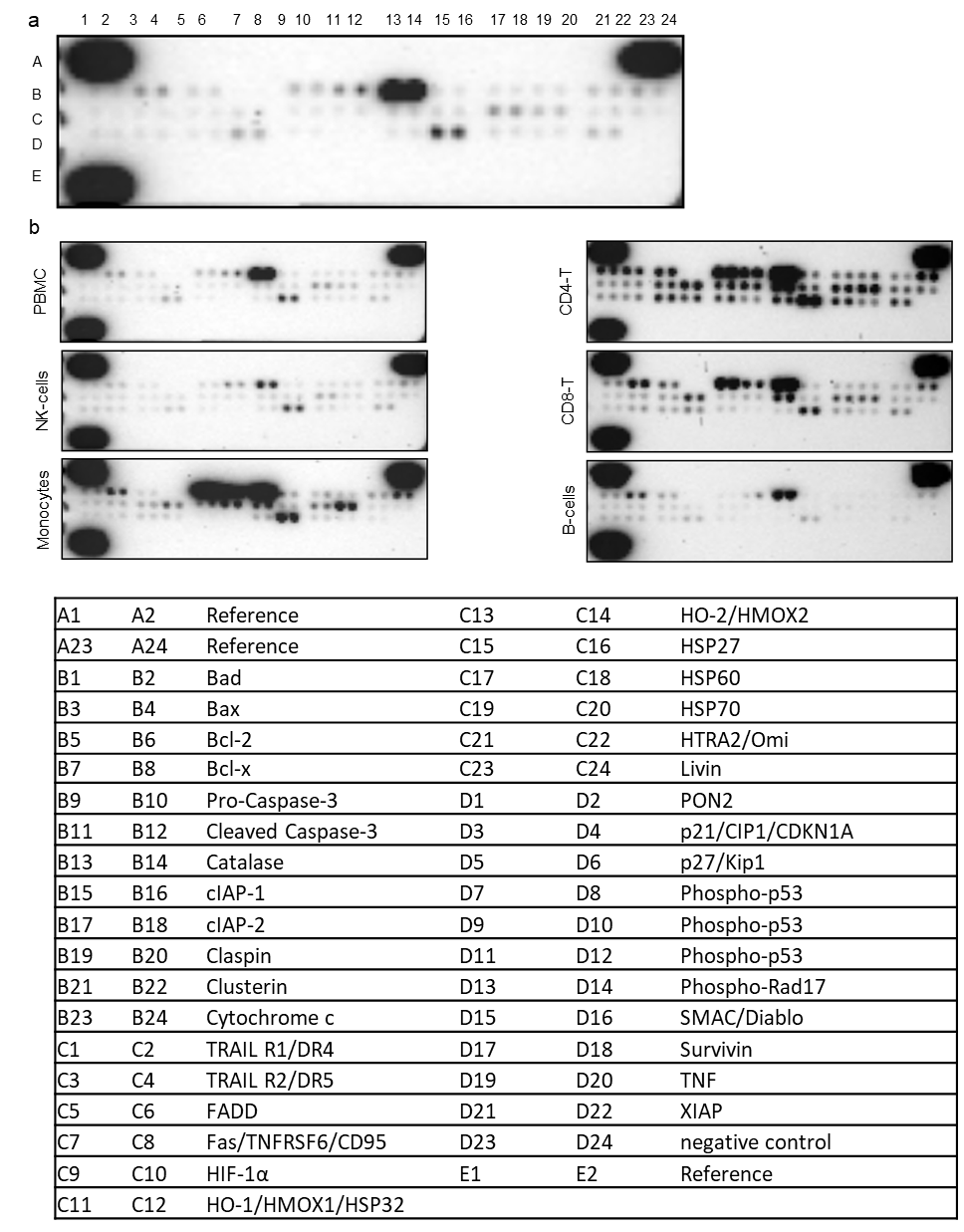
**

**Supplementary Figure 5. Array of apoptosis-related proteins secreted by γ-irradiated PBMCs.** a) Membrane arrays detecting 35 apoptosis-related proteins were incubated with cell lysates from 25x10^6^ γ-irradiated PBMCs 24 hours after exposure. b) Plots of PBMCs, NK-cells, monocytes, CD4 T-cells, CD8 T-cells, and B-cells are shown. Table lists the proteins analysed.

**Supplementary Figure 6**

**
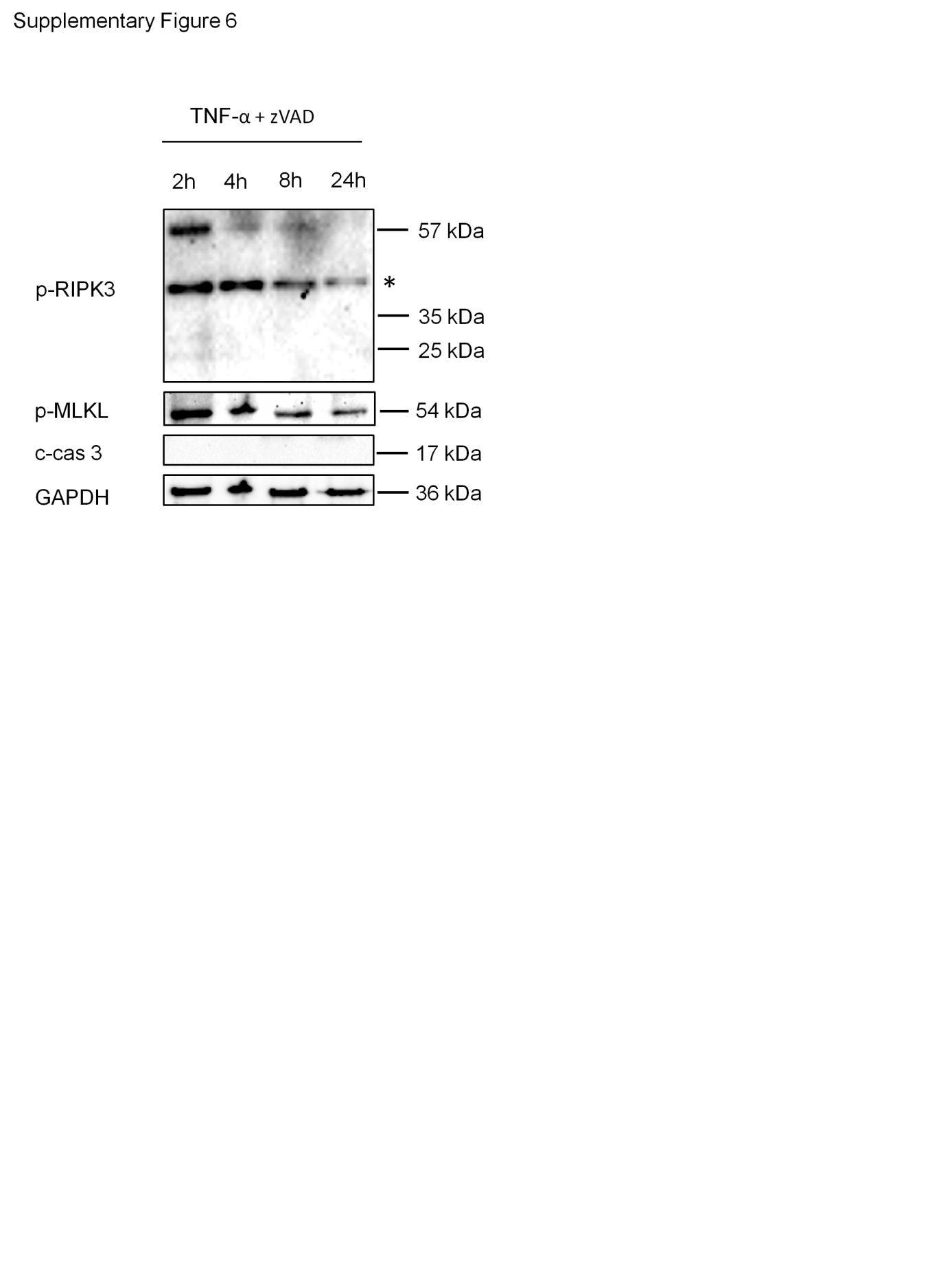
**

**Supplementary Figure 6. TNFα and zVAD induce necroptosis in PBMCs.** PBMCs were stimulated with a combination of TNFα and zVAD and cultivated for up to 24 hours. Co-incubation of PBMCs with TNFα and zVAD abrogated caspase-3 cleavage and lead to phosphorylation of RIPK3 and MLKL. Stimulation with TNFα and zVAD did not induce RIPK3 cleavage products with molecular weights of 35 and 25 kDa as observed after γ-irradiation. The position of an unidentified, most likely unspecific, band on the p-RIPK3 Western blot is indicated by an asterisk. n=3.

**Supplementary Figure 7**


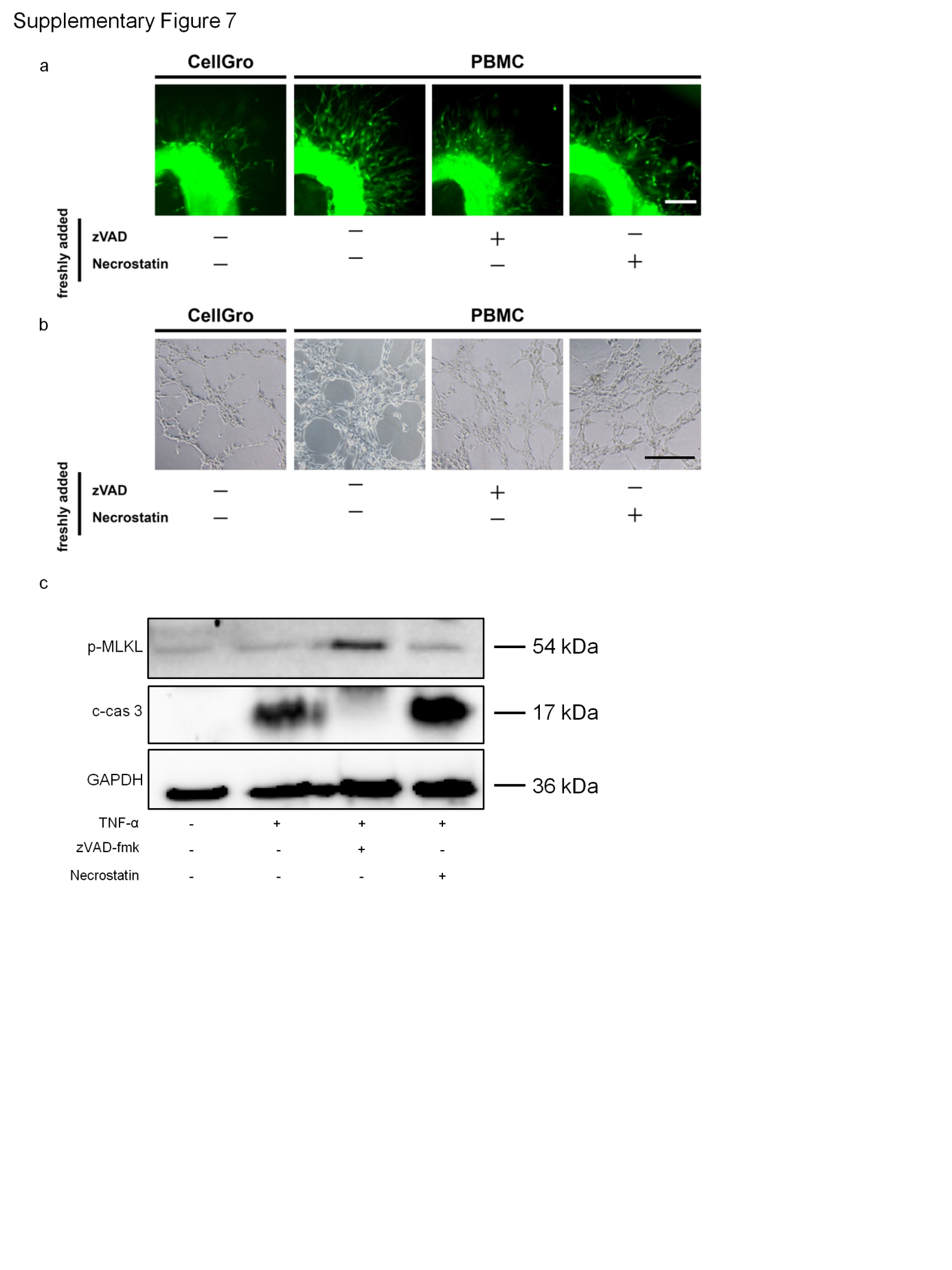


**Supplementary Figure 7. TNFα induces apoptosis and necroptosis in PBMCs which were inhibited by zVAD and necrostatin, respectively.** a) Aortic rings were incubated with the secretome of γ-irradiated PBMCs together with freshly added zVAD, necrostatin or a combination of both for 3 days. Thereafter calcein (green dye) was added to label viable cells. Neither zVAD nor necrostatin significantly inhibited blood vessel sprouting in the aortic ring assay. Scale bar, 200 µm. n=3. b) Endothelial cells were incubated with the secretome of γ-irradiated PBMCs together with freshly added zVAD and necrostatin for 3 hours after overnight starvation. Cell Gro medium was used as negative control. The tube formation was diminished in the medium control, yet the fresh addition of zVAD and necrostatin had no effect on endothelial outgrowth compared to the PBMC secretome. Scale bar, 200 µm. c) PBMCs were stimulated with combinations of TNFα, zVAD and necrostatin and were cultivated for 24 hours. Stimulation with TNFα resulted in cleavage of caspase-3 (c-cas 3). Co-incubation of PBMCs with TNFα and zVAD abrogated caspase-3 cleavage and lead to phosphorylation MLKL while co incubation with necrostatin favored caspase-3 cleavage. n=3
