# Supplementary material for: Tissue-regenerative potential of the secretome of γ-irradiated peripheral blood mononuclear cells is mediated via TNFRSF1B-induced necroptosis
Source: Cell Death Dis. 2019 Sep 30;10(10):729. doi: 10.1038/s41419-019-1974-6 (PMC6768878; doi:10.1038/s41419-019-1974-6)
Supplement: Supplementary file 2 — Supplemental table 1 [file 41419_2019_1974_MOESM2_ESM.docx]

| **Supplementary Table 1. Mean expression levels of differentially expressed genes between PBMC and PBMC subsets after 60 Gy γ-irradiation.** | | | | | | |
| --- | --- | --- | --- | --- | --- | --- |
| **GeneSymbol** | **PBMC** | **NK-cell** | **Monocyte** | **CD4 T-cell** | **CD8 T-cell** | **B-cell** |
| PPBP | 4.72 | 2.27 | 2.09 | 0.11 | 0.02 | 0.22 |
| FCAR | 3.95 | 2.59 | 3.14 | 0.32 | -0.58 | -0.79 |
| CD14 | 3.95 | 2.65 | 1.38 | -0.09 | -0.98 | -1.12 |
| TREM1 | 3.60 | 1.10 | 2.96 | -0.69 | -0.82 | -0.78 |
| RNASE6 | 3.57 | 0.79 | 0.24 | -1.42 | -1.55 | 0.49 |
| HILPDA | 3.57 | 1.34 | 6.26 | 0.12 | -0.09 | 0.17 |
| VCAN | 3.52 | 1.86 | 3.39 | -0.79 | -0.01 | -0.55 |
| FPR3 | 3.38 | 2.63 | 1.21 | 0.56 | 0.69 | 0.07 |
| FPR1 | 3.28 | 0.77 | 0.97 | -0.73 | -0.65 | -0.73 |
| THBS1 | 3.28 | 2.80 | 1.33 | 0.66 | -0.87 | -1.60 |
| ANPEP | 3.27 | 1.69 | 2.46 | -0.24 | -0.53 | -1.10 |
| LRP1 | 3.17 | 0.55 | 1.99 | -0.60 | -0.44 | -0.85 |
| SULF2 | 3.15 | -0.32 | 2.54 | -0.95 | -0.57 | -1.01 |
| S100A12 | 3.05 | 1.91 | 3.36 | -0.51 | -1.34 | -1.51 |
| ANG\|RNASE4 | 3.02 | 0.45 | 0.09 | 0.00 | -0.14 | -0.12 |
| HBA1 | 2.97 | -0.16 | 0.19 | -0.11 | 0.14 | -0.06 |
| CXCL16 | 2.94 | 0.67 | 0.42 | -0.07 | -1.23 | -1.57 |
| EPB41L3 | 2.90 | 1.68 | 2.98 | 0.15 | -0.07 | -0.40 |
| RNASE1 | 2.89 | 1.15 | 0.56 | 0.56 | 0.00 | -0.24 |
| FCGR2B | 2.85 | -0.18 | -0.20 | -0.21 | -0.22 | 1.53 |
| CD300E | 2.85 | 1.94 | 2.46 | -0.49 | -0.34 | -0.68 |
| HK2 | 2.81 | 1.41 | 3.80 | -0.13 | -0.56 | -0.40 |
| CLEC7A | 2.80 | 0.39 | 3.07 | -0.86 | -0.74 | -0.93 |
| ZNF385A | 2.80 | 0.27 | 1.15 | -0.64 | -1.26 | -1.25 |
| DNER | 2.75 | 1.23 | 0.31 | 0.08 | -0.01 | 0.00 |
| DEFA1B | 2.72 | 0.34 | -0.09 | 0.09 | 0.29 | 0.27 |
| PLA2G7 | 2.70 | 2.83 | 1.05 | 1.44 | -0.49 | -1.15 |
| SEMA6B | 2.63 | 2.03 | 2.94 | 0.15 | 0.21 | -0.07 |
| VEGFA | 2.56 | 1.05 | 3.59 | -0.04 | -0.17 | -0.13 |
| CSF3R | 2.55 | 0.29 | 2.90 | -0.46 | -0.27 | -0.32 |
| VMO1 | 2.54 | 0.75 | 0.26 | 0.25 | 0.12 | -0.27 |
| CD300LB | 2.52 | -0.23 | 2.74 | -0.69 | -0.64 | -0.83 |
| BST1 | 2.52 | 0.47 | 0.72 | -0.44 | -0.30 | -0.38 |
| CCL22 | 2.50 | 3.07 | -0.31 | 2.57 | 1.85 | 3.04 |
| IRAK3 | 2.46 | 1.72 | 2.57 | -0.36 | -0.81 | -0.80 |
| SDC4 | 2.41 | 1.42 | 1.97 | 0.25 | -0.39 | -0.42 |
| LRRC25 | 2.38 | 0.38 | 0.00 | -0.74 | -0.91 | -0.86 |
| TNFRSF10D | 2.37 | 1.15 | 1.66 | 1.02 | -0.21 | -0.45 |
| PLXDC2 | 2.36 | 1.45 | 2.10 | -0.92 | -0.30 | -0.84 |
| NFAM1 | 2.35 | 0.17 | 1.18 | -0.45 | -0.33 | -0.52 |
| SIRPB2 | 2.35 | -0.09 | 1.10 | -0.24 | -0.20 | -0.20 |
| CSF1R | 2.33 | -0.27 | 0.08 | -1.11 | -1.14 | -1.60 |
| CCL24 | 2.33 | 1.17 | 0.11 | 0.21 | 0.30 | -0.15 |
| PLAUR | 2.32 | 1.92 | 2.36 | 0.17 | -0.64 | -1.14 |
| MIR3143 | 2.30 | -0.28 | 3.46 | 0.37 | 0.28 | -0.55 |
| CD86 | 2.29 | 0.35 | 0.21 | -0.65 | -0.46 | 0.27 |
| CPNE2 | 2.29 | 0.68 | 0.05 | -0.50 | -0.27 | -0.64 |
| AQP9 | 2.29 | 2.58 | 3.14 | 0.10 | -0.40 | -0.28 |
| SLC37A2 | 2.28 | 0.25 | 1.84 | -0.31 | -0.44 | -0.15 |
| CCR4 | 2.27 | -0.51 | -0.58 | 3.92 | 1.58 | -0.48 |
| ADM | 2.26 | 1.02 | 4.33 | -0.04 | 0.04 | -0.18 |
| ENG | 2.25 | 0.98 | 0.11 | -0.62 | -0.68 | -1.27 |
| CLEC4E | 2.24 | 1.77 | 1.00 | 0.10 | -0.34 | -0.45 |
| CDA | 2.23 | -0.50 | 0.48 | -1.11 | -1.10 | -1.01 |
| SLC11A1 | 2.22 | 1.01 | 3.21 | -0.70 | -0.75 | -0.53 |
| S100A8 | 2.22 | 1.57 | 2.74 | -0.60 | -1.05 | -1.18 |
| CLC | 2.22 | 0.02 | 0.05 | -0.11 | 0.60 | 0.90 |
| NCF2 | 2.21 | 1.17 | 2.50 | -0.34 | -1.07 | -1.42 |
| SIGLEC7 | 2.20 | 0.24 | 0.03 | -0.62 | -0.59 | -0.80 |
| F11R | 2.19 | 0.57 | -0.84 | -0.05 | -0.36 | -0.43 |
| NLRP3 | 2.15 | 0.81 | 2.18 | -0.35 | -0.36 | -0.63 |
| HBA2 | 2.15 | -0.06 | 0.45 | -0.04 | 0.02 | -0.13 |
| SIRPA | 2.12 | 1.49 | 1.11 | 0.01 | -0.89 | -1.05 |
| TMEM51 | 2.12 | 0.69 | 0.90 | 0.21 | -0.11 | -0.18 |
| SIRPB1 | 2.12 | -0.40 | 0.34 | -0.62 | -0.53 | -0.65 |
| GPR162 | 2.11 | 0.68 | 0.33 | -0.03 | -0.08 | -0.22 |
| IFI6 | 2.10 | -0.72 | 0.27 | 0.04 | -1.09 | -1.61 |
| SLC16A3 | 2.10 | 0.29 | 1.89 | -0.70 | -0.67 | -1.04 |
| BNIP3 | 2.09 | -0.12 | 1.88 | 0.07 | -0.25 | -0.76 |
| CDCP1 | 2.08 | 0.60 | 0.33 | -0.02 | -0.11 | -0.13 |
| CDKN1A | 2.06 | 0.59 | 0.97 | 0.51 | -0.10 | -0.08 |
| NRGN | 2.05 | -0.18 | 0.77 | -0.38 | -0.27 | -0.48 |
| LYZ | 2.04 | 1.10 | 0.68 | 0.08 | -0.41 | -1.58 |
| PTGFRN | 2.04 | 0.25 | -0.04 | 0.10 | 0.29 | -0.02 |
| P4HA1 | 2.04 | 0.62 | 2.27 | -0.20 | -0.18 | 0.00 |
| SCD | 2.03 | 0.61 | 0.82 | 0.13 | -0.56 | -0.52 |
| PLXNB2 | 2.03 | 0.10 | 2.22 | -0.57 | -0.04 | -0.28 |
| SIGLEC9 | 2.02 | 0.53 | 1.13 | -0.81 | -0.88 | -0.92 |
| ADGRE3 | 2.01 | 0.01 | 0.27 | -0.26 | -0.06 | -0.02 |
| CCR1 | 2.00 | 1.75 | 1.14 | -0.14 | -0.49 | -0.62 |
| EMILIN2 | 2.00 | 1.07 | 1.37 | -0.48 | -0.74 | -0.45 |
| GPNMB | 2.00 | 1.86 | 0.84 | 0.60 | 0.07 | -0.16 |
| GPX1 | 1.99 | 0.14 | 1.17 | -0.45 | -0.82 | -0.80 |
| MS4A1 | 1.99 | 0.31 | -0.67 | -0.36 | 0.18 | 6.11 |
| IER3 | 1.99 | 1.37 | 1.93 | -0.11 | -0.23 | -0.54 |
| C15orf48 | 1.99 | 1.98 | 3.42 | 0.48 | 0.01 | -0.02 |
| FCN1 | 1.99 | -0.55 | 2.17 | -1.54 | -1.69 | -1.75 |
| CYP2S1 | 1.97 | 0.27 | -0.07 | -0.14 | -0.13 | -0.31 |
| PLPPR2 | 1.97 | 0.68 | 0.90 | -0.34 | -0.16 | -0.58 |
| ALDOC | 1.96 | -0.29 | 1.31 | -0.28 | -0.52 | -0.71 |
| RAB3D | 1.96 | -0.01 | -0.11 | -0.68 | -1.00 | -0.80 |
| C5AR2 | 1.94 | 0.24 | 1.14 | 0.01 | -0.39 | -0.40 |
| GAA | 1.94 | -0.34 | 0.25 | -0.53 | -0.35 | -0.31 |
| TIMP2\|CEP295NL | 1.92 | 0.01 | 0.55 | -0.32 | -0.68 | -0.95 |
| VNN2 | 1.92 | 1.30 | -0.09 | -0.81 | -0.74 | -0.16 |
| PYGL | 1.92 | 0.58 | 0.73 | -0.19 | -0.10 | -0.27 |
| SLC44A1 | 1.92 | 0.76 | -0.06 | -0.16 | -0.41 | -0.09 |
| IGLC2\|IGLJ3 | 1.92 | -0.89 | -2.02 | -1.34 | -0.16 | 5.21 |
| PILRA | 1.92 | 0.14 | 1.05 | -0.63 | -0.99 | -1.12 |
| GNA15 | 1.91 | 1.04 | 2.52 | 0.11 | -0.67 | -1.06 |
| HIST1H3H | 1.91 | -0.37 | 3.24 | 0.04 | 0.20 | -0.56 |
| SLC43A2 | 1.89 | 0.41 | 1.68 | -0.52 | -0.92 | 0.27 |
| GRN | 1.89 | -0.24 | 0.51 | -1.13 | -1.88 | -0.99 |
| GLUL | 1.87 | 0.84 | 3.36 | -0.27 | -0.53 | -0.77 |
| RASGRP4 | 1.87 | -0.41 | 0.26 | -0.84 | -1.03 | -0.83 |
| ADGRE2 | 1.87 | 0.18 | 1.20 | -0.56 | -0.47 | -0.73 |
| ALOX5 | 1.87 | -1.39 | 1.04 | -1.33 | -1.03 | 2.57 |
| CXCL5 | 1.86 | 5.06 | 1.73 | 1.88 | 0.43 | 0.04 |
| GPI | 1.86 | -0.58 | -0.51 | -0.56 | -0.33 | -0.75 |
| CD68 | 1.85 | 0.50 | 1.36 | -0.40 | -2.37 | -2.62 |
| IGHA1 | 1.85 | -0.51 | -0.58 | -0.67 | 0.01 | 5.16 |
| CSF2RA | 1.84 | 1.00 | 1.74 | 0.09 | -0.22 | -0.54 |
| CSTA | 1.84 | 0.61 | 1.70 | -0.24 | -0.46 | -0.60 |
| CFD | 1.83 | -0.88 | 0.76 | -0.94 | -0.89 | -0.88 |
| FAM129B | 1.83 | 1.05 | 2.26 | -0.27 | -0.16 | -0.63 |
| GNS | 1.83 | 0.68 | 0.15 | -0.40 | -0.93 | -1.15 |
| ST6GALNAC2 | 1.82 | 0.06 | 0.25 | 0.35 | -0.48 | -0.50 |
| TUBB1 | 1.81 | 0.12 | 0.87 | -0.08 | -0.22 | -0.20 |
| VNN1 | 1.81 | 1.69 | 0.62 | -0.01 | -0.21 | -0.19 |
| RRAS | 1.79 | -0.47 | 0.21 | -0.72 | -1.40 | -1.56 |
| HPSE | 1.79 | 1.32 | 0.97 | -0.27 | -0.47 | -0.53 |
| IFI30 | 1.78 | 0.22 | 0.58 | -0.81 | -1.96 | -1.45 |
| NAGA | 1.76 | 0.18 | -0.21 | -0.36 | -0.47 | -0.46 |
| SLC43A3\|PRG2 | 1.75 | 1.12 | 1.71 | -0.03 | -0.61 | -1.00 |
| SLC16A10 | 1.74 | 1.53 | 0.95 | 0.58 | -0.04 | -0.53 |
| TLR2 | 1.73 | 1.99 | 2.84 | -0.12 | -0.39 | -0.28 |
| BNIP3L | 1.72 | 0.75 | 2.08 | -0.06 | -0.43 | -0.16 |
| OGFRL1 | 1.71 | 0.46 | 0.23 | -0.74 | -0.73 | -0.05 |
| APLP2 | 1.69 | 0.36 | 0.65 | -0.84 | -1.13 | 0.35 |
| METTL7A | 1.68 | -0.18 | -1.42 | -0.97 | -1.28 | 0.81 |
| LOC102724994\|LOC102724995\|LOC101929599\|LOC105369229\|LOC101929812 | 1.67 | 0.69 | 1.20 | -1.32 | -0.82 | -0.77 |
| LOC441081 | 1.67 | 0.69 | 1.20 | -1.32 | -0.82 | -0.77 |
| LOC728093\|LOC643784 | 1.67 | 0.69 | 1.20 | -1.32 | -0.82 | -0.77 |
| C5AR1 | 1.67 | 0.07 | 0.99 | -1.10 | -2.02 | -2.33 |
| FGL2 | 1.62 | 0.45 | 1.37 | -1.08 | -0.64 | -0.62 |
| CSF2RB | 1.59 | 0.23 | 0.00 | -0.40 | -0.48 | 0.02 |
| IGLC1\|IGLV4-3\|IGLV3-25\|IGLC3 | 1.58 | -0.50 | -1.09 | -0.73 | 0.05 | 4.72 |
| ORAI3 | 1.58 | -0.18 | -0.50 | -0.73 | -0.69 | -0.61 |
| ABCC3 | 1.57 | -0.37 | 0.76 | -0.45 | -0.10 | -0.17 |
| CEBPD | 1.57 | -0.01 | 0.68 | -1.33 | -1.14 | -1.42 |
| CD101 | 1.56 | -0.38 | -0.44 | -0.22 | 0.57 | -0.76 |
| NCF4 | 1.55 | -0.79 | 0.16 | -0.08 | -1.02 | 1.39 |
| SIGLEC14 | 1.54 | 0.05 | 1.20 | -1.14 | -1.26 | -0.51 |
| HIST1H3A | 1.54 | 0.30 | 3.69 | 0.44 | 0.91 | -0.50 |
| CD4 | 1.53 | -0.77 | -0.27 | 0.79 | -0.75 | -0.81 |
| IL10RB | 1.53 | 0.35 | 0.77 | -0.14 | -0.97 | -0.48 |
| AIF1 | 1.52 | -0.98 | 0.38 | -1.52 | -2.02 | -2.44 |
| RXRA | 1.51 | 0.09 | 1.84 | -0.38 | -0.38 | -0.64 |
| EREG | 1.50 | 1.77 | 1.88 | -0.22 | -0.41 | -0.93 |
| HEXB | 1.50 | 0.83 | 0.82 | -0.27 | -0.79 | -0.95 |
| NDRG1 | 1.50 | 0.04 | 1.13 | -0.19 | -0.25 | -0.66 |
| S100A9 | 1.49 | 0.84 | 2.66 | -1.24 | -1.65 | -1.91 |
| CEBPA | 1.48 | -0.29 | 0.40 | -0.93 | -0.39 | -0.77 |
| BLVRB | 1.48 | -0.38 | -0.03 | -0.67 | -0.98 | -0.91 |
| RTN3 | 1.48 | 0.16 | -0.25 | -0.26 | -0.67 | -0.67 |
| CD300C | 1.47 | 0.03 | 2.51 | -0.77 | -0.57 | -0.79 |
| RAB31 | 1.47 | 0.56 | 0.34 | -1.66 | -1.25 | -0.07 |
| ABCA1 | 1.47 | 0.45 | 3.79 | 0.09 | -0.26 | 0.84 |
| MPP1 | 1.46 | 0.84 | 1.77 | 0.09 | -0.45 | -0.83 |
| IGSF6 | 1.45 | 1.78 | 1.15 | -0.28 | -0.82 | -0.89 |
| CD93 | 1.45 | 0.43 | 1.24 | -0.81 | -0.94 | -0.89 |
| CHST15 | 1.44 | -0.72 | 0.92 | -1.51 | -1.53 | 0.04 |
| CECR1 | 1.44 | -0.51 | -0.25 | -0.25 | -0.56 | -0.33 |
| DIRC2 | 1.43 | 0.07 | 1.31 | -0.44 | -0.77 | 0.04 |
| MPEG1 | 1.43 | -0.29 | -0.08 | -1.48 | -2.26 | 1.00 |
| PRDM8 | 1.42 | 0.66 | -0.63 | -0.14 | -0.08 | 0.20 |
| LTA4H | 1.42 | 0.27 | -0.03 | -0.35 | -1.00 | 0.25 |
| IGKV1-39\|IGKV1D-39 | 1.42 | -0.52 | -0.75 | -0.82 | -0.50 | 4.97 |
| TYROBP | 1.41 | 0.31 | 0.13 | -0.95 | -2.64 | -3.64 |
| PEA15 | 1.41 | 0.32 | 0.49 | -0.52 | -1.31 | -0.53 |
| CTSZ | 1.40 | -0.04 | 0.13 | -0.79 | -1.03 | 0.65 |
| EEPD1 | 1.40 | 0.63 | 1.83 | 0.30 | -0.27 | -0.62 |
| IGKC\|IGKJ5 | 1.39 | -0.63 | -1.38 | -1.22 | -0.30 | 5.19 |
| SERPINB2\|SERPINB10 | 1.39 | 4.08 | 2.77 | 0.58 | 0.08 | -0.05 |
| CTSB | 1.38 | 0.69 | 0.70 | -0.22 | -1.39 | -1.64 |
| HIST1H2AB | 1.38 | -0.48 | 3.84 | -0.16 | -0.11 | -0.89 |
| RNF130 | 1.37 | 0.48 | 0.51 | -0.17 | -0.93 | -1.04 |
| DMXL2 | 1.37 | 1.73 | 3.85 | 0.02 | 0.15 | 0.02 |
| DDIT4 | 1.37 | 0.22 | 2.75 | -0.56 | -0.51 | -1.65 |
| TIMP1 | 1.36 | 0.10 | 0.74 | -0.46 | -1.11 | -1.78 |
| FBP1 | 1.34 | -0.10 | -0.10 | -0.81 | -0.78 | -0.79 |
| MEGF9 | 1.32 | 0.71 | -0.19 | -0.48 | -0.78 | -0.63 |
| C3AR1 | 1.32 | 1.13 | 0.45 | -0.16 | -0.50 | -0.70 |
| QSOX1 | 1.31 | 0.70 | 0.58 | -0.41 | -0.61 | -0.71 |
| HIST2H4B\|HIST2H4A | 1.30 | -0.37 | 0.31 | 0.24 | -0.12 | -1.02 |
| IFNGR2 | 1.29 | -0.06 | 0.75 | -0.69 | -1.32 | -0.14 |
| MIR21\|VMP1 | 1.28 | 1.86 | 2.35 | -0.36 | -0.76 | -1.03 |
| C19orf38 | 1.28 | -0.69 | 0.47 | -1.87 | -2.11 | -2.01 |
| FCER1G | 1.27 | 0.70 | 0.64 | -1.22 | -2.57 | -3.35 |
| IGH\|IGHA2 | 1.27 | -0.62 | 0.16 | -0.86 | 0.14 | 4.31 |
| HCK | 1.26 | 0.32 | 1.13 | -1.44 | -1.49 | -0.48 |
| HIST1H2BB | 1.26 | -0.13 | 4.16 | 0.15 | 0.28 | -0.40 |
| IGKV1-5\|IGKV2D-28\|IGKV2-28\|IGKC | 1.26 | -0.36 | -0.31 | -0.53 | -0.21 | 4.34 |
| AMICA1 | 1.26 | -0.72 | -0.10 | 0.46 | 0.49 | -1.27 |
| ARRB2 | 1.26 | 0.17 | -0.32 | -0.54 | -0.21 | -1.46 |
| BCKDK | 1.26 | -0.03 | 1.27 | -0.71 | -0.90 | -0.71 |
| PSAP | 1.24 | -0.23 | 0.38 | -0.75 | -2.03 | -1.60 |
| NPC2\|MIR4709 | 1.23 | 0.45 | 0.15 | -0.52 | -1.79 | -1.48 |
| RNASE2 | 1.22 | -0.24 | 1.65 | -0.87 | -1.09 | -0.97 |
| PRKACA | 1.20 | 0.24 | -0.60 | -0.87 | -0.89 | -1.04 |
| LINC01272 | 1.20 | 0.00 | 0.84 | -0.54 | -0.98 | -1.13 |
| LAPTM4A | 1.20 | -0.14 | -0.34 | -0.33 | -1.36 | -0.91 |
| S100A11 | 1.19 | 0.15 | 0.82 | -0.11 | -0.81 | -1.51 |
| PKM | 1.19 | 0.16 | 0.87 | -0.57 | -0.69 | -0.83 |
| CHTF8 | 1.18 | -0.32 | -1.29 | -0.01 | -0.81 | -0.16 |
| ZCWPW2\|IGHM | 1.16 | -0.48 | -2.30 | -1.89 | -1.03 | 5.39 |
| FAM129C | 1.15 | 0.09 | -0.37 | -0.34 | -0.11 | 4.98 |
| KIAA0513 | 1.15 | -0.33 | 0.43 | -0.16 | -0.52 | -1.33 |
| RASA4B | 1.15 | -0.65 | -0.04 | 0.20 | -0.93 | -0.53 |
| CD300LF | 1.14 | -0.21 | 1.27 | -1.28 | -1.25 | -1.17 |
| LDLR | 1.14 | 0.90 | 0.33 | 0.26 | -0.02 | -0.98 |
| SRGN | 1.14 | 0.65 | 0.81 | -0.19 | -0.66 | -2.22 |
| ZDHHC7 | 1.13 | -0.18 | -0.19 | -0.68 | -1.04 | -1.31 |
| ATP6V0A1 | 1.11 | -0.07 | 1.01 | -0.62 | -1.44 | 0.77 |
| SPI1 | 1.11 | 0.09 | 1.28 | -1.16 | -1.02 | -0.25 |
| ALDH2 | 1.11 | 0.11 | 0.93 | -0.48 | -0.90 | -0.46 |
| BSG | 1.10 | 0.02 | -0.40 | -0.37 | -0.91 | -1.26 |
| FCER2 | 1.10 | 0.51 | -0.29 | -0.49 | -0.30 | 4.37 |
| ETS2 | 1.10 | 0.71 | 1.70 | -0.20 | -0.55 | -1.26 |
| IL1B | 1.09 | 2.96 | 3.91 | 0.47 | -0.07 | 0.00 |
| CD22 | 1.09 | -0.02 | 0.24 | -0.73 | -0.62 | 5.56 |
| ANXA5 | 1.09 | 1.02 | 1.65 | -0.13 | -0.72 | -1.22 |
| MEFV | 1.07 | -0.07 | 1.50 | -0.80 | -0.81 | -0.95 |
| ALDOA | 1.07 | -0.11 | -0.06 | -0.51 | -0.83 | -1.02 |
| ATP6V1B2 | 1.06 | 0.86 | 1.95 | -0.20 | -0.66 | -1.09 |
| GAPDH | 1.06 | 0.13 | 0.37 | -0.38 | -0.56 | -1.04 |
| IRF2BP2 | 1.02 | -0.86 | 0.07 | -0.70 | -1.61 | -2.25 |
| MT1X | 1.02 | -0.32 | -1.11 | -0.36 | 0.03 | -1.03 |
| PLK2 | 1.01 | 0.40 | 3.75 | 0.24 | 0.49 | 0.10 |
| PTTG1IP | 1.01 | 0.04 | 0.20 | -0.32 | -0.91 | -1.09 |
| DUSP6 | 1.01 | 0.72 | 1.61 | -1.00 | -0.83 | -0.91 |
| MIF | 1.01 | -0.84 | 0.07 | -0.60 | -0.88 | -1.11 |
| IL17RA | 1.00 | 0.02 | -0.05 | -0.01 | -0.30 | -1.01 |
| GOLGA8B\|GOLGA8A | 1.00 | 2.71 | -1.41 | 2.65 | 3.01 | 2.45 |
| SNAI1 | 0.99 | 0.51 | 4.51 | -0.08 | 0.17 | 0.00 |
| RNF24 | 0.99 | -0.04 | 0.64 | -0.32 | -0.66 | -1.36 |
| NCF1C | 0.99 | -0.08 | 0.56 | -1.06 | -1.24 | 2.28 |
| ITM2B | 0.98 | -0.81 | -0.72 | -0.01 | -1.15 | -1.96 |
| ADAM28 | 0.97 | 0.73 | 0.79 | -0.25 | -0.08 | 4.26 |
| HIST1H2BG | 0.97 | -0.45 | 3.75 | -0.15 | 0.34 | -0.36 |
| LOC644090 | 0.97 | 1.52 | 3.53 | 0.12 | -0.01 | -0.10 |
| ADRBK2 | 0.97 | -0.28 | 0.23 | -1.43 | -1.33 | 1.25 |
| BTK | 0.96 | -0.32 | 0.17 | -0.94 | -0.77 | 3.11 |
| IGHM\|IGHG3 | 0.96 | -0.41 | -0.30 | -0.22 | -0.04 | 5.02 |
| HLA-DMB | 0.95 | -0.57 | 0.07 | -1.19 | -0.95 | 2.14 |
| ATP6V0E1 | 0.94 | 0.18 | -1.09 | -0.12 | -1.10 | -0.99 |
| HIST1H2AG | 0.94 | -0.87 | 3.30 | 0.42 | -0.21 | -0.63 |
| CD79A | 0.93 | -0.70 | -1.31 | -1.08 | -0.79 | 4.65 |
| OLR1 | 0.93 | 3.24 | 4.33 | 0.44 | 0.93 | 0.54 |
| IGLC7 | 0.93 | -0.44 | -0.26 | -0.42 | -0.21 | 3.78 |
| ITGAX | 0.92 | 1.69 | 1.10 | -1.95 | -1.41 | -0.67 |
| IGKV3D-15\|IGKV2-29\|IGKJ3 | 0.92 | -0.34 | -0.80 | -0.82 | -0.51 | 4.92 |
| CITED2 | 0.92 | -0.41 | -0.32 | -0.44 | 0.03 | -1.20 |
| IGHG1\|IGHM\|IGHV4-31\|IGHG3 | 0.91 | -0.08 | 0.03 | -0.17 | -0.06 | 3.87 |
| HIST1H4E | 0.91 | -0.48 | 1.72 | -0.02 | -0.52 | -1.18 |
| HIST1H2BH | 0.90 | 0.15 | 3.96 | -0.56 | 0.06 | -0.43 |
| APOBR | 0.90 | 0.17 | 0.37 | -0.82 | -0.53 | -1.12 |
| TNFSF8 | 0.89 | -0.63 | -0.85 | 1.58 | 1.10 | -1.54 |
| GOLGA8A | 0.89 | 2.63 | -1.52 | 2.55 | 2.92 | 2.41 |
| PDE4A | 0.89 | 1.01 | 0.28 | -0.49 | -0.26 | -1.15 |
| IGKV2D-28\|IGKV2-28 | 0.88 | -0.17 | -0.16 | -0.27 | 0.05 | 3.66 |
| RAP2B | 0.88 | -0.37 | 0.62 | -0.42 | -0.68 | -1.28 |
| GLIPR2 | 0.88 | 0.02 | -0.11 | -0.80 | -1.29 | -1.49 |
| EGR1 | 0.88 | 0.08 | 2.97 | -0.51 | -0.07 | 1.17 |
| METRNL | 0.86 | 0.44 | 0.90 | -1.64 | -0.95 | -1.93 |
| CD300A | 0.85 | 1.59 | -0.08 | -1.83 | -0.64 | -1.98 |
| CTSS | 0.85 | 0.46 | 0.77 | -0.85 | -1.30 | -0.30 |
| PAX5 | 0.85 | -0.10 | -0.06 | -0.23 | -0.09 | 4.42 |
| TNFRSF1B\|MIR4632 | 0.85 | 0.14 | 0.24 | -1.07 | -1.50 | -2.16 |
| IGHD | 0.85 | -0.09 | -0.70 | -0.62 | -0.35 | 5.19 |
| CYBB | 0.84 | 1.51 | 0.69 | -1.28 | -1.83 | 1.05 |
| IL6R | 0.84 | -0.83 | 0.81 | 0.07 | -1.22 | -1.76 |
| TNFAIP6 | 0.83 | 4.07 | 3.29 | 1.10 | 0.68 | 0.42 |
| IGHV3-48\|IGHM\|IGHV4-31\|IGHA1\|IGHJ3\|IGHA2\|IGHG1 | 0.83 | -0.41 | -1.25 | -1.02 | -0.39 | 5.41 |
| STAB1 | 0.82 | 0.71 | 3.42 | -0.07 | -0.13 | -0.14 |
| IGKV2-30\|IGKC | 0.81 | -0.10 | -0.18 | -0.23 | -0.10 | 3.34 |
| AGTRAP | 0.81 | -0.27 | -0.02 | -1.20 | -1.20 | -1.37 |
| IER5 | 0.81 | 0.32 | 2.98 | 0.35 | 0.27 | -0.29 |
| IGKV2-24 | 0.81 | -0.22 | -0.48 | -0.54 | -0.09 | 4.48 |
| PLBD1 | 0.81 | -0.29 | 1.33 | -0.82 | -1.06 | -1.20 |
| BASP1 | 0.80 | 0.32 | 1.04 | -0.42 | -1.61 | -0.49 |
| HIST1H4A | 0.80 | -0.19 | 3.58 | -0.39 | -0.10 | -0.65 |
| FAM101B | 0.80 | -0.23 | -0.93 | -0.48 | -0.29 | -1.36 |
| HIF1A-AS2 | 0.80 | 0.41 | 3.07 | -0.14 | -0.10 | 0.05 |
| IGKV1-17\|IGKC | 0.80 | -0.08 | 0.02 | -0.28 | -0.13 | 4.62 |
| C1orf162 | 0.79 | -0.83 | -0.35 | -1.12 | -1.67 | -0.93 |
| IGHV3-33 | 0.79 | -0.44 | -1.43 | -0.98 | -0.88 | 5.01 |
| RHOB | 0.79 | -0.58 | 2.45 | -1.25 | -0.71 | -0.06 |
| CCL3L1 | 0.78 | 1.66 | 4.20 | 0.17 | -0.08 | -0.25 |
| MPI | 0.77 | -0.80 | -1.56 | -0.03 | -0.02 | -0.37 |
| H3F3AP4 | 0.77 | -0.08 | 0.05 | -0.63 | -1.42 | -0.87 |
| IGKV1-37\|IGKV1D-37 | 0.77 | -0.17 | -0.28 | -0.40 | -0.26 | 3.82 |
| AFF3 | 0.77 | 0.39 | -0.32 | 0.06 | -0.08 | 4.00 |
| RGS2 | 0.77 | -0.84 | 1.94 | -1.25 | -0.53 | -0.62 |
| LY86 | 0.77 | -1.30 | -0.04 | -1.83 | -2.20 | 0.74 |
| CST3 | 0.76 | -0.49 | 0.76 | -1.07 | -1.15 | -1.33 |
| TPP1 | 0.74 | -1.05 | -0.32 | -0.70 | -1.64 | -0.79 |
| TRAV2 | 0.74 | -0.85 | -1.46 | 2.08 | 0.60 | -1.61 |
| FCRL1 | 0.73 | 0.08 | -0.17 | -0.19 | 0.01 | 5.27 |
| HIST2H3A\|HIST2H3C | 0.73 | -0.12 | 3.77 | -0.05 | 0.49 | -0.24 |
| FCRL5 | 0.72 | 0.12 | -0.04 | 0.02 | -0.02 | 4.45 |
| KIAA0226L | 0.72 | 0.25 | -0.02 | -0.30 | -0.20 | 4.14 |
| CXCL8 | 0.72 | 3.31 | 2.95 | 0.91 | 0.40 | 0.77 |
| SYNE1 | 0.72 | 4.07 | -0.07 | 0.58 | 3.25 | 0.94 |
| HLA-DMA\|HLA-DMB | 0.72 | -0.86 | -0.10 | -1.31 | -1.54 | 0.88 |
| PSENEN | 0.72 | -0.26 | -0.94 | -0.89 | -1.99 | -1.75 |
| CLEC17A | 0.72 | -0.12 | 0.08 | -0.24 | -0.21 | 3.24 |
| CD109 | 0.71 | 0.49 | 3.25 | 0.12 | 0.21 | -0.05 |
| GABARAP | 0.71 | -0.27 | -0.32 | -0.69 | -1.72 | -1.43 |
| FOSL2 | 0.71 | 0.53 | 0.25 | -0.39 | -0.36 | -2.34 |
| CCR7 | 0.71 | -0.05 | -1.68 | 1.55 | 0.49 | 0.75 |
| ENO1 | 0.71 | -0.75 | 0.34 | -1.11 | -1.34 | -1.26 |
| VMP1\|MIR21 | 0.71 | -0.15 | 0.28 | -0.60 | -1.46 | -1.38 |
| HIST2H2BE | 0.71 | -0.64 | 2.08 | -1.37 | -0.84 | -1.28 |
| BLK | 0.69 | 0.21 | -0.13 | -0.20 | -0.05 | 3.94 |
| OAZ1\|SPPL2B | 0.69 | -0.28 | -0.22 | -0.58 | -1.56 | -1.25 |
| HIST1H2BF | 0.69 | -0.36 | 2.94 | 0.09 | -0.39 | -0.81 |
| LGALS1 | 0.69 | -0.26 | 0.94 | -1.25 | -1.34 | -1.88 |
| ICOS | 0.69 | -0.29 | -0.44 | 2.75 | 1.47 | -0.47 |
| TNFRSF1A | 0.68 | -0.20 | -0.09 | -0.63 | -0.80 | -1.40 |
| HIST1H2AI | 0.68 | -0.90 | 2.42 | -0.59 | -0.88 | -1.55 |
| IGKV1-6 | 0.68 | -0.06 | -0.12 | -0.19 | -0.20 | 3.88 |
| IGHV4-34\|IGHM | 0.67 | -0.51 | -1.23 | -0.11 | -0.41 | 4.81 |
| IGLV1-40 | 0.67 | -0.20 | -0.18 | -0.30 | -0.25 | 4.01 |
| METTL9 | 0.66 | 0.00 | -0.27 | -0.61 | -1.51 | -1.63 |
| HSPA1B\|HSPA1A | 0.66 | -0.47 | 2.71 | -0.05 | -0.31 | -1.00 |
| SYTL3 | 0.65 | 1.04 | -1.10 | 0.78 | 0.23 | -2.47 |
| UBE2R2 | 0.65 | -0.83 | -1.16 | -0.94 | -2.08 | -1.62 |
| HIST1H3F | 0.65 | -0.80 | 0.25 | -0.96 | -0.32 | -1.51 |
| WDFY4 | 0.64 | -0.25 | -0.14 | -0.64 | -0.32 | 3.97 |
| ADIPOR1 | 0.64 | -0.20 | -0.43 | -0.29 | -1.67 | -1.83 |
| SYNGR2 | 0.64 | -0.77 | -0.82 | -0.55 | -1.38 | 0.10 |
| TXNIP | 0.63 | -1.10 | -0.37 | -1.40 | -1.32 | -0.33 |
| PLEKHG1 | 0.62 | 0.09 | -0.15 | 0.29 | 0.34 | 4.07 |
| PLXND1 | 0.62 | 0.87 | 1.12 | -0.40 | -0.32 | -1.83 |
| SWAP70 | 0.61 | 0.44 | 0.19 | -0.57 | -0.25 | 2.92 |
| EAF1 | 0.61 | 1.19 | 2.86 | 0.10 | 0.03 | -0.07 |
| SOCS3 | 0.61 | 0.26 | 1.65 | -0.57 | -1.10 | -1.83 |
| SYK | 0.61 | -0.23 | 0.10 | -2.14 | -2.12 | 1.20 |
| CD19 | 0.60 | -0.06 | -0.08 | -0.09 | -0.11 | 3.78 |
| IL24 | 0.59 | 4.07 | 1.48 | 1.49 | 0.02 | 0.04 |
| EBF1 | 0.59 | -0.08 | 0.03 | -0.05 | -0.08 | 3.25 |
| IGHV4-31\|IGHV3-15\|IGHG1 | 0.58 | -0.19 | -0.48 | -0.35 | -0.16 | 4.37 |
| PTGS2 | 0.57 | 1.08 | 3.66 | -0.16 | -0.14 | -0.23 |
| EFHD2 | 0.57 | 0.90 | -0.67 | -1.22 | -0.32 | -1.49 |
| GNG7 | 0.56 | -0.69 | -0.83 | -0.36 | -0.82 | 3.20 |
| HCST | 0.56 | -0.22 | -0.43 | -1.09 | -0.49 | -2.23 |
| IGKV1-16 | 0.56 | -0.33 | -0.30 | -0.38 | -0.17 | 3.52 |
| GDE1 | 0.55 | 0.17 | -1.45 | -0.37 | -0.91 | -0.98 |
| STX7 | 0.55 | 0.32 | 0.14 | -0.60 | -0.16 | 2.87 |
| IGKV3D-11 | 0.54 | -0.02 | -0.16 | -0.23 | -0.09 | 3.51 |
| IGKC\|IGKV1-5 | 0.54 | -0.14 | -0.80 | -0.66 | -0.16 | 3.38 |
| UTRN | 0.54 | 3.65 | 0.12 | 1.66 | 3.42 | 3.35 |
| CFP | 0.54 | -0.46 | 0.67 | -0.97 | -1.34 | -1.56 |
| ATHL1 | 0.53 | 1.61 | -1.49 | 1.53 | 2.13 | 0.67 |
| PLCG2 | 0.53 | 1.80 | -0.93 | -1.98 | -0.31 | 2.72 |
| NELL2 | 0.53 | 0.06 | -0.19 | 1.51 | 2.72 | -0.30 |
| ACSL1 | 0.52 | 1.71 | 2.63 | 0.03 | -0.41 | -0.21 |
| IGHG2 | 0.52 | -0.52 | 0.18 | -0.44 | -0.45 | 3.67 |
| SAMHD1 | 0.52 | -0.04 | -1.07 | 0.18 | 0.18 | -2.18 |
| CCRL2 | 0.52 | 0.81 | 2.71 | 0.00 | -0.16 | -0.07 |
| IGKV3D-15\|IGKV3-15 | 0.51 | -0.11 | -0.28 | -0.24 | -0.17 | 3.67 |
| RAP1GAP2 | 0.51 | 2.72 | -0.20 | 1.02 | 1.68 | 0.23 |
| IGKV1D-33\|IGKV1-33\|IGKC | 0.51 | -0.23 | -0.06 | -0.12 | -0.15 | 3.00 |
| RNF125 | 0.50 | 0.97 | -1.87 | 1.52 | 1.32 | -1.04 |
| DLG5 | 0.50 | 3.09 | -0.47 | 0.23 | 1.29 | -0.15 |
| THEMIS2 | 0.50 | 0.27 | -0.05 | -1.54 | -1.45 | -0.99 |
| FTL | 0.49 | 0.05 | 0.55 | -0.45 | -1.55 | -1.57 |
| CAPN3 | 0.49 | 0.53 | -0.06 | 0.73 | 1.19 | 2.86 |
| SERPINE1 | 0.49 | 0.85 | 2.75 | -0.09 | 0.11 | -0.03 |
| ATP6V0C | 0.49 | -0.39 | -0.94 | -0.86 | -1.52 | -1.67 |
| HMGN2P46 | 0.49 | 0.87 | 2.50 | 0.06 | -0.03 | 0.06 |
| LINC00926 | 0.49 | 0.27 | 0.00 | -0.12 | -0.17 | 4.41 |
| ELL2 | 0.49 | -0.12 | 2.69 | 0.31 | -0.29 | 0.02 |
| CTSD | 0.48 | -0.02 | -0.18 | -0.76 | -1.56 | -2.40 |
| ANKRD36B | 0.48 | 2.21 | -0.59 | 2.05 | 3.04 | 2.72 |
| TRAJ35 | 0.47 | -0.93 | -1.61 | 2.25 | 1.93 | -1.14 |
| SUPT4H1 | 0.47 | -0.69 | -1.24 | -0.63 | -1.54 | -1.38 |
| CIITA | 0.47 | -0.18 | -1.07 | -0.94 | -0.19 | 3.36 |
| IGKV3OR2-268 | 0.45 | -0.16 | -0.16 | -0.08 | -0.12 | 2.93 |
| HLA-DRA | 0.45 | -0.42 | 0.07 | -0.58 | -1.79 | 0.50 |
| IGKV2D-24 | 0.45 | -0.25 | -0.23 | -0.22 | -0.16 | 3.22 |
| TNFRSF13C | 0.45 | -0.59 | -0.73 | -0.37 | -0.47 | 3.70 |
| SNX29P1 | 0.45 | 0.10 | 0.15 | -0.09 | -0.14 | 2.47 |
| IGKV3D-15 | 0.43 | -0.15 | -0.20 | -0.07 | -0.13 | 3.30 |
| SNORA11\|MAGED2 | 0.43 | 2.61 | -1.20 | 2.04 | 2.91 | 2.08 |
| CNBP | 0.43 | -0.31 | -1.69 | 0.28 | -0.42 | -0.31 |
| TTN | 0.43 | 0.97 | -0.14 | 1.70 | 2.49 | 3.89 |
| JCHAIN | 0.43 | -0.20 | -0.40 | -0.32 | 0.10 | 4.62 |
| ZYX | 0.42 | -0.85 | -0.07 | -1.00 | -1.16 | -1.86 |
| FHL3 | 0.42 | 0.10 | 1.14 | -1.42 | -0.91 | -1.67 |
| IGKV4-1 | 0.42 | -0.11 | -0.14 | -0.12 | -0.17 | 2.69 |
| ZCWPW2\|IGHV3-23 | 0.42 | -0.16 | -0.38 | -0.30 | -0.23 | 4.35 |
| COX8A | 0.41 | -0.69 | -1.02 | -0.48 | -1.65 | -1.85 |
| VAV2 | 0.41 | -0.18 | 0.12 | -0.42 | -0.40 | 2.48 |
| BLNK | 0.41 | -0.15 | -0.26 | -0.07 | -0.20 | 4.10 |
| HIST1H2BJ | 0.41 | -0.25 | 2.89 | -0.11 | 0.01 | -0.17 |
| CDK14 | 0.40 | 0.46 | 0.50 | -0.02 | -0.05 | 3.37 |
| CD28 | 0.40 | -0.78 | -1.73 | 2.16 | 1.68 | -1.81 |
| FAM89B | 0.40 | -0.73 | -0.91 | -0.59 | -1.47 | -1.66 |
| LOC100506258 | 0.39 | 0.24 | 0.03 | -0.17 | -0.14 | 2.49 |
| RGCC | 0.38 | -0.04 | 0.45 | 1.80 | 1.16 | -2.03 |
| CMTM3 | 0.38 | -0.29 | -1.37 | -0.25 | -0.93 | -1.70 |
| IGLL5\|IGLV3-1 | 0.38 | -0.23 | -0.35 | -0.41 | -0.20 | 3.86 |
| LINC00936 | 0.37 | -0.25 | 2.99 | -0.67 | -0.67 | -0.30 |
| KLHDC3 | 0.37 | -0.75 | -1.70 | -0.32 | -1.01 | -0.97 |
| HIST1H3J | 0.37 | -0.31 | 3.16 | -0.05 | 0.31 | -0.45 |
| IGKV3-11 | 0.36 | -0.63 | -0.41 | -0.64 | -0.54 | 3.53 |
| ITPR2 | 0.36 | 1.61 | 0.43 | 0.69 | 1.79 | 2.42 |
| ZMYM6NB | 0.36 | -1.06 | -2.15 | 0.07 | -1.62 | -1.70 |
| CD72 | 0.35 | 0.81 | -0.30 | -0.32 | -0.01 | 3.03 |
| ATP6V1F | 0.35 | -0.20 | 0.31 | -0.84 | -2.02 | -1.33 |
| GOLGA8Q | 0.35 | 3.04 | -0.09 | 0.26 | 1.04 | 0.84 |
| ECH1 | 0.35 | -0.92 | -2.02 | -0.24 | -1.14 | -1.23 |
| CCL2 | 0.35 | 5.59 | 0.64 | 0.44 | 0.00 | -0.06 |
| VTRNA1-1 | 0.34 | 0.81 | 3.07 | 0.95 | 0.62 | 0.08 |
| HIST1H2BE | 0.34 | -0.19 | 3.87 | -0.22 | 0.35 | -0.22 |
| GOLGA8R | 0.34 | 2.58 | -0.14 | 0.22 | 1.12 | 0.68 |
| IGHV1-18 | 0.33 | -0.06 | -0.38 | -0.42 | 0.02 | 4.84 |
| GOLGA8N\|GOLGA8O\|GOLGA8Q | 0.33 | 3.03 | -0.12 | 0.24 | 1.05 | 0.70 |
| MACF1 | 0.33 | 2.84 | 0.01 | 1.54 | 2.87 | 2.34 |
| GOLGA8O\|GOLGA8N\|GOLGA8Q | 0.33 | 3.10 | -0.10 | 0.27 | 1.12 | 0.76 |
| IGHA1\|IGHV3-66 | 0.33 | 0.00 | -0.06 | -0.13 | -0.06 | 3.38 |
| IGK\|IGKV3-20\|IGKC | 0.32 | 0.06 | 0.02 | -0.20 | -0.11 | 3.19 |
| IGHG4 | 0.31 | -0.20 | -0.05 | -0.24 | -0.25 | 2.72 |
| ADAMTS10 | 0.31 | 2.36 | -0.39 | 0.51 | 1.20 | -0.51 |
| BZRAP1 | 0.30 | 2.65 | 0.07 | 0.06 | 0.93 | -0.19 |
| EZR | 0.30 | -0.44 | -1.98 | 0.60 | 0.63 | 1.48 |
| MMP25-AS1 | 0.30 | 2.75 | -0.58 | 0.84 | 2.17 | -0.16 |
| ITGA5 | 0.29 | 0.57 | 1.71 | 0.22 | -0.16 | -2.02 |
| IGHV5-51\|IGHG1 | 0.28 | -0.18 | -0.25 | -0.17 | -0.22 | 4.05 |
| FCHSD2 | 0.28 | -0.09 | -0.91 | -0.11 | -0.29 | 2.29 |
| GPR84 | 0.27 | 0.98 | 2.64 | -0.09 | -0.03 | -0.13 |
| NOTCH2NL | 0.27 | 0.75 | 2.45 | 0.07 | 0.61 | 2.12 |
| PIK3AP1 | 0.27 | 0.61 | 0.64 | -2.22 | -1.51 | 0.47 |
| SLC7A6 | 0.26 | -0.26 | -2.24 | 1.46 | 0.49 | 0.20 |
| ABCA2 | 0.26 | 2.53 | -1.22 | 0.93 | 1.97 | -0.50 |
| LOC102724714 | 0.26 | 0.00 | 0.08 | 0.14 | -0.06 | 2.93 |
| BACH2 | 0.26 | -0.25 | -2.05 | 0.60 | 0.61 | 2.49 |
| STAT4 | 0.26 | 1.73 | -1.09 | 1.48 | 2.27 | -0.73 |
| TUBB | 0.26 | -0.85 | -1.09 | -0.74 | -2.00 | -2.18 |
| USP6NL | 0.25 | 0.12 | -0.23 | -0.23 | -0.09 | 3.02 |
| RALGPS2 | 0.25 | 0.43 | -0.14 | -0.09 | 0.06 | 5.03 |
| ITGAV | 0.25 | 0.81 | 2.68 | 0.15 | 0.41 | 0.02 |
| CD5 | 0.25 | -1.03 | -3.30 | 1.65 | 1.07 | -1.90 |
| TGFBR2 | 0.25 | -0.54 | -2.29 | 0.13 | 0.12 | 0.44 |
| ITPR1 | 0.25 | 0.99 | -0.30 | 0.69 | 1.38 | 3.40 |
| ARHGAP24 | 0.24 | 0.01 | 0.23 | -0.09 | -0.14 | 2.41 |
| MZB1 | 0.24 | -0.21 | -0.31 | -0.33 | -0.05 | 2.57 |
| RASGRP3 | 0.24 | 0.11 | 0.02 | 0.20 | 0.21 | 2.69 |
| IGKV1D-33\|IGKV1-33 | 0.24 | -0.19 | -0.05 | -0.17 | -0.23 | 2.70 |
| MIR4451 | 0.24 | -0.15 | 0.01 | -0.10 | -0.11 | 3.09 |
| HLA-DOA | 0.24 | -0.46 | -0.74 | -0.57 | -0.35 | 2.44 |
| SMCHD1 | 0.23 | 0.56 | -0.10 | 1.39 | 1.97 | 2.40 |
| BANK1 | 0.23 | 0.13 | -0.03 | -0.05 | 0.02 | 4.81 |
| CCDC141 | 0.22 | 0.62 | -0.15 | 1.45 | 2.26 | 3.65 |
| IGKV1-5 | 0.21 | 0.01 | -0.05 | -0.07 | -0.09 | 3.12 |
| IL18RAP | 0.21 | 3.65 | -0.22 | 0.18 | 0.85 | -0.32 |
| VPS13B | 0.21 | 1.85 | 0.11 | 0.92 | 2.10 | 2.56 |
| IGKV2-40 | 0.21 | -0.02 | -0.04 | -0.07 | -0.02 | 2.30 |
| LOC102724162 | 0.20 | -0.04 | 0.05 | -0.04 | -0.01 | 2.42 |
| OLIG1 | 0.20 | 1.07 | 3.18 | -0.19 | -0.15 | -0.37 |
| IGHV1-69\|IGHJ2\|IGHV4-31\|IGHG1\|IGHA1\|IGHD | 0.20 | -0.10 | -0.14 | -0.35 | -0.10 | 4.35 |
| NOMO2\|NOMO1\|NOMO3 | 0.19 | 0.26 | -1.93 | -0.03 | 0.05 | -0.07 |
| NCAM1 | 0.19 | 2.27 | 0.04 | -0.01 | 0.03 | -0.03 |
| MIR4738\|H3F3B | 0.18 | 0.02 | 2.99 | 0.06 | 0.06 | -0.01 |
| EML6 | 0.18 | 0.10 | -0.02 | 0.04 | 0.15 | 2.90 |
| IGKV3D-20 | 0.18 | -0.04 | -0.20 | -0.22 | -0.33 | 3.13 |
| COL19A1 | 0.18 | 0.01 | 0.00 | -0.02 | 0.00 | 2.24 |
| SLC9A7 | 0.18 | -0.26 | -0.34 | -0.06 | 0.03 | 3.08 |
| IL12RB2 | 0.16 | 2.48 | -0.19 | 0.30 | 0.28 | -0.17 |
| LGALS8-AS1 | 0.16 | -0.04 | 2.33 | 0.05 | 0.02 | 0.05 |
| MIR378I\|TNFRSF13C | 0.15 | -0.54 | -0.78 | -0.26 | -0.62 | 3.09 |
| CCNI | 0.15 | -1.15 | -1.72 | -0.22 | -1.91 | -1.54 |
| PNISR | 0.14 | 0.54 | 0.66 | 0.69 | 1.42 | 2.28 |
| CRTAP | 0.14 | -1.26 | -1.52 | -1.08 | -1.87 | -1.85 |
| FCRL2 | 0.14 | 0.03 | -0.03 | -0.01 | -0.03 | 4.02 |
| SIPA1L3 | 0.14 | 0.22 | -1.25 | -0.24 | 0.13 | 2.33 |
| CCDC50 | 0.14 | 0.45 | -0.70 | -0.24 | -0.25 | 3.17 |
| HLA-DOB | 0.13 | 0.19 | -0.14 | 0.02 | 0.21 | 2.95 |
| SNX2 | 0.13 | 0.41 | -0.17 | 0.06 | 0.23 | 2.33 |
| KIAA1109 | 0.13 | 1.88 | 0.42 | 1.19 | 2.43 | 2.07 |
| TNFAIP3 | 0.13 | 0.71 | -0.08 | 1.29 | 1.32 | -1.92 |
| USP24 | 0.12 | 1.60 | -0.10 | 1.00 | 1.98 | 2.51 |
| IGHV4-31 | 0.12 | -0.11 | -0.12 | -0.34 | -0.19 | 3.97 |
| ITPR3 | 0.11 | 1.68 | -2.34 | 1.33 | 1.86 | 1.25 |
| IGKV1D-16 | 0.10 | -0.55 | 0.25 | -0.46 | -0.62 | 2.49 |
| IGHV1-58 | 0.10 | 0.11 | -0.13 | -0.01 | 0.03 | 2.59 |
| IGHM\|IGHV4-31 | 0.10 | 0.23 | -0.21 | -0.18 | 0.05 | 3.90 |
| TBC1D9 | 0.10 | 0.23 | 0.79 | -0.68 | -0.89 | 2.81 |
| IGHV4-61 | 0.10 | -0.14 | -0.57 | -0.20 | 0.01 | 2.76 |
| DENND5B | 0.09 | 0.10 | -0.05 | -0.05 | 0.04 | 2.59 |
| LOC100128059 | 0.08 | 0.29 | 2.53 | -0.08 | 0.10 | 0.00 |
| LOC101926893 | 0.08 | -0.01 | -0.25 | -0.17 | -0.15 | 2.89 |
| KIAA0125 | 0.08 | 0.02 | -0.16 | -0.17 | -0.14 | 2.20 |
| NCOA3 | 0.08 | 0.22 | -0.76 | 0.19 | 0.72 | 2.39 |
| LOC105378056 | 0.08 | 0.00 | -0.03 | -0.02 | -0.02 | 2.35 |
| MICAL3 | 0.07 | 0.47 | -0.63 | 0.22 | 0.49 | 2.76 |
| AMFR | 0.07 | -0.74 | -2.12 | -0.73 | -1.26 | 0.40 |
| CELSR1 | 0.07 | 0.46 | 0.02 | 0.00 | -0.05 | 2.10 |
| IGLV3-25 | 0.07 | -0.21 | -0.35 | -0.33 | -0.06 | 2.22 |
| FANCD2 | 0.07 | 0.50 | -0.26 | 0.55 | 1.16 | 2.32 |
| UGT8 | 0.07 | 0.01 | -0.03 | -0.01 | 0.04 | 2.48 |
| KMO | 0.07 | 1.08 | 1.86 | -0.08 | -0.12 | 2.34 |
| BTLA | 0.06 | -0.22 | -0.20 | 0.37 | -0.15 | 2.57 |
| TNFRSF13B\|TBC1D27 | 0.06 | -0.09 | -0.11 | -0.08 | -0.19 | 2.45 |
| IGLV2-18 | 0.05 | -0.25 | -0.42 | -0.49 | -0.01 | 2.79 |
| SYNE2 | 0.05 | 2.76 | -1.37 | 1.70 | 3.06 | 0.78 |
| IGHM\|IGHV3-7 | 0.05 | 0.02 | -0.05 | -0.02 | -0.03 | 2.55 |
| ZBTB16 | 0.05 | 2.17 | -0.21 | -0.09 | 0.03 | 0.66 |
| ARL5B | 0.04 | 0.24 | 2.36 | 0.18 | 0.07 | 0.37 |
| IL7R | 0.04 | -0.03 | -2.46 | 1.81 | 2.17 | -1.30 |
| RFX3 | 0.04 | 0.99 | -0.55 | 0.66 | 1.44 | 2.07 |
| IGKV1-12\|IGKV1D-12 | 0.04 | 0.03 | -0.28 | -0.05 | -0.08 | 2.71 |
| DMXL1 | 0.04 | 0.43 | 0.63 | -0.01 | 0.46 | 2.32 |
| PLCB1 | 0.04 | 2.25 | 0.48 | 0.19 | 1.24 | -0.15 |
| HSPA1L | 0.04 | -0.27 | 2.12 | -0.27 | -0.22 | -0.47 |
| IGHV1-24\|IGHG1\|IGHG3 | 0.03 | -0.10 | -0.08 | -0.13 | 0.07 | 2.65 |
| TSPAN13 | 0.03 | 0.15 | -0.15 | -0.07 | -0.14 | 2.39 |
| IGHG1\|IGHV4-28 | 0.03 | -0.04 | -0.22 | -0.06 | -0.05 | 2.47 |
| ITSN2 | 0.03 | 0.93 | 0.38 | 0.82 | 1.34 | 2.14 |
| HIST1H2AJ | 0.03 | -0.66 | 4.30 | -0.08 | -0.16 | -0.98 |
| STRBP | 0.03 | -0.03 | -0.10 | 0.02 | 0.16 | 2.29 |
| SETBP1 | 0.03 | 0.65 | -0.24 | -0.23 | 0.28 | 2.42 |
| PTBP1 | 0.03 | -0.11 | -2.18 | 0.21 | -0.15 | -0.47 |
| GANC | 0.02 | 0.01 | -0.38 | 0.28 | 0.66 | 2.87 |
| FOSB | 0.01 | 0.84 | 2.11 | 0.93 | 1.77 | 0.27 |
| IGHV2-26 | 0.01 | -0.06 | -0.04 | -0.03 | -0.07 | 2.51 |
| ADGRG1 | 0.01 | 2.71 | -1.40 | -1.13 | 1.13 | -0.89 |
| KBTBD8 | 0.00 | 0.15 | 2.12 | -0.01 | 0.18 | 1.11 |
| SCAPER | 0.00 | 1.37 | -0.17 | 0.76 | 1.97 | 2.33 |
| BCL11A | 0.00 | -0.26 | -0.14 | -0.41 | -0.38 | 2.15 |
| CR2 | 0.00 | -0.06 | -0.04 | 0.05 | 0.04 | 2.17 |
| GEN1 | 0.00 | 0.04 | -0.03 | 0.01 | 0.13 | 2.31 |
| INADL | 0.00 | 0.79 | -0.57 | 1.37 | 2.31 | 1.66 |
| DUS2 | 0.00 | -0.20 | -0.85 | -0.38 | -0.32 | 2.03 |
| VPREB3 | -0.01 | -0.09 | 0.13 | -0.10 | 0.00 | 2.17 |
| CHD7 | -0.01 | 0.43 | -0.23 | 0.67 | 0.66 | 2.42 |
| BTAF1 | -0.02 | 1.12 | 2.31 | 0.59 | 1.60 | 2.32 |
| IL1A | -0.02 | 1.74 | 3.35 | 0.00 | 0.03 | 0.09 |
| SPAG9 | -0.02 | 0.67 | 2.54 | 0.18 | 0.63 | 0.50 |
| IGHV3-13 | -0.02 | -0.03 | -0.14 | -0.12 | -0.08 | 3.00 |
| IGKV2-29 | -0.02 | -0.06 | -0.07 | -0.06 | -0.01 | 2.02 |
| GOLGA8J | -0.02 | 2.89 | 0.29 | 0.21 | 0.89 | 0.53 |
| VPS41 | -0.03 | 0.91 | 0.18 | 0.28 | 1.06 | 1.98 |
| HES1 | -0.03 | 0.19 | 2.97 | 0.01 | 0.46 | 1.04 |
| RCAN3 | -0.03 | -1.23 | -2.31 | 1.46 | 0.60 | -0.67 |
| DDIT3 | -0.03 | 0.18 | 2.04 | 0.11 | 0.29 | 0.22 |
| STAP1 | -0.04 | 0.30 | -0.18 | -0.05 | -0.14 | 3.23 |
| ZAP70 | -0.04 | 2.07 | -1.80 | 1.07 | 1.74 | -1.68 |
| GPCPD1 | -0.04 | 0.27 | 2.14 | 0.36 | 0.52 | 0.70 |
| KIAA1033 | -0.04 | 0.28 | 0.66 | 0.00 | 0.40 | 2.46 |
| PARP14 | -0.04 | 0.87 | 0.56 | 0.12 | 1.03 | 2.84 |
| PIKFYVE | -0.04 | 0.79 | -0.13 | 0.42 | 1.28 | 2.48 |
| LPAL2 | -0.05 | 2.04 | 0.13 | 0.17 | 0.89 | 0.10 |
| VGF | -0.05 | 0.21 | 2.70 | -0.05 | 0.11 | 0.03 |
| CEP78 | -0.05 | 2.17 | -0.22 | 0.17 | 1.21 | 0.40 |
| P2RY8 | -0.07 | -0.18 | -2.25 | 0.36 | 0.19 | -0.24 |
| LUC7L3 | -0.07 | 0.86 | 0.63 | 0.49 | 1.37 | 2.19 |
| IGLV2-11 | -0.08 | -0.18 | -0.18 | -0.21 | -0.18 | 2.05 |
| MDN1 | -0.08 | 1.35 | -0.62 | 1.28 | 1.95 | 1.56 |
| NR4A1 | -0.08 | -0.47 | 2.27 | -0.49 | -0.54 | -0.29 |
| PPIP5K1 | -0.08 | 1.36 | -0.76 | 0.67 | 1.73 | 1.99 |
| CCL3 | -0.08 | 1.70 | 3.04 | 0.32 | -0.14 | -0.26 |
| MLC1 | -0.08 | 1.98 | -0.14 | -0.57 | -0.12 | -0.51 |
| SECISBP2L | -0.09 | 0.55 | -0.39 | 0.17 | 0.87 | 2.11 |
| HIST2H2BC | -0.09 | -0.98 | 2.56 | -0.57 | -0.99 | -1.00 |
| PHKB | -0.09 | 0.58 | -0.40 | 0.09 | 0.80 | 2.11 |
| TTC37 | -0.09 | 1.14 | -0.26 | 0.66 | 1.68 | 2.00 |
| PNPLA8 | -0.09 | 0.33 | 2.47 | 0.08 | 0.17 | 1.15 |
| IRAK2 | -0.09 | 1.04 | 2.12 | -0.15 | -0.85 | 0.08 |
| LOC105372674 | -0.09 | 1.94 | -0.20 | 0.01 | 0.84 | -0.28 |
| PHIP | -0.10 | 1.13 | 0.53 | 0.45 | 1.67 | 2.01 |
| ZFC3H1 | -0.10 | 0.61 | 2.39 | 0.36 | 0.86 | 1.65 |
| DNAJA4 | -0.10 | -0.14 | 2.08 | 0.34 | -0.16 | -0.33 |
| MYCBP2 | -0.10 | 1.28 | -0.13 | 0.97 | 1.93 | 2.62 |
| BAG3 | -0.10 | -0.31 | 2.90 | 0.29 | -0.24 | -0.63 |
| MGAT5 | -0.11 | 0.10 | -1.21 | 0.31 | 0.31 | 1.92 |
| AKAP9 | -0.12 | 0.77 | 0.23 | 0.41 | 1.41 | 2.09 |
| GTF3C2 | -0.12 | -0.28 | -2.12 | -0.02 | -0.33 | 0.17 |
| IRF8 | -0.13 | -0.37 | 0.28 | -2.18 | -1.65 | 2.46 |
| CRYBB2P1 | -0.13 | 0.29 | 2.51 | 0.16 | -0.01 | 0.12 |
| MBD4 | -0.13 | 0.57 | 0.89 | -0.21 | 0.02 | 1.96 |
| VPS13C | -0.13 | 1.63 | 0.33 | 0.55 | 1.94 | 2.78 |
| ITK | -0.14 | 0.72 | -2.07 | 1.66 | 2.32 | -2.07 |
| CDCA7L | -0.14 | -0.19 | -0.09 | 0.05 | 0.08 | 2.21 |
| UVRAG | -0.14 | 0.10 | -0.49 | -0.38 | 0.09 | 2.08 |
| SKIV2L2 | -0.14 | 0.90 | -0.27 | 0.81 | 1.45 | 1.89 |
| NIN | -0.14 | 0.85 | -0.69 | 0.25 | 1.82 | 2.39 |
| CD180 | -0.15 | -0.18 | 0.04 | -0.88 | -0.72 | 2.56 |
| DENND4A | -0.15 | 0.31 | 0.42 | 0.26 | 1.03 | 2.06 |
| TRGV2 | -0.15 | 0.60 | -0.38 | -0.24 | 1.88 | -0.33 |
| LRBA | -0.16 | 1.72 | -0.90 | 1.18 | 2.51 | 2.62 |
| BOD1L1 | -0.16 | 0.82 | 0.71 | 0.32 | 1.37 | 2.04 |
| ATP2B1 | -0.16 | 0.75 | 2.20 | 0.18 | 1.04 | 2.42 |
| TTC38 | -0.16 | 1.92 | -0.85 | -0.79 | -0.01 | -0.68 |
| AIM2 | -0.16 | -0.06 | -0.04 | -0.09 | -0.04 | 2.99 |
| BCLAF1 | -0.17 | 0.78 | 0.47 | 0.88 | 2.07 | 2.14 |
| DOCK10 | -0.17 | 1.78 | -0.15 | 1.37 | 2.58 | 2.29 |
| BDP1 | -0.17 | 1.26 | -0.49 | 0.56 | 1.88 | 2.30 |
| IFT57 | -0.17 | 0.45 | -0.09 | 0.38 | 0.60 | 2.20 |
| SPTAN1 | -0.17 | 0.85 | -2.29 | 1.01 | 1.25 | 0.44 |
| PRKX | -0.17 | 0.17 | -2.41 | 0.21 | 0.35 | -0.30 |
| PLCG1 | -0.18 | 0.96 | -2.45 | 1.56 | 2.12 | -0.43 |
| SMC6 | -0.18 | 0.36 | -0.23 | 0.17 | 0.71 | 2.87 |
| SP140 | -0.19 | 0.33 | -0.97 | 0.42 | 0.65 | 2.25 |
| HIF1A | -0.19 | 1.23 | 1.04 | -0.12 | 0.61 | 1.81 |
| BCL2A1 | -0.19 | 0.28 | 2.24 | -0.38 | -0.43 | -0.15 |
| PRKCQ | -0.20 | 1.33 | -2.44 | 1.43 | 2.02 | -2.25 |
| LOC102724850 | -0.20 | 1.12 | 2.69 | -0.10 | -0.28 | -0.36 |
| SNORD116-1 | -0.20 | -1.23 | -2.34 | 0.80 | -0.70 | -1.02 |
| CHORDC1 | -0.21 | 0.27 | 2.07 | 0.21 | 0.63 | 0.66 |
| PCM1 | -0.21 | 1.01 | -0.33 | 0.53 | 1.92 | 1.97 |
| IGHV1-2\|IGHG1 | -0.22 | -0.17 | -1.18 | -0.40 | -0.28 | 3.59 |
| PARP15 | -0.22 | 1.32 | -1.12 | 0.66 | 1.62 | 3.46 |
| ADAM19 | -0.22 | -0.87 | -0.57 | 1.42 | 0.48 | 2.77 |
| LOC101928354 | -0.22 | 2.10 | -0.30 | 0.34 | -0.25 | 1.64 |
| MATK | -0.23 | 1.82 | -0.48 | -0.57 | 0.36 | -0.79 |
| KLRC3 | -0.23 | 1.97 | -0.53 | -0.46 | 0.79 | -0.47 |
| CD84 | -0.23 | 0.02 | -0.34 | 1.06 | 2.15 | 1.36 |
| THEMIS | -0.24 | -0.32 | -0.63 | 0.76 | 1.95 | -0.64 |
| JMJD1C | -0.24 | 0.49 | 1.20 | 0.39 | 1.11 | 2.26 |
| TXK | -0.25 | 2.05 | -0.94 | 0.79 | 1.54 | -0.77 |
| TRBV20-1 | -0.25 | -1.65 | -3.36 | 1.71 | 0.75 | -3.27 |
| EIF2AK3 | -0.26 | 0.25 | 0.69 | 0.20 | 0.30 | 2.45 |
| USP9Y | -0.27 | 0.84 | -0.55 | 1.12 | 1.71 | 1.87 |
| RBM26 | -0.27 | 0.72 | -0.85 | 0.83 | 1.33 | 1.96 |
| PXN | -0.27 | 0.81 | -1.50 | 0.19 | 0.19 | -2.49 |
| SPOCK2 | -0.27 | -0.12 | -2.35 | 0.82 | 0.69 | -0.77 |
| MIR4420 | -0.29 | 0.05 | 0.48 | 0.59 | -0.15 | 2.52 |
| SP140L | -0.29 | 0.64 | -0.61 | 0.74 | 1.24 | 2.16 |
| SP100 | -0.29 | 0.73 | -0.15 | 0.24 | 1.26 | 2.42 |
| MAP3K1 | -0.29 | -0.47 | -0.85 | 0.44 | 0.16 | 1.86 |
| SLFN12L | -0.29 | 1.50 | -0.74 | 0.88 | 1.92 | -0.86 |
| LINC00528 | -0.29 | -0.36 | 1.86 | -0.68 | -0.64 | -0.81 |
| DOCK9 | -0.29 | 0.72 | -1.25 | 1.56 | 1.99 | -0.01 |
| FGD2 | -0.30 | -0.36 | 0.21 | -0.48 | -0.27 | 2.79 |
| TRDJ2 | -0.30 | 2.31 | -0.31 | -0.26 | -0.16 | -0.24 |
| FCRL6 | -0.31 | 2.16 | -0.75 | -0.43 | 1.70 | -0.45 |
| BIRC3 | -0.31 | 0.11 | 0.49 | 0.86 | 1.00 | 2.68 |
| HSP90AA1 | -0.31 | 0.38 | 2.60 | 0.25 | 1.16 | 0.66 |
| LOC202181 | -0.31 | 0.18 | -0.07 | 0.33 | 0.24 | 1.79 |
| DMTF1 | -0.31 | 1.17 | 0.84 | 0.47 | 1.56 | 2.28 |
| HEATR5B | -0.31 | 0.79 | -1.07 | 0.55 | 1.12 | 1.93 |
| GVINP1 | -0.32 | 0.45 | -0.77 | 0.42 | 1.02 | 1.85 |
| HNRNPA2B1 | -0.32 | 0.12 | 1.89 | 0.47 | 0.90 | 1.34 |
| RICTOR | -0.32 | 0.90 | 0.21 | 0.46 | 1.49 | 1.74 |
| ZNF532 | -0.32 | 0.29 | -0.64 | -0.37 | 0.23 | 2.18 |
| TPR | -0.32 | 1.18 | -0.03 | 0.81 | 2.17 | 2.04 |
| CCL5 | -0.32 | -0.14 | -1.81 | -2.13 | 0.40 | -3.16 |
| FLT3LG | -0.34 | -0.17 | -2.96 | 1.82 | 1.65 | -1.12 |
| KLRF1 | -0.34 | 2.35 | -0.37 | -0.39 | 0.01 | -0.35 |
| CNTRL | -0.35 | 0.84 | -0.55 | 0.42 | 1.69 | 2.57 |
| QRSL1 | -0.35 | -0.14 | -0.80 | 0.11 | 0.09 | 1.79 |
| TRDJ1 | -0.36 | 2.87 | -0.67 | -0.70 | -0.22 | -0.19 |
| BIRC6 | -0.36 | 0.82 | 0.50 | 0.34 | 1.29 | 1.73 |
| ATRX | -0.36 | 0.71 | -0.10 | 0.25 | 1.21 | 1.74 |
| ARFGAP | -0.37 | 0.71 | 2.04 | -0.21 | -0.21 | -0.34 |
| SEL1L3 | -0.38 | -0.51 | -1.68 | -0.23 | 0.26 | 2.65 |
| MCTP2 | -0.38 | 2.03 | -0.65 | -0.56 | 0.88 | 1.41 |
| DNAJC10 | -0.39 | 0.20 | 0.03 | 0.06 | 0.50 | 2.65 |
| RANBP2 | -0.39 | 0.50 | 2.44 | 0.36 | 1.08 | 0.66 |
| KLRD1 | -0.39 | 1.81 | -0.52 | -0.59 | 0.59 | -0.71 |
| TMEM156 | -0.40 | -0.23 | -0.83 | 0.04 | -0.38 | 1.91 |
| SCIMP | -0.40 | -1.00 | -0.61 | -1.47 | -1.09 | 2.15 |
| IGHD2-15 | -0.40 | -0.10 | -0.52 | -0.40 | -0.03 | 2.38 |
| IQGAP2 | -0.41 | 1.66 | -0.33 | 0.78 | 1.84 | -0.10 |
| LARS | -0.41 | 0.61 | -0.82 | 0.60 | 1.13 | 1.69 |
| SETX | -0.41 | 0.86 | -0.10 | -0.19 | 0.92 | 1.80 |
| CTC1 | -0.41 | 0.18 | -2.79 | 0.77 | 0.90 | 0.82 |
| MYBL1 | -0.41 | 1.69 | -0.91 | 0.11 | 1.48 | -0.76 |
| PARP8 | -0.41 | 1.02 | -0.28 | 0.42 | 1.65 | -0.02 |
| RABEP1 | -0.41 | 0.68 | -1.02 | 0.18 | 0.96 | 2.13 |
| GZMB | -0.42 | 2.01 | -0.92 | -1.02 | 0.10 | -0.96 |
| BCL11B | -0.43 | -1.21 | -2.70 | 0.76 | 0.23 | -2.69 |
| RAD50 | -0.43 | 0.64 | -0.73 | 0.00 | 1.28 | 1.92 |
| HSPH1 | -0.43 | 0.05 | 3.27 | 0.18 | 0.73 | 0.33 |
| TBK1 | -0.43 | 0.43 | 1.72 | -0.29 | 0.08 | 0.30 |
| FCRL3 | -0.45 | 0.39 | -1.50 | -0.78 | 0.40 | 2.87 |
| FAM102A | -0.46 | -2.07 | -3.64 | 1.11 | 0.21 | -0.40 |
| NUP88 | -0.46 | 0.18 | -1.67 | 0.21 | 0.58 | 2.06 |
| USP34 | -0.46 | 0.93 | 0.01 | 0.41 | 1.33 | 1.76 |
| XRN1 | -0.47 | 0.93 | 0.22 | 0.54 | 1.62 | 1.50 |
| SAMD9L | -0.47 | 0.22 | -0.31 | -0.19 | 0.64 | 1.89 |
| HIST3H2BB | -0.49 | -0.06 | 1.67 | -0.13 | 0.11 | -0.62 |
| SLC44A2 | -0.50 | 0.04 | -3.01 | -0.13 | 0.08 | 0.61 |
| ZCCHC7 | -0.50 | -0.17 | -0.88 | -0.15 | 0.18 | 1.98 |
| CCL3L3 | -0.51 | 1.95 | 3.45 | 0.14 | -0.85 | -1.50 |
| TRAF5 | -0.51 | 0.53 | -1.15 | 0.28 | 0.96 | 2.33 |
| CD79B | -0.51 | -0.43 | -1.05 | -0.77 | -0.75 | 2.59 |
| TRDC | -0.51 | 2.84 | -0.95 | -0.60 | -0.23 | -0.57 |
| FAM46C | -0.51 | -0.57 | -2.62 | 0.56 | 0.07 | 0.64 |
| GARS | -0.51 | 0.04 | 1.54 | -0.04 | -0.35 | -0.26 |
| ACAP1 | -0.51 | 1.04 | -2.55 | 0.79 | 1.46 | 0.59 |
| ZFAND2A | -0.53 | -0.18 | 1.75 | -0.40 | -0.23 | -0.45 |
| CHD9 | -0.54 | 1.44 | 0.91 | -0.27 | 1.48 | 2.56 |
| CARD11 | -0.54 | 1.92 | -2.44 | 0.61 | 1.19 | 0.86 |
| PIK3C2B | -0.54 | -0.26 | -1.57 | 0.06 | 0.03 | 1.85 |
| SMC3 | -0.55 | 0.48 | -0.55 | 0.01 | 1.26 | 1.69 |
| KLRC4-KLRK1 | -0.55 | 1.40 | -1.33 | -1.38 | 2.23 | -0.62 |
| P2RY10 | -0.55 | -1.04 | -1.98 | 0.71 | 0.23 | 1.52 |
| S1PR1 | -0.56 | -0.40 | -3.60 | 1.19 | 0.67 | -1.03 |
| SH2D1B | -0.57 | 1.97 | -1.17 | -1.25 | -1.09 | -1.21 |
| PIK3R1 | -0.57 | 0.59 | -1.59 | 0.45 | 1.77 | -0.46 |
| FAM117B | -0.58 | -0.61 | -1.77 | 0.37 | 0.52 | 1.65 |
| ADGRG5 | -0.61 | 1.56 | -0.81 | -0.56 | 0.39 | 0.47 |
| DOCK11 | -0.62 | 0.83 | -0.53 | 0.41 | 1.30 | 1.75 |
| NSUN5P1\|TRIM73 | -0.62 | 0.25 | -1.54 | 0.07 | 0.59 | 1.41 |
| NEAT1 | -0.65 | 0.26 | 2.05 | -0.31 | 0.54 | 0.32 |
| TRAV20\|TRAJ17\|TRDV2\|TRAC | -0.66 | -2.57 | -5.23 | 1.10 | 0.40 | -3.95 |
| ATM | -0.68 | 1.00 | -1.22 | 0.41 | 1.57 | 1.85 |
| CYFIP2 | -0.68 | 0.33 | -2.85 | 0.16 | 0.48 | 0.36 |
| IL2RB | -0.69 | 1.49 | -0.92 | -0.03 | 0.15 | -1.30 |
| TRGV10 | -0.70 | 1.75 | -3.16 | 0.46 | 2.14 | -2.61 |
| GNLY | -0.73 | 2.17 | -1.73 | -1.70 | 0.03 | -1.70 |
| CD2 | -0.74 | -0.66 | -2.61 | 0.82 | 0.58 | -2.89 |
| MAP3K8 | -0.76 | 0.39 | 1.42 | -1.23 | -0.34 | 1.04 |
| CD69 | -0.76 | 0.27 | -1.64 | 0.38 | 1.49 | 1.33 |
| TRBV20OR9-2\|TRBV3-1\|TRBV7-2\|TRBV25OR9-2\|TRBJ2-1 | -0.77 | -2.01 | -3.52 | 0.65 | 0.34 | -2.58 |
| SPTBN1 | -0.77 | 0.70 | -2.03 | 0.74 | 1.12 | 1.41 |
| PYHIN1 | -0.78 | 1.54 | -1.55 | 0.43 | 1.51 | 0.17 |
| ETS1 | -0.82 | 0.48 | -3.60 | 0.34 | 0.67 | -0.14 |
| SP110 | -0.84 | -0.27 | -1.43 | -0.41 | 0.17 | 1.64 |
| HIST1H2AK | -0.86 | -1.40 | 2.59 | -0.88 | -1.57 | -1.91 |
| KIAA0922 | -0.86 | 0.01 | -0.91 | 0.19 | 0.44 | 1.56 |
| PRF1 | -0.87 | 1.92 | -2.69 | -2.20 | 0.36 | -2.26 |
| TC2N | -0.88 | 0.39 | -2.54 | 0.98 | 1.66 | -1.06 |
| CD96 | -0.90 | 0.37 | -2.22 | 1.03 | 1.41 | -0.25 |
| NFATC2 | -0.91 | 0.95 | -3.17 | 0.05 | 0.57 | -0.34 |
| IFI16 | -0.94 | 0.49 | -1.33 | -0.16 | 0.75 | 1.52 |
| TRBV20OR9-2\|TRBJ2-7 | -0.94 | -2.01 | -3.98 | 0.84 | 0.36 | -2.90 |
| KLRB1 | -0.94 | 1.41 | -1.64 | 0.13 | -0.36 | -1.87 |
| ITGA4 | -0.96 | 0.33 | -1.47 | 0.18 | 1.76 | 1.40 |
| CD3E | -0.96 | -1.62 | -3.06 | 0.54 | -0.24 | -3.01 |
| FAM65B | -0.96 | 0.16 | -1.91 | 0.66 | 1.01 | 1.54 |
| TOP2B | -0.97 | 0.29 | 0.09 | -0.27 | 0.85 | 1.21 |
| SLC38A1 | -0.98 | 0.04 | -3.27 | 0.55 | 0.82 | 0.82 |
| CTSW | -1.02 | 1.91 | -2.88 | -1.67 | 0.93 | -2.29 |
| SLAMF7 | -1.04 | 1.57 | -0.07 | 0.25 | -0.05 | -0.40 |
| SKAP1 | -1.10 | 0.55 | -3.25 | 0.60 | 0.85 | -0.70 |
| FCMR | -1.11 | -1.37 | -4.75 | 0.09 | -0.08 | 1.49 |
| TRGJP1 | -1.13 | 1.76 | -1.38 | -0.60 | 1.15 | -1.94 |
| TRBJ2-2 | -1.16 | -1.90 | -3.45 | 0.68 | -0.01 | -3.06 |
| LY9 | -1.18 | -1.77 | -2.28 | -0.47 | -1.15 | 1.06 |
| CST7 | -1.20 | 0.61 | -3.81 | -1.30 | -0.24 | -2.83 |
| TRGJP2\|TRGC2 | -1.25 | 1.05 | -1.14 | -1.51 | 0.49 | -2.45 |
| TRBV3-1\|TRBV7-2\|TRBV6-5\|TRBC2 | -1.29 | -2.14 | -5.85 | 0.36 | -0.13 | -1.98 |
| GZMA | -1.40 | 1.10 | -2.07 | -1.47 | 0.42 | -2.14 |
| TRGC2\|TRGJ1\|TRGJ2 | -1.48 | 0.05 | -3.04 | -0.92 | 1.82 | -2.79 |
| TRGV9\|TRGJ1\|TRGJ2 | -1.48 | 0.05 | -3.04 | -0.92 | 1.82 | -2.79 |
| IKZF3 | -1.48 | -0.08 | -3.55 | -0.25 | 0.29 | 1.20 |
| CCL4 | -1.54 | 0.42 | 0.58 | -0.74 | -1.12 | -1.79 |
| CCL4L2 | -1.56 | 0.75 | 1.19 | -0.53 | -1.04 | -1.81 |
| SLAMF6 | -1.69 | -0.60 | -3.30 | -0.24 | 0.34 | 0.92 |
| NKG7 | -1.71 | 0.75 | -2.97 | -2.97 | -0.23 | -3.23 |
| HIST1H2BM | -1.75 | -1.49 | 1.38 | -1.34 | -1.31 | -2.12 |
