# Supplementary material for: Tissue-regenerative potential of the secretome of γ-irradiated peripheral blood mononuclear cells is mediated via TNFRSF1B-induced necroptosis
Source: Cell Death Dis. 2019 Sep 30;10(10):729. doi: 10.1038/s41419-019-1974-6 (PMC6768878; doi:10.1038/s41419-019-1974-6)
Supplement: Supplementary file 3 — Supplemental table 2 [file 41419_2019_1974_MOESM3_ESM.docx]

| **Supplementary Table 2. Mean expression levels of genes associated with "Cytokine production"** | | | | | | |
| --- | --- | --- | --- | --- | --- | --- |
| **GeneSymbol** | **PBMC** | **NK-cell** | **Monocyte** | **CD4 T-cell** | **CD8 T-cell** | **B-cell** |
| ADAM10 | 403 | 797 | 581 | 360 | 604 | 783 |
| ADAM15 | 61 | 57 | 49 | 51 | 59 | 48 |
| ADAM17 | 258 | 365 | 856 | 213 | 244 | 477 |
| ADAM19 | 198 | 98 | 143 | 741 | 362 | 2071 |
| ADAM28 | 213 | 171 | 173 | 44 | 61 | 2420 |
| ADAM8 | 591 | 433 | 889 | 164 | 279 | 228 |
| ADAM9 | 212 | 356 | 922 | 82 | 79 | 84 |
| ADAMTS10 | 150 | 905 | 63 | 187 | 343 | 55 |
| ADAMTS7 | 91 | 148 | 125 | 103 | 122 | 93 |
| ADAMTSL4 | 57 | 33 | 68 | 28 | 37 | 26 |
| ADAMTSL4-AS1 | 63 | 79 | 94 | 49 | 70 | 47 |
| ADI1 | 114 | 121 | 49 | 112 | 70 | 85 |
| ADIPOR1 | 3105 | 2071 | 1360 | 1547 | 539 | 486 |
| ADIPOR2 | 223 | 137 | 111 | 107 | 111 | 94 |
| ANG | 959 | 109 | 66 | 59 | 46 | 48 |
| ANGPTL6 | 69 | 70 | 52 | 45 | 56 | 71 |
| CCL22 | 1055 | 1449 | 30 | 687 | 463 | 1267 |
| CCL24 | 460 | 200 | 51 | 57 | 66 | 30 |
| CCL3L3 | 387 | 2570 | 7010 | 695 | 274 | 147 |
| CCL4 | 62 | 428 | 558 | 169 | 107 | 41 |
| CCL4L2 | 118 | 806 | 1141 | 322 | 184 | 83 |
| CCL5 | 2108 | 2227 | 692 | 562 | 3271 | 235 |
| CCR1 | 459 | 480 | 217 | 52 | 28 | 19 |
| CCR2 | 558 | 123 | 59 | 39 | 39 | 33 |
| CCR4 | 563 | 24 | 20 | 1882 | 401 | 26 |
| CCR7 | 2016 | 1055 | 292 | 3198 | 1390 | 1857 |
| CCRL2 | 106 | 143 | 700 | 49 | 37 | 44 |
| CCRN4L | 214 | 104 | 208 | 109 | 81 | 52 |
| CSF1 | 77 | 52 | 48 | 69 | 43 | 42 |
| CSF1R | 3280 | 658 | 637 | 241 | 236 | 159 |
| CSF2RA | 1387 | 899 | 1310 | 362 | 299 | 214 |
| CSF2RB | 773 | 290 | 196 | 137 | 133 | 205 |
| CSF3R | 655 | 93 | 835 | 25 | 37 | 33 |
| CXCL16 | 4990 | 1733 | 862 | 574 | 213 | 157 |
| CXCL2 | 128 | 199 | 264 | 82 | 43 | 30 |
| CXCL5 | 542 | 8919 | 1303 | 509 | 97 | 47 |
| CXCL8 | 731 | 5189 | 3133 | 718 | 436 | 612 |
| CXCR2P1 | 177 | 33 | 48 | 19 | 27 | 21 |
| CXCR4 | 11787 | 7111 | 7519 | 9786 | 11487 | 10221 |
| CXCR5 | 81 | 29 | 27 | 75 | 31 | 525 |
| ENG | 2286 | 1105 | 448 | 254 | 237 | 138 |
| FGFBP2 | 94 | 538 | 49 | 58 | 205 | 57 |
| FGFR1 | 68 | 35 | 59 | 44 | 49 | 23 |
| ICAM1 | 530 | 811 | 526 | 211 | 173 | 132 |
| ICAM2 | 874 | 508 | 223 | 1314 | 597 | 518 |
| ICAM3 | 617 | 571 | 195 | 492 | 470 | 568 |
| ICE1 | 77 | 115 | 57 | 131 | 224 | 181 |
| ICE2 | 63 | 55 | 46 | 94 | 100 | 136 |
| ICK | 100 | 159 | 38 | 181 | 215 | 223 |
| ICMT | 114 | 49 | 32 | 108 | 55 | 51 |
| ICOS | 128 | 23 | 14 | 714 | 260 | 13 |
| ICOSLG | 229 | 120 | 212 | 103 | 68 | 593 |
| ICT1 | 197 | 162 | 82 | 235 | 99 | 141 |
| IFNA14 | 55 | 40 | 30 | 62 | 47 | 47 |
| IFNAR1 | 377 | 260 | 119 | 218 | 159 | 329 |
| IFNAR2 | 629 | 389 | 400 | 371 | 245 | 265 |
| IFNGR1 | 334 | 338 | 454 | 110 | 113 | 101 |
| IFNGR2 | 771 | 303 | 465 | 135 | 65 | 229 |
| IL10RA | 2606 | 1685 | 1321 | 1521 | 1904 | 1225 |
| IL10RB | 957 | 440 | 539 | 254 | 114 | 187 |
| IL10RB-AS1 | 402 | 216 | 991 | 112 | 82 | 99 |
| IL11RA | 111 | 163 | 44 | 334 | 274 | 127 |
| IL12RB1 | 78 | 162 | 37 | 104 | 158 | 74 |
| IL13RA1 | 1718 | 916 | 516 | 282 | 244 | 489 |
| IL17RA | 2333 | 1149 | 1042 | 1099 | 865 | 509 |
| IL18BP | 576 | 136 | 926 | 145 | 105 | 119 |
| IL18RAP | 69 | 1378 | 33 | 71 | 151 | 27 |
| IL1B | 224 | 1403 | 1844 | 104 | 49 | 58 |
| IL1R1 | 202 | 159 | 50 | 80 | 59 | 51 |
| IL1R2 | 198 | 43 | 46 | 36 | 30 | 27 |
| IL1RN | 70 | 32 | 190 | 27 | 20 | 20 |
| IL21R | 362 | 328 | 105 | 549 | 465 | 248 |
| IL23A | 263 | 209 | 19 | 583 | 442 | 205 |
| IL24 | 147 | 3265 | 449 | 316 | 52 | 57 |
| IL27 | 80 | 39 | 81 | 35 | 48 | 32 |
| IL27RA | 358 | 321 | 84 | 371 | 217 | 221 |
| IL2RA | 132 | 91 | 85 | 245 | 84 | 272 |
| IL2RB | 138 | 869 | 110 | 255 | 296 | 72 |
| IL2RG | 3278 | 2283 | 1742 | 3403 | 2364 | 2183 |
| IL32 | 81 | 78 | 37 | 188 | 182 | 31 |
| IL3RA | 750 | 373 | 725 | 223 | 308 | 405 |
| IL4I1 | 414 | 234 | 224 | 177 | 118 | 130 |
| IL4R | 1065 | 606 | 811 | 1478 | 640 | 2915 |
| IL6R | 902 | 287 | 874 | 483 | 161 | 91 |
| IL6ST | 309 | 301 | 312 | 785 | 578 | 254 |
| IL7R | 1370 | 902 | 107 | 4036 | 4572 | 303 |
| ILF2 | 578 | 677 | 335 | 780 | 591 | 595 |
| ILF3 | 1665 | 2119 | 747 | 3059 | 2857 | 2272 |
| ILF3-AS1 | 242 | 199 | 316 | 229 | 217 | 185 |
| LCN2 | 65 | 38 | 51 | 52 | 31 | 36 |
| LCOR | 244 | 329 | 244 | 243 | 361 | 483 |
| LCP1 | 4095 | 3490 | 2490 | 2448 | 2645 | 2135 |
| LCP2 | 1649 | 3230 | 3933 | 2741 | 3933 | 508 |
| PDGFB | 136 | 39 | 129 | 47 | 55 | 29 |
| PECAM1 | 348 | 60 | 47 | 22 | 48 | 54 |
| SERPINA1 | 185 | 111 | 200 | 22 | 25 | 19 |
| SERPINE1 | 83 | 105 | 559 | 20 | 33 | 24 |
| TGFA | 61 | 67 | 49 | 52 | 44 | 49 |
| TGFB1 | 2910 | 3563 | 979 | 3593 | 2196 | 936 |
| TGFBI | 310 | 252 | 250 | 86 | 64 | 50 |
| TGFBR1 | 242 | 387 | 402 | 122 | 199 | 194 |
| TGFBR2 | 1323 | 669 | 157 | 1151 | 1076 | 1416 |
| TGFBR3 | 297 | 1215 | 59 | 432 | 762 | 99 |
| TGFBRAP1 | 192 | 266 | 68 | 226 | 217 | 208 |
| TGIF1 | 131 | 113 | 164 | 92 | 94 | 82 |
| TGIF2 | 277 | 228 | 130 | 227 | 161 | 170 |
| TNF | 469 | 324 | 969 | 449 | 242 | 197 |
| TNFAIP1 | 371 | 313 | 264 | 330 | 249 | 227 |
| TNFAIP2 | 451 | 395 | 1901 | 109 | 136 | 136 |
| TNFAIP3 | 3429 | 5164 | 2861 | 7503 | 7690 | 778 |
| TNFAIP6 | 152 | 2443 | 1147 | 186 | 223 | 109 |
| TNFAIP8 | 74 | 73 | 79 | 137 | 84 | 225 |
| TNFAIP8L1 | 81 | 77 | 145 | 83 | 72 | 68 |
| TNFRSF10A | 133 | 70 | 60 | 133 | 96 | 184 |
| TNFRSF10B | 517 | 167 | 321 | 347 | 191 | 214 |
| TNFRSF10C | 203 | 45 | 95 | 58 | 64 | 73 |
| TNFRSF10D | 813 | 522 | 500 | 274 | 77 | 58 |
| TNFRSF11A | 63 | 216 | 42 | 35 | 31 | 18 |
| TNFRSF12A | 142 | 119 | 679 | 87 | 99 | 82 |
| TNFRSF13B | 56 | 42 | 39 | 43 | 34 | 582 |
| TNFRSF13C | 190 | 47 | 39 | 66 | 59 | 2201 |
| TNFRSF14 | 247 | 249 | 555 | 294 | 268 | 244 |
| TNFRSF18 | 57 | 128 | 46 | 89 | 30 | 36 |
| TNFRSF1A | 343 | 178 | 173 | 100 | 81 | 32 |
| TNFRSF1B | 6077 | 4053 | 3718 | 1482 | 1092 | 691 |
| TNFRSF21 | 104 | 44 | 185 | 42 | 39 | 39 |
| TNFRSF25 | 295 | 146 | 62 | 1196 | 396 | 55 |
| TNFRSF9 | 96 | 87 | 81 | 62 | 64 | 34 |
| TNFSF10 | 381 | 117 | 113 | 329 | 136 | 102 |
| TNFSF13 | 193 | 91 | 101 | 61 | 58 | 85 |
| TNFSF13B | 97 | 63 | 95 | 43 | 51 | 41 |
| TNFSF14 | 178 | 354 | 484 | 207 | 161 | 73 |
| TNFSF4 | 102 | 71 | 13 | 92 | 45 | 27 |
| TNFSF8 | 901 | 208 | 198 | 1224 | 858 | 79 |
| TNFSF9 | 167 | 153 | 182 | 113 | 156 | 135 |
| VASP | 1727 | 1437 | 1238 | 782 | 781 | 756 |
| VDR | 306 | 150 | 537 | 83 | 112 | 83 |
| VEGFA | 900 | 251 | 1543 | 64 | 55 | 57 |
| VEGFB | 130 | 57 | 62 | 99 | 51 | 86 |
