# Supplementary material for: Tissue-regenerative potential of the secretome of γ-irradiated peripheral blood mononuclear cells is mediated via TNFRSF1B-induced necroptosis
Source: Cell Death Dis. 2019 Sep 30;10(10):729. doi: 10.1038/s41419-019-1974-6 (PMC6768878; doi:10.1038/s41419-019-1974-6)
Supplement: Supplementary file 5 — Supplemental table 4 [file 41419_2019_1974_MOESM5_ESM.docx]

| **Supplementary Table 4. Mean expression levels of genes associated with "Wound Healing"** | | | | | | |
| --- | --- | --- | --- | --- | --- | --- |
| **GeneSymbol** | **PBMC** | **NK-cell** | **Monocyte** | **CD4 T-cell** | **CD8 T-cell** | **B-cell** |
| ACTA2 | 85 | 27 | 31 | 34 | 28 | 36 |
| ACVRL1 | 18 | 19 | 19 | 17 | 18 | 16 |
| ADAMTS13 | 25 | 28 | 27 | 27 | 24 | 25 |
| ADAMTS18 | 15 | 15 | 15 | 14 | 13 | 14 |
| ADIPOR2 | 223 | 137 | 111 | 107 | 111 | 94 |
| ADRA2A | 11 | 13 | 14 | 16 | 13 | 13 |
| ADRA2C | 25 | 29 | 31 | 19 | 22 | 24 |
| ADRB1 | 52 | 52 | 79 | 42 | 59 | 41 |
| ADRB2 | 486 | 329 | 901 | 118 | 254 | 142 |
| ADTRP | 19 | 17 | 27 | 31 | 22 | 22 |
| AGER | 131 | 92 | 113 | 129 | 111 | 105 |
| AHNAK2 | 19 | 17 | 26 | 23 | 22 | 18 |
| AJAP1 | 19 | 16 | 18 | 20 | 18 | 13 |
| AJUBA | 15 | 14 | 16 | 19 | 15 | 16 |
| ALOX12 | 21 | 19 | 23 | 18 | 15 | 15 |
| ALOX15 | 14 | 16 | 15 | 20 | 16 | 13 |
| ANO6 | 806 | 896 | 638 | 606 | 725 | 669 |
| ANXA1 | 499 | 1032 | 959 | 736 | 1432 | 115 |
| ANXA2P1 | 17 | 18 | 22 | 16 | 15 | 14 |
| ANXA5 | 2342 | 2646 | 3484 | 947 | 631 | 422 |
| ANXA8 | 27 | 33 | 32 | 36 | 28 | 34 |
| AP3B1 | 122 | 265 | 225 | 135 | 284 | 584 |
| APOE | 14 | 15 | 18 | 18 | 16 | 14 |
| APOH | 11 | 10 | 12 | 11 | 12 | 11 |
| AQP1 | 23 | 28 | 29 | 29 | 23 | 26 |
| ARFGEF1 | 139 | 321 | 205 | 257 | 514 | 466 |
| ARHGAP24 | 43 | 28 | 41 | 21 | 18 | 510 |
| ARHGAP35 | 354 | 338 | 106 | 283 | 446 | 195 |
| ARHGEF19 | 26 | 40 | 24 | 36 | 32 | 40 |
| AXL | 34 | 30 | 42 | 33 | 28 | 23 |
| B4GALT1 | 1721 | 1261 | 737 | 722 | 685 | 1128 |
| BCAM | 25 | 30 | 33 | 30 | 35 | 27 |
| BCL9 | 217 | 103 | 47 | 172 | 110 | 85 |
| BIN3 | 171 | 164 | 177 | 137 | 123 | 140 |
| BLOC1S3 | 293 | 211 | 276 | 261 | 243 | 198 |
| BLOC1S4 | 52 | 40 | 37 | 55 | 35 | 39 |
| BLOC1S6 | 174 | 171 | 309 | 168 | 159 | 223 |
| BNC1 | 11 | 10 | 15 | 10 | 10 | 10 |
| C1GALT1C1 | 37 | 28 | 32 | 32 | 23 | 24 |
| C1QTNF1 | 23 | 40 | 24 | 24 | 28 | 25 |
| C3 | 31 | 275 | 169 | 76 | 51 | 35 |
| C9 | 11 | 11 | 11 | 9 | 14 | 11 |
| CAPN3 | 144 | 150 | 75 | 177 | 269 | 993 |
| CARNS1 | 58 | 64 | 67 | 55 | 89 | 119 |
| CASK | 130 | 240 | 48 | 354 | 494 | 295 |
| CAV1 | 37 | 68 | 29 | 36 | 28 | 34 |
| CAV3 | 18 | 14 | 17 | 15 | 17 | 12 |
| CCL2 | 45 | 5577 | 62 | 51 | 17 | 14 |
| CCM2L | 28 | 36 | 44 | 24 | 25 | 31 |
| CCR2 | 558 | 123 | 59 | 39 | 39 | 33 |
| CD109 | 86 | 65 | 949 | 25 | 33 | 15 |
| CD151 | 241 | 169 | 239 | 107 | 110 | 84 |
| CD34 | 18 | 15 | 17 | 15 | 15 | 15 |
| CD36 | 50 | 25 | 39 | 13 | 13 | 11 |
| CD40LG | 61 | 26 | 15 | 234 | 31 | 17 |
| CD44 | 516 | 669 | 501 | 482 | 374 | 260 |
| CD9 | 208 | 55 | 75 | 40 | 28 | 28 |
| CEACAM1 | 40 | 24 | 23 | 28 | 33 | 57 |
| CELSR1 | 27 | 57 | 23 | 23 | 19 | 309 |
| CFLAR | 305 | 578 | 593 | 354 | 394 | 311 |
| CLASP1 | 270 | 600 | 181 | 363 | 474 | 444 |
| CLASP2 | 45 | 79 | 56 | 60 | 78 | 120 |
| CLEC10A | 333 | 78 | 99 | 78 | 65 | 40 |
| CNN2 | 3419 | 2213 | 893 | 2855 | 2079 | 2132 |
| COL1A1 | 54 | 34 | 38 | 57 | 39 | 27 |
| COL3A1 | 14 | 14 | 18 | 17 | 14 | 17 |
| COL5A1 | 27 | 33 | 40 | 28 | 32 | 25 |
| CORO1B | 87 | 67 | 40 | 42 | 39 | 46 |
| CPB2 | 12 | 10 | 13 | 12 | 10 | 9 |
| CRK | 347 | 291 | 245 | 292 | 179 | 247 |
| CRP | 18 | 18 | 27 | 18 | 14 | 16 |
| CX3CL1 | 21 | 19 | 36 | 23 | 34 | 29 |
| CXADRP2 | 11 | 11 | 10 | 10 | 9 | 8 |
| CXCR4 | 11787 | 7111 | 7519 | 9786 | 11487 | 10221 |
| CYR61 | 27 | 22 | 29 | 21 | 29 | 22 |
| DCBLD2 | 15 | 13 | 14 | 14 | 16 | 16 |
| DDR1 | 48 | 55 | 34 | 47 | 41 | 92 |
| DICER1 | 206 | 432 | 261 | 312 | 501 | 809 |
| DMTN | 93 | 72 | 54 | 69 | 114 | 45 |
| DRD5 | 29 | 28 | 34 | 35 | 31 | 31 |
| DTNBP1 | 102 | 186 | 113 | 113 | 121 | 230 |
| DUSP10 | 886 | 1407 | 1219 | 1006 | 1165 | 430 |
| DYSF | 29 | 85 | 50 | 21 | 40 | 18 |
| EGFR | 17 | 15 | 18 | 17 | 14 | 15 |
| ELK3 | 151 | 194 | 171 | 353 | 161 | 143 |
| ENG | 2286 | 1105 | 448 | 254 | 237 | 138 |
| ENPP4 | 24 | 62 | 29 | 50 | 74 | 88 |
| ENTPD1 | 51 | 48 | 68 | 35 | 40 | 185 |
| ENTPD2 | 20 | 24 | 22 | 22 | 22 | 19 |
| EPB41L4B | 18 | 17 | 26 | 17 | 15 | 17 |
| EPPK1 | 31 | 17 | 16 | 57 | 26 | 24 |
| ERBB2 | 48 | 143 | 59 | 70 | 53 | 54 |
| ERBB4 | 12 | 11 | 14 | 14 | 12 | 11 |
| EVL | 189 | 347 | 65 | 439 | 471 | 339 |
| EZH2 | 87 | 131 | 118 | 136 | 139 | 87 |
| F10 | 18 | 23 | 31 | 25 | 20 | 19 |
| F11 | 13 | 13 | 12 | 11 | 14 | 12 |
| F12 | 27 | 31 | 30 | 24 | 24 | 26 |
| F13A1 | 163 | 34 | 112 | 25 | 23 | 24 |
| F13B | 12 | 10 | 12 | 10 | 11 | 11 |
| F2 | 21 | 17 | 21 | 16 | 17 | 13 |
| F2R | 128 | 214 | 97 | 133 | 226 | 49 |
| F2RL1 | 26 | 14 | 18 | 19 | 14 | 15 |
| F2RL2 | 20 | 32 | 27 | 22 | 35 | 21 |
| F2RL3 | 41 | 47 | 65 | 49 | 41 | 40 |
| F3 | 27 | 23 | 65 | 27 | 20 | 18 |
| F5 | 50 | 18 | 25 | 83 | 30 | 14 |
| F7 | 14 | 14 | 24 | 14 | 19 | 17 |
| F8 | 18 | 14 | 14 | 16 | 16 | 13 |
| F9 | 10 | 10 | 12 | 10 | 11 | 11 |
| FBLN1 | 21 | 30 | 38 | 29 | 30 | 25 |
| FCER1G | 5603 | 4166 | 3614 | 948 | 344 | 178 |
| FERMT3 | 633 | 817 | 448 | 447 | 554 | 366 |
| FGA | 13 | 13 | 13 | 13 | 12 | 12 |
| FGB | 12 | 12 | 16 | 13 | 12 | 12 |
| FGF10 | 18 | 13 | 15 | 12 | 11 | 18 |
| FGF2 | 12 | 15 | 14 | 11 | 11 | 11 |
| FGF7 | 13 | 10 | 13 | 12 | 12 | 12 |
| FGFR1OP2 | 36 | 50 | 165 | 46 | 47 | 65 |
| FGG | 12 | 11 | 12 | 10 | 10 | 9 |
| FLNA | 2833 | 4564 | 3347 | 2590 | 3351 | 1436 |
| FN1 | 98 | 16 | 21 | 14 | 15 | 15 |
| FOCAD | 66 | 197 | 46 | 120 | 220 | 133 |
| FOXA2 | 40 | 43 | 50 | 51 | 44 | 42 |
| FOXC2 | 30 | 26 | 34 | 30 | 36 | 23 |
| FZD6 | 29 | 20 | 17 | 42 | 32 | 23 |
| FZD7 | 20 | 26 | 34 | 23 | 19 | 15 |
| FZD9 | 19 | 22 | 23 | 21 | 20 | 29 |
| GAS6 | 48 | 44 | 55 | 53 | 45 | 47 |
| GATA1 | 55 | 47 | 64 | 49 | 55 | 46 |
| GATA4 | 21 | 15 | 19 | 15 | 20 | 19 |
| GJA1 | 13 | 11 | 19 | 11 | 14 | 12 |
| GJD4 | 14 | 15 | 18 | 15 | 15 | 14 |
| GNA13 | 407 | 649 | 661 | 493 | 582 | 522 |
| GNAS | 71 | 62 | 62 | 73 | 66 | 73 |
| GP1BA | 68 | 23 | 33 | 21 | 25 | 23 |
| GP2 | 16 | 21 | 22 | 17 | 17 | 19 |
| GP5 | 34 | 40 | 32 | 30 | 30 | 20 |
| GP6 | 31 | 32 | 54 | 31 | 35 | 29 |
| GP9 | 42 | 32 | 44 | 35 | 32 | 27 |
| GPR4 | 16 | 13 | 34 | 13 | 18 | 17 |
| GPX1 | 1557 | 433 | 821 | 222 | 160 | 163 |
| GRHL3 | 15 | 13 | 18 | 13 | 15 | 14 |
| HBEGF | 221 | 89 | 246 | 35 | 35 | 30 |
| HIF1A | 1011 | 3044 | 2185 | 1205 | 1877 | 4072 |
| HMGB1 | 1979 | 4522 | 1889 | 4131 | 4674 | 4521 |
| HMGCR | 243 | 237 | 182 | 186 | 122 | 158 |
| HMOX1 | 459 | 204 | 694 | 123 | 87 | 100 |
| HNF4A | 22 | 18 | 23 | 20 | 16 | 23 |
| HOPX | 23 | 31 | 28 | 21 | 18 | 18 |
| HPS1 | 239 | 213 | 108 | 191 | 194 | 306 |
| HPS4 | 93 | 115 | 69 | 162 | 137 | 191 |
| HPS5 | 161 | 141 | 245 | 127 | 126 | 284 |
| HPS6 | 24 | 21 | 18 | 26 | 23 | 17 |
| HPSE | 415 | 297 | 219 | 43 | 29 | 25 |
| HRAS | 95 | 123 | 68 | 98 | 106 | 97 |
| HRG | 10 | 9 | 12 | 11 | 11 | 9 |
| IFRD1 | 359 | 323 | 870 | 293 | 327 | 265 |
| IGF1 | 13 | 14 | 16 | 13 | 14 | 15 |
| IGSF10 | 17 | 14 | 15 | 13 | 12 | 15 |
| IL1A | 14 | 365 | 925 | 15 | 17 | 20 |
| INSC | 16 | 19 | 19 | 20 | 17 | 15 |
| INSL3 | 47 | 53 | 40 | 102 | 63 | 46 |
| ITGA2B | 33 | 31 | 50 | 22 | 33 | 30 |
| ITGB3 | 110 | 46 | 83 | 22 | 23 | 33 |
| JARID2 | 1138 | 2232 | 2158 | 983 | 915 | 1196 |
| KANK1 | 14 | 14 | 19 | 15 | 18 | 18 |
| KLKB1 | 11 | 14 | 12 | 15 | 13 | 11 |
| KNG1 | 17 | 17 | 19 | 16 | 18 | 15 |
| KNOP1 | 60 | 149 | 37 | 112 | 144 | 132 |
| KRT6A | 16 | 15 | 22 | 19 | 15 | 14 |
| LARGE-AS1 | 16 | 16 | 13 | 11 | 15 | 15 |
| LGR6 | 57 | 296 | 43 | 39 | 182 | 39 |
| LNX1 | 17 | 18 | 23 | 20 | 22 | 17 |
| LOX | 15 | 13 | 15 | 15 | 12 | 14 |
| LYN | 1686 | 3636 | 1697 | 693 | 456 | 4007 |
| LYST | 252 | 553 | 762 | 178 | 512 | 948 |
| MACF1 | 316 | 2133 | 249 | 861 | 2069 | 1420 |
| MAP3K1 | 585 | 462 | 346 | 1041 | 789 | 2565 |
| MAPK14 | 269 | 274 | 265 | 240 | 228 | 212 |
| MERTK | 35 | 50 | 155 | 21 | 20 | 16 |
| MIA3 | 172 | 240 | 115 | 275 | 450 | 385 |
| MIR489 | 11 | 11 | 20 | 12 | 13 | 11 |
| MIR676 | 12 | 11 | 16 | 10 | 12 | 11 |
| MIR708 | 12 | 12 | 13 | 11 | 11 | 11 |
| MMP12 | 11 | 13 | 12 | 12 | 11 | 11 |
| MMRN1 | 17 | 19 | 20 | 18 | 16 | 16 |
| MPL | 29 | 27 | 29 | 28 | 40 | 29 |
| MSX2P1 | 15 | 38 | 31 | 30 | 30 | 18 |
| MTOR | 422 | 837 | 147 | 754 | 864 | 934 |
| MUC16 | 22 | 20 | 22 | 22 | 31 | 40 |
| MUT | 71 | 91 | 74 | 102 | 112 | 123 |
| MYC | 62 | 58 | 42 | 75 | 101 | 265 |
| MYF6 | 11 | 10 | 16 | 10 | 12 | 14 |
| MYH10 | 16 | 14 | 17 | 16 | 13 | 16 |
| MYH2 | 20 | 11 | 16 | 13 | 18 | 11 |
| MYLK | 16 | 11 | 15 | 14 | 12 | 12 |
| MYNN | 59 | 118 | 93 | 103 | 110 | 151 |
| MYOD1 | 19 | 20 | 26 | 17 | 20 | 16 |
| MYOF | 91 | 69 | 411 | 44 | 53 | 46 |
| NACA | 74 | 74 | 67 | 98 | 85 | 101 |
| NBEAL2 | 717 | 1840 | 798 | 1325 | 1604 | 586 |
| NDNF | 15 | 18 | 17 | 17 | 16 | 17 |
| NF1 | 153 | 230 | 153 | 119 | 164 | 153 |
| NFE2L2 | 231 | 380 | 354 | 231 | 234 | 268 |
| NINJ1 | 491 | 557 | 741 | 257 | 130 | 82 |
| NINJ2 | 69 | 58 | 77 | 70 | 49 | 61 |
| NLRP6 | 31 | 34 | 26 | 30 | 36 | 25 |
| NOG | 54 | 26 | 28 | 97 | 29 | 23 |
| NOTCH2 | 852 | 650 | 1290 | 327 | 422 | 955 |
| NOV | 17 | 18 | 18 | 16 | 16 | 15 |
| P2RX1 | 544 | 136 | 473 | 137 | 178 | 595 |
| P2RX5-TAX1BP3 | 122 | 77 | 81 | 83 | 70 | 150 |
| P2RY1 | 23 | 19 | 42 | 17 | 19 | 15 |
| P2RY12 | 21 | 12 | 15 | 13 | 12 | 10 |
| PAK1 | 389 | 179 | 182 | 117 | 143 | 116 |
| PAPSS2 | 90 | 49 | 38 | 37 | 28 | 26 |
| PARD3 | 18 | 12 | 21 | 12 | 17 | 11 |
| PAX7 | 21 | 20 | 32 | 26 | 24 | 22 |
| PDCD10 | 35 | 50 | 64 | 39 | 51 | 65 |
| PDGFA | 34 | 29 | 42 | 24 | 30 | 24 |
| PDGFB | 136 | 39 | 129 | 47 | 55 | 29 |
| PDGFC | 23 | 26 | 17 | 19 | 16 | 18 |
| PDGFD | 23 | 183 | 14 | 15 | 78 | 22 |
| PDGFRA | 12 | 12 | 12 | 11 | 12 | 11 |
| PDPN | 18 | 35 | 21 | 22 | 16 | 16 |
| PEAR1 | 18 | 17 | 24 | 16 | 21 | 21 |
| PECAM1 | 348 | 60 | 47 | 22 | 48 | 54 |
| PF4 | 235 | 78 | 103 | 41 | 52 | 63 |
| PHLDB2 | 17 | 48 | 17 | 21 | 27 | 19 |
| PIK3CA | 73 | 166 | 76 | 184 | 283 | 423 |
| PIK3CB | 384 | 116 | 302 | 121 | 128 | 126 |
| PIK3CD | 516 | 949 | 260 | 975 | 1274 | 773 |
| PIK3CG | 295 | 770 | 178 | 317 | 587 | 767 |
| PIP5K1C | 299 | 401 | 221 | 261 | 334 | 237 |
| PKM | 3311 | 1661 | 2587 | 899 | 809 | 743 |
| PLAU | 26 | 59 | 99 | 24 | 28 | 35 |
| PLEK | 1594 | 1461 | 2306 | 341 | 687 | 730 |
| PLET1 | 11 | 12 | 12 | 11 | 12 | 10 |
| PLG | 17 | 22 | 23 | 25 | 15 | 17 |
| PLRG1 | 184 | 140 | 88 | 217 | 164 | 196 |
| POU2F3 | 13 | 12 | 13 | 12 | 12 | 11 |
| PPARA | 64 | 77 | 41 | 54 | 49 | 55 |
| PPARD | 542 | 334 | 504 | 336 | 296 | 371 |
| PRCP | 158 | 51 | 42 | 34 | 35 | 44 |
| PRDX2 | 211 | 132 | 87 | 274 | 203 | 99 |
| PRKCA | 207 | 207 | 53 | 602 | 653 | 30 |
| PRKCD | 1144 | 694 | 632 | 353 | 390 | 485 |
| PRKCE | 67 | 77 | 71 | 54 | 57 | 373 |
| PRKCQ | 393 | 1225 | 28 | 1304 | 1950 | 44 |
| PRKG1 | 11 | 11 | 16 | 11 | 13 | 11 |
| PROC | 29 | 25 | 49 | 24 | 26 | 29 |
| PROCR | 26 | 180 | 35 | 40 | 55 | 30 |
| PROS1 | 15 | 16 | 18 | 15 | 13 | 12 |
| PROZ | 24 | 30 | 44 | 29 | 25 | 26 |
| PRSS53 | 59 | 57 | 52 | 54 | 48 | 45 |
| PSG7 | 12 | 9 | 11 | 11 | 9 | 8 |
| PTENP1 | 16 | 12 | 15 | 14 | 12 | 14 |
| PTGER3 | 15 | 14 | 13 | 15 | 14 | 14 |
| PTGER4 | 337 | 272 | 296 | 552 | 503 | 120 |
| PTK7 | 21 | 23 | 26 | 22 | 17 | 14 |
| PTPN6 | 953 | 1199 | 660 | 577 | 1130 | 2412 |
| PTPRJ | 1196 | 1213 | 517 | 522 | 528 | 817 |
| RAB27A | 280 | 285 | 149 | 150 | 197 | 80 |
| RAP2B | 667 | 239 | 526 | 228 | 179 | 99 |
| REG3A | 14 | 11 | 12 | 15 | 11 | 11 |
| REG3G | 19 | 17 | 30 | 22 | 20 | 20 |
| RREB1 | 666 | 546 | 763 | 565 | 408 | 497 |
| S100A9 | 1075 | 804 | 2470 | 106 | 63 | 43 |
| SCNN1B | 15 | 18 | 30 | 19 | 14 | 20 |
| SCNN1G | 19 | 18 | 32 | 19 | 19 | 16 |
| SCRIB | 70 | 129 | 48 | 88 | 102 | 98 |
| SDC1 | 26 | 28 | 35 | 22 | 26 | 26 |
| SDC4 | 802 | 490 | 645 | 122 | 52 | 50 |
| SELK | 106 | 113 | 158 | 108 | 111 | 105 |
| SELP | 24 | 18 | 17 | 22 | 22 | 22 |
| SERPINA10 | 19 | 15 | 19 | 16 | 20 | 18 |
| SERPINB2 | 248 | 2367 | 982 | 93 | 36 | 27 |
| SERPINC1 | 11 | 12 | 15 | 13 | 12 | 12 |
| SERPIND1 | 20 | 19 | 18 | 15 | 15 | 15 |
| SERPINE1 | 83 | 105 | 559 | 20 | 33 | 24 |
| SERPINE2 | 15 | 19 | 19 | 18 | 16 | 17 |
| SERPINF2 | 25 | 33 | 40 | 27 | 33 | 29 |
| SERPING1 | 50 | 31 | 27 | 19 | 23 | 20 |
| SHH | 17 | 15 | 27 | 19 | 18 | 19 |
| SLC11A1 | 1057 | 518 | 1978 | 71 | 68 | 95 |
| SLC7A11 | 125 | 2180 | 1641 | 235 | 459 | 408 |
| SMAD3 | 41 | 42 | 47 | 49 | 47 | 75 |
| SMAD4 | 596 | 589 | 291 | 788 | 608 | 730 |
| SNAI2 | 15 | 16 | 19 | 15 | 16 | 15 |
| SOX15 | 18 | 20 | 26 | 24 | 23 | 23 |
| SPACA1 | 11 | 12 | 13 | 12 | 11 | 11 |
| SRF | 718 | 538 | 430 | 595 | 350 | 345 |
| SRSF6 | 561 | 569 | 657 | 813 | 733 | 964 |
| STARD13 | 17 | 17 | 31 | 14 | 14 | 17 |
| STXBP1 | 129 | 80 | 150 | 88 | 191 | 38 |
| STXBP3 | 132 | 172 | 178 | 137 | 157 | 156 |
| SYK | 709 | 387 | 471 | 52 | 51 | 1095 |
| SYT11 | 348 | 504 | 81 | 397 | 352 | 140 |
| SYT7 | 18 | 15 | 22 | 16 | 18 | 15 |
| TBXA2R | 33 | 28 | 34 | 30 | 38 | 29 |
| TEC | 100 | 118 | 56 | 31 | 49 | 168 |
| TFF1 | 25 | 30 | 30 | 26 | 34 | 30 |
| TFF2 | 14 | 12 | 18 | 13 | 15 | 12 |
| TFF3 | 19 | 15 | 30 | 24 | 18 | 23 |
| TFPI | 22 | 31 | 16 | 18 | 22 | 31 |
| TFPI2 | 12 | 19 | 15 | 15 | 12 | 12 |
| TGFB1 | 2910 | 3563 | 979 | 3593 | 2196 | 936 |
| TGFB2 | 17 | 16 | 24 | 14 | 16 | 24 |
| THBD | 158 | 70 | 100 | 26 | 24 | 21 |
| THBS1 | 10560 | 9690 | 3341 | 1772 | 633 | 342 |
| TIMP1 | 1864 | 871 | 1315 | 470 | 270 | 152 |
| TLR4 | 165 | 37 | 223 | 23 | 30 | 40 |
| TMEFF2 | 11 | 13 | 12 | 12 | 12 | 10 |
| TMPRSS6 | 20 | 25 | 16 | 18 | 22 | 15 |
| TNFRSF12A | 142 | 119 | 679 | 87 | 99 | 82 |
| TOR1A | 551 | 399 | 140 | 348 | 222 | 204 |
| TPM1 | 21 | 20 | 19 | 18 | 23 | 23 |
| TPSB2 | 27 | 36 | 65 | 29 | 30 | 23 |
| TREML1 | 90 | 25 | 48 | 24 | 14 | 17 |
| TRIM72 | 22 | 18 | 20 | 18 | 19 | 19 |
| TSPAN32 | 162 | 505 | 192 | 175 | 404 | 182 |
| TSPAN8 | 12 | 12 | 14 | 12 | 11 | 12 |
| TXK | 109 | 800 | 39 | 309 | 528 | 51 |
| TYRO3 | 16 | 20 | 27 | 25 | 18 | 21 |
| UBASH3A | 166 | 76 | 36 | 526 | 442 | 34 |
| UBASH3B | 67 | 85 | 52 | 94 | 103 | 54 |
| VANGL2 | 19 | 19 | 24 | 20 | 19 | 18 |
| VEGFB | 130 | 57 | 62 | 99 | 51 | 86 |
| VIL1 | 16 | 19 | 18 | 17 | 17 | 16 |
| VKORC1 | 506 | 207 | 204 | 200 | 188 | 120 |
| VPS33B | 163 | 168 | 73 | 194 | 198 | 192 |
| VWF | 23 | 20 | 34 | 25 | 25 | 23 |
| WARS | 165 | 114 | 195 | 69 | 80 | 138 |
| WFDC1 | 15 | 16 | 13 | 18 | 14 | 14 |
| WNT10B | 46 | 72 | 51 | 62 | 107 | 35 |
| WNT3A | 20 | 20 | 23 | 21 | 19 | 20 |
| WNT4 | 18 | 19 | 27 | 16 | 15 | 13 |
| WNT5A | 18 | 14 | 23 | 15 | 16 | 19 |
| WNT7A | 73 | 35 | 39 | 133 | 60 | 34 |
| XBP1 | 403 | 472 | 683 | 582 | 307 | 186 |
